# Diels–Alder cycloadditions of *N*-arylpyrroles via aryne intermediates using diaryliodonium salts

**DOI:** 10.3762/bjoc.14.23

**Published:** 2018-02-06

**Authors:** Huangguan Chen, Jianwei Han, Limin Wang

**Affiliations:** 1 Key Laboratory for Advanced Materials, Institute of Fine Chemicals, School of Chemistry & Molecular Engineering, East China University of Science and Technology, 130 Meilong Road, Shanghai 200237, P. R. China; 2 Shanghai–Hong Kong Joint Laboratory in Chemical Synthesis, Shanghai Institute of Organic Chemistry, The Chinese Academy of Sciences, 345 Lingling Road, Shanghai 200032, China

**Keywords:** benzyne, cycloaddition, diaryliodonium salts, *N*-phenylamine, pyrrole

## Abstract

With a strategy of the formation of benzynes by using diaryliodonium salts, a cycloaddition reaction of *N*-arylpyrroles with benzynes was reported. A wide range of bridge-ring amines with various substituents have been synthesized in moderate to excellent yields (35–96%). Furthermore, with a catalytic amount of TsOH·H_2_O, these amines can be converted into the corresponding *N*-phenylamine derivatives easily, which are potentially useful in photosensitive dyes.

## Introduction

**Scheme 1:**
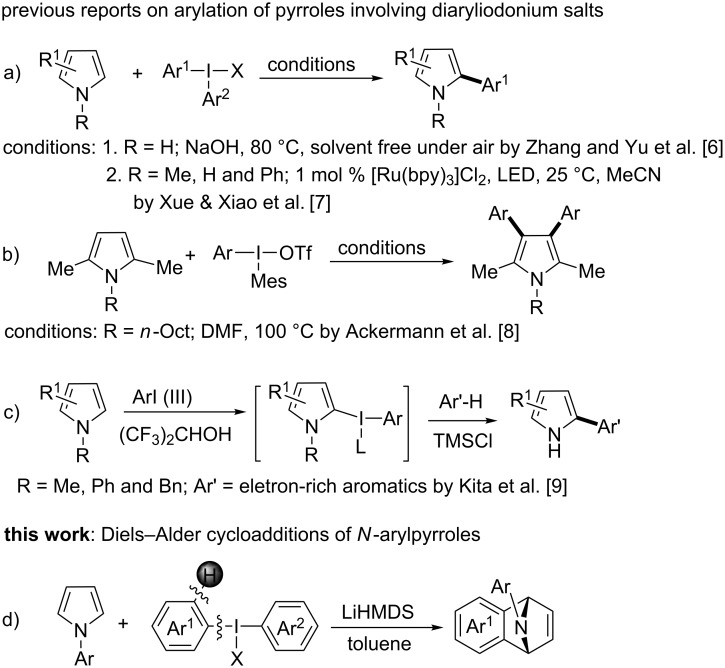
Arylations of pyrrole derivatives with diaryliodonium salts.

Pyrrole is a very useful heterocyclic substrate to produce structural attributes of valuable chemicals, functional materials and pharmaceuticals [1–5]. Recently, arylation of pyrrole derivatives with diaryliodonium salts for pyrrole–aryl coupling products is generating tremendous academic interest in organic synthesis. In 2012, the Zhang and Yu group reported that sodium hydroxide promoted direct arylation of unprotected pyrroles with diaryliodonium salts at the temperature of 80 °C, the coupling products were obtained in moderate to good yields (Scheme 1a) [6]. Later in 2013, Xue and Xiao et al. developed a method of photoredox catalysis in the presence of [Ru(bpy)_3_]^2+^ with visible light for the coupling reaction of arenes with unprotected or N-substituted pyrroles, pyrrole substrates were well tolerated with *N*-methyl and *N*-phenyl groups (Scheme 1a) [7]. Ackermann et al. employed a 2,5-dimethylpyrrole derivative as substrate to deliver double arylated products at 3,4-positions of the pyrrole ring (Scheme 1b) [8]. Recently, the research group of Kita documented an oxidative biaryl coupling for pyrroles using a hypervalent iodine reagent and a stabilizer for pyrrolyliodonium intermediates (Scheme 1c) [9]. The reactions readily provided a variety of desired coupling products in good yields. In general, the mechanism of these arylations was postulated by generating aryl radicals with diaryiodonium salts in the literature.

In 1995, Kitamura prepared phenyl[*o*-(trimethylsilyl)phenyl]iodonium triflate which could be used as an efficient benzyne precursor in trapping furans [10]. Surprisingly, the research groups of Stuart [11–12] and Wang [13] independently discovered in 2016 that simple diaryliodonium salts can generate benzynes under severe basic conditions, the resulted benzynes were allowed to undergo cycloaddition reaction with furan or N-arylation of secondary amides and amines. Due to the easy accessibility of the diaryliodonium salts, this kind of benzyne precursor is attracting extensive attention [14–16]. Also as a five-membered heterocyclic ring, the cycloaddition reaction of N-substituted pyrroles is much less than that of furan [17–21]. As a matter of fact, the Diels–Alder adduct formation of pyrroles with benzyne has been postulated in 1965 as transient products under thermal conditions to afford arylamines [22]. Inspired by the pioneering work of Stuart and Wang [12–13], herein we reported the usage of diaryliodonium salts as aryne precursor for Diels–Alder cycloadditions of *N*-arylpyrroles (Scheme 1d).

## Results and Discussion

We initially started the cycloaddition reaction of 1-phenylpyrrole (**1a**) using phenyl(mesityl)iodonium tosylate (**2a**) as benzyne precursor. To our delight, with LiHMDS as the base in toluene, the Diels–Alder adduct **3aa** was obtained in 23% yield at room temperature (Table 1, entry 1). However, when the reaction temperature was increased to 100 ^o^C or the solvent was changed to THF, we found a slight decrease in the yield of **3aa** (Table 1, entries 2 and 3). Interestingly, the reaction stoichiometry of **1a** and **2a** had a significant influence on the yield of **3aa**, which was similar to Stuart’s work [11–12] (Table 1, entry 4–8). Further examinations of bases did not lead to better results (Table 1, entries 9–14). We then chose LiHMDS as the optimal base for the reaction. The reaction yield could be improved to 85% when an excess amount of LiHMDS was used (Table 1, entries 15–17). However, a screening of reaction temperature, solvent, and reaction time did not improve the yield of **3aa** (Table 1, entries 18–21).

**Table 1:** Optimization of reaction conditions.^a^

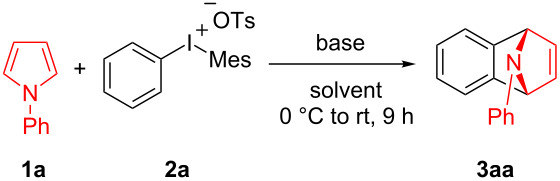				

entry	**1a**/**2a** (equiv)	base (equiv)	solvent	**3aa** (%)^b^

1	1:1.2	LiHMDS (1.2)	toluene	23
2^c^	1:1.2	LiHMDS (1.2)	toluene	22
3	1:1.2	LiHMDS (1.2)	THF	20
4	1:3	LiHMDS (3)	toluene	40
5	3:1	LiHMDS (1)	toluene	59
6	4:1	LiHMDS (1)	toluene	73
7	5:1	LiHMDS (1)	toluene	80
8	6:1	LiHMDS (1)	toluene	74
9	5:1	KHMDS (1.5)	toluene	68
10	5:1	KO*t*-Bu (1)	toluene	60
11	5:1	NaNH_2_ (1)	toluene	39
12	5:1	KO*t*-Bu (2)	toluene	65
13	5:1	NaOMe (2)	toluene	40
14	5:1	NaH (2)	toluene	n. r.
15	5:1	LiHMDS (1.2)	toluene	79
16	5:1	LiHMDS (1.5)	toluene	85
17	5:1	LiHMDS (2)	toluene	74
18^d^	5:1	LiHMDS (1.5)	toluene	70
19	5:1	LiHMDS (1.5)	THF	73
20	5:1	LiHMDS (1.5)	MeCN	n. r.
21^e^	5:1	LiHMDS (1.5)	toluene	73

^a^Reaction conditions: **1a** or **2a** (0.5 mmol, 1 equiv), base (0.5–0.75 mmol, 1–1.5 equiv), solvent (5 mL), 0 °C to rt, 9 h. ^b^Isolated yield. ^c^The reaction temperature was 100 °C. ^d^The reaction temperature was 80 °C. ^e^The reaction was quenched after 13 hours. n. r. = no reaction.

With the optimal reaction conditions in hand, various aryl(mesityl)iodonium salts **2** were examined. As shown in Table 2, an extensive range of substituted aryl(mesityl)iodonium salts, bearing a wide variety of substituent groups, could react with **1a** to afford the corresponding cycloaddition adducts **3**. It was observed that the reaction gave the desired products **3ab** and **3ac** in moderate yields of 63% and 57% when iodonium salts **2** have electron-donating groups in the *para*-position of the aryl moiety, such as methyl groups and *tert-*butyl groups (Table 2, entries 2 and 3). For those bearing electron-withdrawing groups, such as halogens (F, Cl, Br), cyano, nitro, trifluoromethyl, and trifluoromethoxy groups, the corresponding products **3ad**–**3aj** were obtained in good to excellent yields of 67–96% (Table 2, entries 4–10). It was found that the reactions underwent smoothly to give the products **3ak**, **3al** in good yields of 89% and 82%, respectively, when there was a phenyl group on the *para*- or *ortho*-positions of the aryl moiety for diaryliodonium salts (Table 2, entries 11 and 12). Analogous to previous work [11–12], when **2m** and **2n** were employed, the cycloaddition regioselectively afforded **3am** and **3an** in good yields of 80% and 71%, respectively (Table 2, entries 13 and 14). Of note, substituents at the *ortho*-position on the aryl moiety with **2**, regardless of their electronic properties, had a negative effect on the reactivity (Table 2, entries 15–19).

**Table 2:** Scope of diaryliodonium salts **2**.^a^

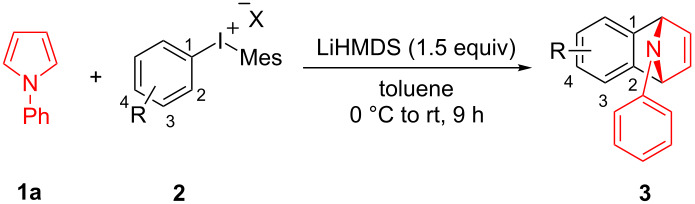			

entry	aryl(mesityl)iodonium salts	product	yield (%)^b^

1	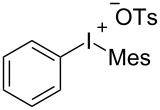 **2a**	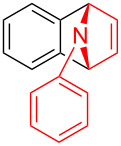 **3aa**	85
2	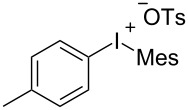 **2b**	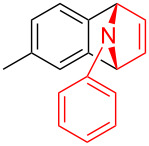 **3ab**	63
3	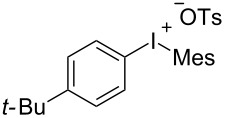 **2c**	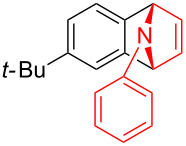 **3ac**	57
4	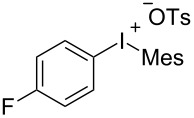 **2d**	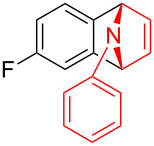 **3ad**	77
5	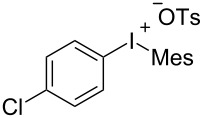 **2e**	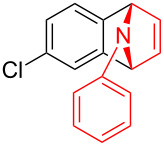 **3ae**	87
6	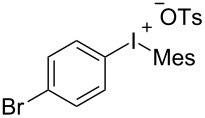 **2f**	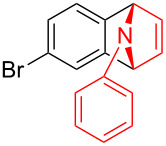 **3af**	96
7	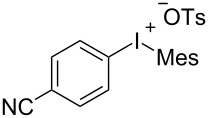 **2g**	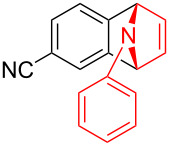 **3ag**	71
8	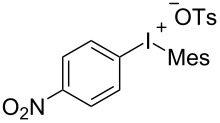 **2h**	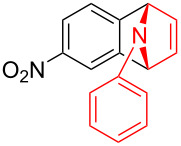 **3ah**	67
9	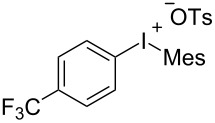 **2i**	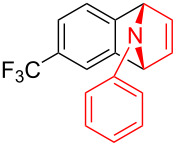 **3ai**	77
10	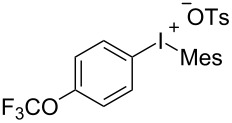 **2j**	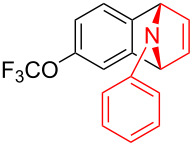 **3aj**	88
11	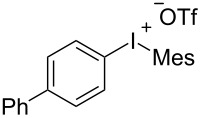 **2k**	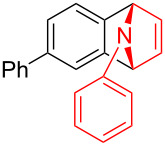 **3ak**	89
12	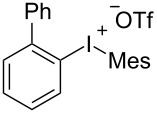 **2l**	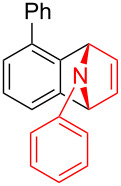 **3al**	82
13	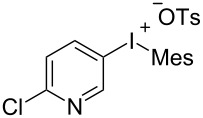 **2m**	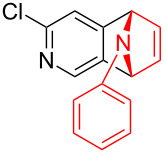 **3am**	80
14	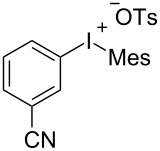 **2n**	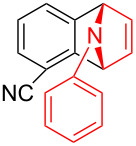 **3an**	71
15	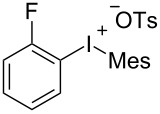 **2o**	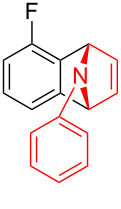 **3ao**	78
16	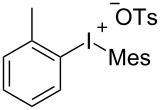 **2p**	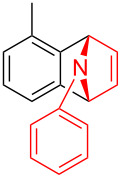 **3ap**	60
17	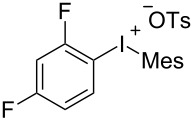 **2q**	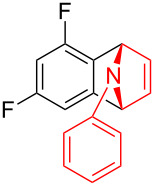 **3aq**	48
18	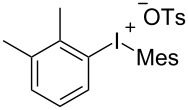 **2r**	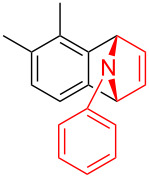 **3ar**	62
19	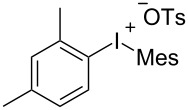 **2s**	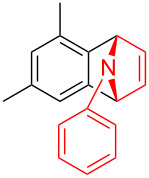 **3as**	58

^a^Reaction conditions: **1a** (2.5 mmol, 5 equiv), **2** (0.5 mmol), LiHMDS (1 M in toluene, 0.75 mL, 1.5 equiv), toluene (5 mL), 0 °C to rt, 9 h. ^b^Isolated yield. Mes = 2,4,6-trimethylphenyl, OTs = 4-toluenesulfonate, OTf = trifluoromethansulfonate.

To further probe the scope of this reaction, a wide range of 1-arylpyrroles **1** was employed in the reaction under the standard conditions. Generally, the conditions proved to be efficient for this Diels–Alder cycloaddition. As shown in Table 3, the electronic properties of aryl substituents had a little influence on the reaction outcome. For example, 1-phenylpyrrole with electron-donating groups (Me, *t*-Bu, OMe) gave **3ba**–**3da** in good yields of 62–83% (Table 3, entries 1–3). Meanwhile, 1-phenylpyrrole with electron-withdrawing groups (F, Cl, Br, CF_3_, OCF_3_, CN) also gave the corresponding products **3ea**–**3ja** in good to excellent yields of 71–93% (Table 3, entry 4–9). However, when R was biphenyl, the desired product **3ka** was only obtained in moderate yield of 35%, probably due to the poor solubility of the starting materials (Table 3, entry 10). In contrast, when N-substituents (R) were Ts, Boc, Bn or methyl, no desired product was detected by thin layer chromatography (TLC) experiments (Table 3, entries 11–13). Interestingly, the method of Lautens works with an *N*-Boc pyrrole [21].

**Table 3:** Scope of N-substituted pyrroles **1**.^a^

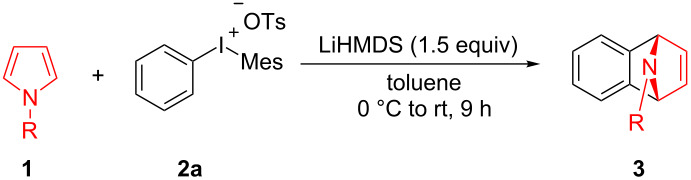			

entry	R	product	yield (%)^b^

1	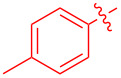 **1b**	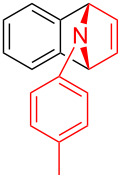 **3ba**	77
2	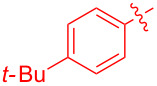 **1c**	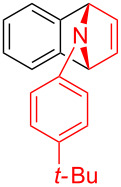 **3ca**	83
3	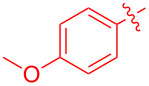 **1d**	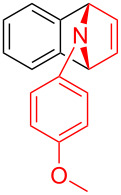 **3da**	62
4	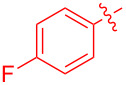 **1e**	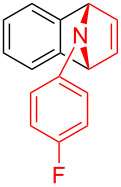 **3ea**	82
5	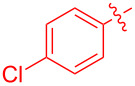 **1f**	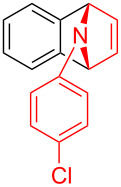 **3fa**	81
6	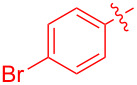 **1g**	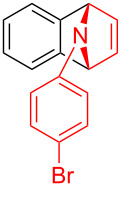 **3ga**	71
7	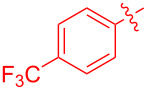 **1h**	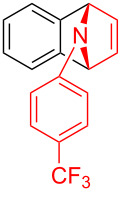 **3ha**	90
8	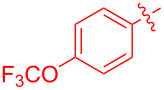 **1i**	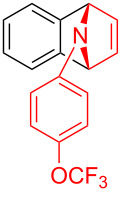 **3ia**	83
9	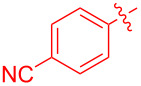 **1j**	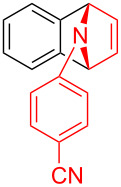 **3ja**	93
10	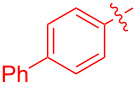 **1k**	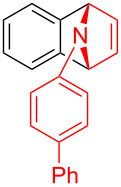 **3ka**	35
11	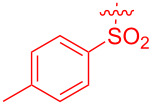 **1l**	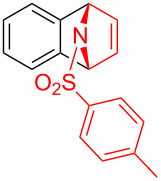 **3la**	0
12	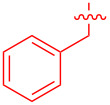 **1m**	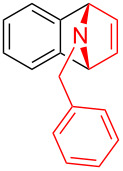 **3ma**	0
13	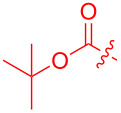 **1n**	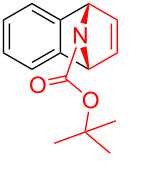 **3na**	0

^a^Reaction conditions: **1** (2.5 mmol, 5 equiv), **2a** (0.5 mmol), LiHMDS (1 M in toluene, 0.75 mL, 1.5 equiv), toluene (5 mL), 0 °C to rt, 9 h. ^b^Isolated yield. Mes = 2,4,6-trimethylphenyl. OTs = 4-toluenesulfonate.

**Scheme 2:**
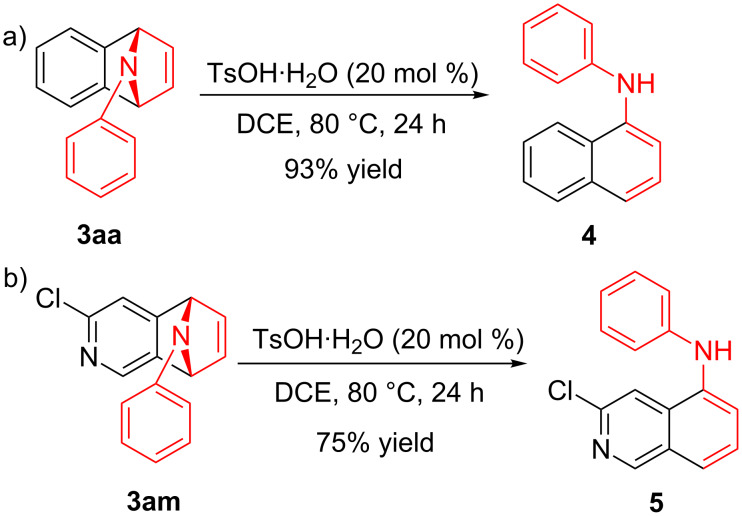
Formation of *N*-phenylamine derivatives **4** and **5** via ring opening reactions.

**Scheme 3:**
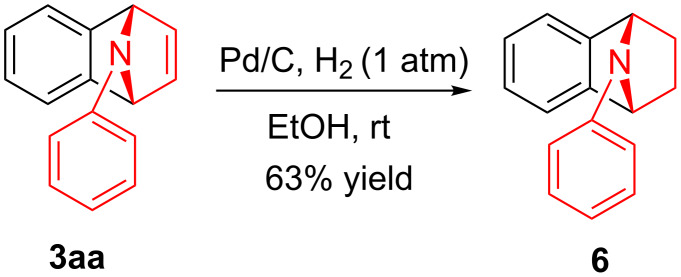
Preparation of product **6** by hydrogenation.

To demonstrate the practical utility of this methodology, treatment of **3aa** with 20 mol % TsOH·H_2_O in DCE at 80 °C resulted in *N*-phenylnaphthalen-1-ylamine (**4**) in 93% yield [23], which was widely used in dye-sensitized solar cells [24], hole transport materials [25–26] and organic light-emitting diodes (OLEDs, Scheme 2a) [27]. In another similar reaction with **3am**, a novel *N*-phenylamine derivative **5** was synthesized in 75% yield (Scheme 2b), whose structure was determined by 2D-NMR analyses (see Supporting Information File 1). Furthermore, as a unique electron donor, the novel compound **5** may have potential applications in photosensitive dyes and OLEDs [28–29]. Interestingly, the bridged-ring compound **6** could be easily obtained in 63% yield with palladium on carbon catalyst under hydrogen atmosphere at room temperature (Scheme 3).

## Conclusion

In summary, we have demonstrated a Diels–Alder cycloaddition of *N*-arylpyrroles by using diaryliodonium salts under mild conditions. The synthetic method was extended to a wide range of substrates. As such, various bridged-ring amines were prepared in moderate to excellent yields of 35–96%. Additionally, the resulting products could be easily converted to *N*-phenylamine derivatives and hydrogenated products in good yields. Further investigations on the application of this transformation are underway in our laboratory.

## Supporting Information

File 1 Experimental procedures and characterization data of all products, copies of ^1^H, ^13^C, ^19^F NMR and HRMS spectra of all compounds.
