# Supplementary material for: Diels–Alder cycloadditions of N-arylpyrroles via aryne intermediates using diaryliodonium salts
Source: Beilstein J Org Chem. 2018 Feb 6;14:354–63. doi: 10.3762/bjoc.14.23 (PMC5815300; doi:10.3762/bjoc.14.23)
Supplement: File 1 — Experimental procedures and characterization data of all products, copies of 1H, 13C, 19F NMR and HRMS spectra of all compounds. [file Beilstein_J_Org_Chem-14-354-s001.pdf]

Supporting Information

for

Diels–Alder cycloadditions of *N*-arylpyrroles via aryne intermediates using diaryliodonium salts

Huangguan Chen[1], Jianwei Han*[1,2] and Limin Wang*[1]

Address: [1]Key Laboratory for Advanced Materials, Institute of Fine Chemicals, School of Chemistry & Molecular Engineering, East China University of Science and Technology, 130 Meilong Road, Shanghai 200237, P. R. China and [2]Shanghai–Hong Kong Joint Laboratory in Chemical Synthesis, Shanghai Institute of Organic Chemistry, The Chinese Academy of Sciences, 345 Lingling Road, Shanghai 200032, China

Email: Jianwei Han* - jianweihan@ecust.edu.cn; Limin Wang* - wanglimin@ecust.edu.cn.

*Corresponding author

Experimental procedures and characterization data of all products, copies of ^{1}H, ^{13}C, ^{19}F NMR and HRMS spectra of all compounds

Table of contents

Part 1. General information……………………..……………….……..…….……S3

a. Methods

b. Materials

Part 2. Synthesis and characterization of *N*-substituted pyrroles…..….......S4

Part 3. Synthesis and characterization of products from cycloaddition reactions…………………………………………..…………………….…S8–S18

Part 4. Synthesis and characterization of products from isomerization reactions……………………………………………………………………....S19

Part 5. Synthesis and characterization of products from hydrogenation reactions……………………………………………………..……….......S20

Part 6. References……………………………………………………….......S21

Part 7. Copies of ^{1}H, ^{13}C, ^{19}F NMR and HRMS (ESI) spectra…….S22–S85

Part 1. General nformation

a. Methods:

^{1}H, ^{13}C, and ^{19}F NMR spectra were recorded in $CDCl_3$ or DMSO-d_6 (with tetramethylsilane as an internal standard) on a Bruker AVANCE 400 spectrometer, operating at 400 MHz, 100 MHz, and 376 MHz respectively. Chemical shifts (δ) are reported in ppm, and coupling constants (*J*) are in Hertz (Hz). The following abbreviations were used to explain the multiplicities: s = singlet, d = doublet, t = triplet, q = quartet, m = multiplet, br = broad; High resolution mass spectrometry (HRMS) was performed on an ESI–TOF spectrometer; reactions were monitored by TLC (detection with UV light); Column chromatography was performed with silica gel (200–300 mesh ASTM).

b. Materials:

All solvents were dried and/or distilled by standard methods. All reagents were purchased from commercial sources and used without further purification. The diaryliodonium salts were synthesized according to the literature procedures[1-3]. 1-Phenyl-1*H*-pyrrole, *tert*-butyl 1*H*-pyrrole-1-carboxylate, 1-tosyl-1*H*-pyrrole, and 1-methyl-1*H*-pyrrole were commercially available and were used as recevied. The preparation of all other materials is described in detail below.

Part 2. Synthesis and characterization of *N*-substituted pyrroles.

General procedure 1: Synthesis of *N*-aryl pyrroles.[4]

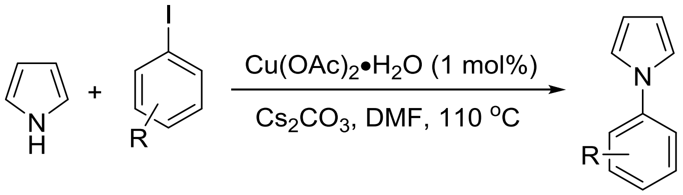

An oven-dried Schlenk tube was charged with $Cu(OAc)_2 \cdot H_2O$ (0.1 mmol, 0.01 equiv), Cs_2CO_3 (20 mmol, 2 equiv), and aryl iodide (if solid, 12 mmol, 1.2 equiv). The tube was degassed with argon for three times. Then DMF (20 mL), pyrrole (10 mmol, 1 equiv), and aryl iodide (if liquid, 12 mmol, 1.2 equiv) were added via syringe under room temperature. The mixture was stirred at 110 °C for 24 h, and then cooled down to room temperature. The reaction mixture was quenched with water (40 mL) and extracted with ethyl ether (20 mL) for three times. The combined organic layers were dried with Na_2SO_4, filtered and concentrated. The crude products were purified using flash column chromatography on silica gel to afford the desired product.

1-(*p*-Tolyl)-1*H*-pyrrole (1b)

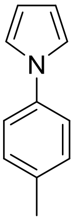

Prepared according to the **general procedure 1** on 10.0 mmol scale and obtained an isolated yield of 75% (1.18 g) as a white solid. Spectral data is consistent with that of previous reported.[5]
^{1}H NMR (400 MHz, $CDCl_3$) δ 7.34 – 7.29 (m, 2H), 7.26 (t, *J* = 9.1 Hz, 2H), 7.09 (t, *J* = 2.2 Hz, 2H), 6.37 (t, *J* = 2.2 Hz, 2H), 2.41 (s, 3H).
^{13}C NMR (100 MHz, $CDCl_3$) δ 138.5, 135.4, 130.1, 120.6, 119.4, 110.1, 20.9.

1-(4-(*tert*-Butyl)phenyl)-1*H*-pyrrole (1c)

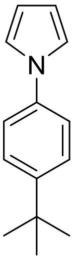

Prepared according to the **general procedure 1** on 10.0 mmol scale and obtained an isolated yield of 79% (1.58 g) as a white solid. Spectral data is consistent with that of previous reported.[6]
^{1}H NMR (400 MHz, $CDCl_3$) δ 7.48 (d, *J* = 8.6 Hz, 2H), 7.37 (d, *J* = 8.6 Hz, 2H), 7.12 (t, *J* = 2.0

Hz, 2H), 6.38 (t, J = 2.0 Hz, 2H), 1.40 (s, 9H).
^{13}C NMR (100 MHz, $CDCl_3$) δ 148.7, 138.4, 126.4, 120.3, 119.4, 110.1, 34.5, 31.4.

1-(4-Methoxyphenyl)-1*H*-pyrrole (1d)

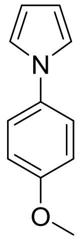

Prepared according to the **general procedure 1** on 10.0 mmol scale and obtained an isolated yield of 63% (1.09 g) as a white solid. Spectral data is consistent with that of previous reported.[5]
^{1}H NMR (400 MHz, $CDCl_3$) δ 7.27 – 7.20 (m, 2H), 6.92 (t, J = 2.2 Hz, 2H), 6.90 – 6.83 (m, 2H), 6.25 (t, J = 2.2 Hz, 2H), 3.76 (s, 3H).
^{13}C NMR (100 MHz, $CDCl_3$) δ 157.7, 134.5, 122.2, 119.7, 114.6, 109.9, 55.6.

1-(4-Fluorophenyl)-1*H*-pyrrole (1e)

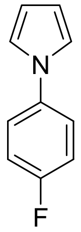

Prepared according to the **general procedure 1** on 10.0 mmol scale and obtained an isolated yield of 83% (1.33 g) as a white solid. Spectral data is consistent with that of previous reported.[7]
^{1}H NMR (400 MHz, $CDCl_3$) δ 7.46 – 7.30 (m, 2H), 7.23 – 7.09 (m, 2H), 7.05 (t, J = 2.2 Hz, 2H), 6.38 (t, J = 2.2 Hz, 2H).
^{13}C NMR (100 MHz, $CDCl_3$) δ 160.6 (d, $J_{C\text{-}F}$ = 243.4 Hz), 137.16, 122.3 (d, $J_{C\text{-}F}$ = 8.1 Hz), 119.64, 116.3 (d, $J_{C\text{-}F}$ = 22.7 Hz), 110.47.
^{19}F NMR (376 MHz, $CDCl_3$) δ -117.10 (s, 1F).

1-(4-Chlorophenyl)-1*H*-pyrrole (1f)

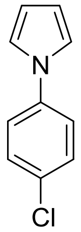

Prepared according to the **general procedure 1** on 10.0 mmol scale and obtained an isolated yield of 87% (1.54 g) as a colorless solid. Spectral data is consistent with that of previous reported.[5]
^{1}H NMR (400 MHz, $CDCl_3$) δ 7.42 – 7.34 (m, 2H), 7.34 – 7.28 (m, 2H), 7.04 (t, J = 2.2 Hz, 2H), 6.35 (t, J = 2.2 Hz, 2H).
^{13}C NMR (100 MHz, $CDCl_3$) δ 139.3, 131.0, 129.6, 121.6, 119.3, 110.8.

1-(4-Bromophenyl)-1*H*-pyrrole (1g)

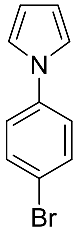

Prepared according to the **general procedure 1** on 10.0 mmol scale and obtained an isolated yield of 83% (1.85 g) as a white solid. Spectral data is consistent with that of previous reported.[8]

^{1}H NMR (400 MHz, $CDCl_3$) δ 7.58 – 7.48 (m, 2H), 7.32 – 7.20 (m, 2H), 7.04 (t, *J* = 2.2 Hz, 2H), 6.35 (t, *J* = 2.2 Hz, 2H).

^{13}C NMR (100 MHz, $CDCl_3$) δ 139.8, 132.6, 122.0, 119.2, 118.7, 110.9.

1-(4-(Trifluoromethyl)phenyl)-1*H*-pyrrole (1h)

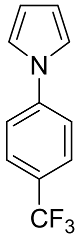

Prepared according to the **general procedure 1** on 10.0 mmol scale and obtained an isolated yield of 95% (2.00 g) as a white solid. Spectral data is consistent with that of previous reported.[9]

^{1}H NMR (400 MHz, $CDCl_3$) δ 7.72 (d, *J* = 8.4 Hz, 2H), 7.52 (d, *J* = 8.4 Hz, 2H), 7.17 (t, *J* = 2.2 Hz, 2H), 6.43 (t, *J* = 2.2 Hz, 2H).

^{13}C NMR (100 MHz, $CDCl_3$) δ 143.2, 127.4 (q, J_{C-F} = 32.7 Hz), 126.9 (q, J_{C-F} = 3.7 Hz), 124.0 (q, J_{C-F} = 270.1 Hz), 120.0, 119.1, 111.5.

^{19}F NMR (376 MHz, $CDCl_3$) δ -62.20 (s, 3F).

1-(4-(Trifluoromethoxy)phenyl)-1*H*-pyrrole (1i)

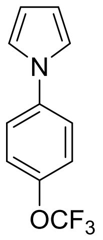

Prepared according to the **general procedure 1** on 10.0 mmol scale and obtained an isolated yield of 95% (2.15 g) as a white solid.

^{1}H NMR (400 MHz, $CDCl_3$) δ 7.46 – 7.32 (m, 2H), 7.27 (d, *J* = 8.6 Hz, 2H), 7.05 (t, *J* = 2.1 Hz, 2H), 6.36 (t, *J* = 2.1 Hz, 2H).

^{13}C NMR (100 MHz, $CDCl_3$) δ 146.7, 139.4, 122.3, 121.6, 120.5 (q, J_{C-F} = 255.7 Hz), 119.2, 110.9.

^{19}F NMR (376 MHz, $CDCl_3$) δ -58.10 (s, 3F).

4-(1*H*-Pyrrol-1-yl)benzonitrile (1j)

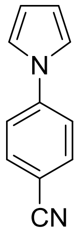

Prepared according to the **general procedure 1** on 10.0 mmol scale and obtained an isolated yield of 89% (1.50 g) as a white solid. Spectral data is consistent with that of previous reported.[7]

^{1}H NMR (400 MHz, $CDCl_3$) δ 7.79 – 7.61 (m, 2H), 7.57 – 7.37 (m, 2H), 7.13 (t, *J* = 2.2 Hz, 2H), 6.40 (t, *J* = 2.2 Hz, 2H).

^{13}C NMR (100 MHz, $CDCl_3$) δ 143.7, 133.8, 120.0, 118.9, 118.5, 112.2, 108.6.

1-([1,1'-Biphenyl]-4-yl)-1*H*-pyrrole (1k)

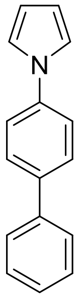

Prepared according to the **general procedure 1** on 10.0 mmol scale and obtained an isolated yield of 82% (1.79 g) as a white solid. Spectral data is consistent with that of previous reported.[5]

^{1}H NMR (400 MHz, $CDCl_3$) δ 7.73 – 7.59 (m, 4H), 7.51 (m, 4H), 7.46 – 7.36 (m, 1H), 7.19 (t, *J* = 2.2 Hz, 2H), 6.44 (t, *J* = 2.2 Hz, 2H).

^{13}C NMR (100 MHz, $CDCl_3$) δ 140.2, 140.0, 138.6, 128.9, 128.2, 127.4, 127.0, 120.7, 119.3, 110.6.

Part 3. Synthesis and characterization of products from cycloaddition reactions.

General procedure 2: Synthesis of Diels–Alder reaction products.

To an oven-dried Schlenk tube was added iodonium salts (0.5 mmol, 1 equiv) and substituted pyrrole (if solid, 2.5 mmol, 5 equiv). The tube was degassed with argon for three times. Then the tube was placed in an ice bath. Toluene (4.25 mL) and substituted pyrrole (if liquid, 2.5 mmol, 5 equiv) were added sequentially via syringe. After being stirred for approximately 10 minutes, LiHMDS (0.75 mL (1 M in toluene), 0.75 mmol, 1.5 equiv) was added via syringe and the mixture was allowed to warm up to room temperature gradually. After TLC indicated that the iodonium salts were completely consumed, the reaction mixture was quenched by addition of an aqueous solution of ammonium chloride (5 mL) and extracted with DCM (10 mL) for three times. The combined organic layers were dried with Na_2SO_4, filtered and concentrated. The crude products were purified using flash column chromatography on silica gel to afford the desired product.

9-Phenyl-1,4-dihydro-1,4-epiminonaphthalene (3aa)

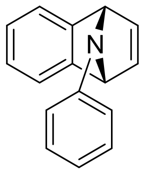

Prepared according to the **general procedure 2** on 0.5 mmol scale and obtained an isolated yield of 85% (93 mg) as a pale brown solid. Spectral data is consistent with that of previous reported.

^{1}H NMR (400 MHz, $CDCl_3$) δ 7.26 (m, 2H), 7.17 (m, 2H), 6.97 – 6.88 (m, 4H), 6.87 – 6.78 (m, 3H), 5.43 (t, *J* = 1.4 Hz, 2H).

^{13}C NMR (100 MHz, $CDCl_3$) δ 148.6, 146.9, 141.9, 128.8, 124.9, 121.5, 120.8, 118.0, 69.3.

HRMS (ESI) calculated for $C_{16}H_{14}N$ $[M+H]^+$ 220.1126, found 220.1127.

6-Methyl-9-phenyl-1,4-dihydro-1,4-epiminonaphthalene (3ab)

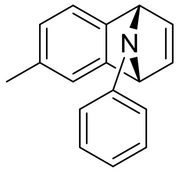

Prepared according to the **general procedure 2** on 0.5 mmol scale and obtained an isolated yield of 63% (74 mg) as a pale brown oil.

^{1}H NMR (400 MHz, $CDCl_3$) δ 7.08 (ddd, *J* = 13.6, 6.2, 4.8 Hz, 1H), 7.01 (s, 1H), 6.90 – 6.80 (m, 2H), 6.79 – 6.68 (m, 3H), 6.64 (dd, *J* = 7.2, 0.5 Hz, 1H), 5.36 – 5.24 (m, 2H), 2.16 (s, 3H).

^{13}C NMR (100 MHz, $CDCl_3$) δ 148.8, 147.0, 145.6, 142.2, 141.7, 134.6, 128.8, 125.0, 122.9, 121.2, 120.8, 118.0, 69.3, 69.0, 21.3.

HRMS (ESI) calculated for $C_{17}H_{16}N$ $[M+H]^+$ 234.1283, found 234.1276.

6-(*tert*-Butyl)-9-phenyl-1,4-dihydro-1,4-epiminonaphthalene (3ac)

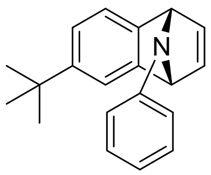

Prepared according to the **general procedure 2** on 0.5 mmol scale and obtained an isolated yield of 57% (78 mg) as a pale yellow oil.

^{1}H NMR (400 MHz, $CDCl_3$) δ 7.27 (d, *J* = 1.5 Hz, 1H), 7.10 (m, 3H), 6.86 (dd, *J* = 7.5, 1.7 Hz, 1H), 6.80 (m, 2H), 6.78 – 6.73 (m, 3H), 5.33 (m, 2H), 1.19 (s, 9H).

^{13}C NMR (100 MHz, $CDCl_3$) δ 148.5, 148.1, 147.0, 145.4, 141.6, 141.4, 128.8, 121.2, 120.8, 120.6, 119.1, 118.1, 69.6, 69.2, 34.7, 31.6.

HRMS (ESI) calculated for $C_{20}H_{22}N$ $[M+H]^+$ 276.1752, found 276.1749.

6-Fluoro-9-phenyl-1,4-dihydro-1,4-epiminonaphthalene (3ad)

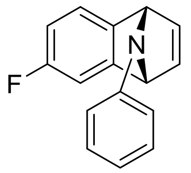

Prepared according to the **general procedure 2** on 0.5 mmol scale and obtained an isolated yield of 77% (92 mg) as a pale yellow oil.

^{1}H NMR (400 MHz, $CDCl_3$) δ 7.13 – 7.03 (m, 3H), 6.92 (dd, *J* = 7.8, 2.3 Hz, 1H), 6.90 – 6.81 (m, 2H), 6.80 – 6.68 (m, 3H), 6.50 (ddd, *J* = 10.0, 7.8, 2.3 Hz, 1H), 5.38 – 5.24 (m, 2H).

^{13}C NMR (100 MHz, $CDCl_3$) δ 160.5 (d, $J_{C\text{-}F}$ = 242.9 Hz), 151.4 (d, $J_{C\text{-}F}$ = 9.0 Hz), 146.63, 143.8 (d, $J_{C\text{-}F}$ = 3.0 Hz), 142.45, 141.44, 128.91, 121.9 (d, $J_{C\text{-}F}$ = 9.0 Hz), 121.07, 117.95, 110.7 (d, $J_{C\text{-}F}$ = 25.0 Hz), 110.4 (d, $J_{C\text{-}F}$ = 22.0 Hz), 69.3 (d, $J_{C\text{-}F}$ = 3.0 Hz), 68.78.

^{19}F NMR (376 MHz, $CDCl_3$) δ -117.76 (s, 1F).

HRMS (ESI) calculated for $C_{16}H_{13}NF$ $[M+H]^+$ 238.1032, found 238.1038.

6-Chloro-9-phenyl-1,4-dihydro-1,4-epiminonaphthalene (3ae)

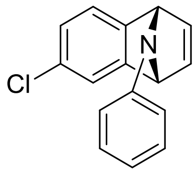

Prepared according to the **general procedure 2** on 0.5 mmol scale and obtained an isolated yield of 87% (110 mg) as a pale brown oil.

^{1}H NMR (400 MHz, $CDCl_3$) δ 7.15 (m, 1H), 7.12 – 7.04 (m, 3H), 6.88 – 6.79 (m, 3H), 6.79 – 6.74 (m, 1H), 6.72 (m, 2H), 5.31 (m, 2H).

^{13}C NMR (100 MHz, $CDCl_3$) δ 150.9, 147.1, 146.5, 142.2, 141.6, 130.6, 128.9, 124.6, 122.5, 122.3, 121.1, 117.9, 69.1, 68.8.

HRMS (ESI) calculated for $C_{16}H_{13}NCl$ $[M+H]^+$ 254.0737, found 254.0730.

6-Bromo-9-phenyl-1,4-dihydro-1,4-epiminonaphthalene (3af)

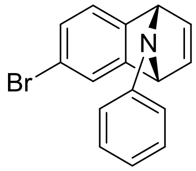

Prepared according to the **general procedure 2** on 0.5 mmol scale and obtained an isolated yield of 96% (143 mg) as a yellow oil.

^{1}H NMR (400 MHz, $CDCl_3$) δ 7.30 (d, *J* = 1.6 Hz, 1H), 7.13 – 7.06 (m, 2H), 7.03 (d, *J* = 7.5 Hz, 1H), 6.98 (dd, *J* = 7.5, 1.7 Hz, 1H), 6.90 – 6.81 (m, 2H), 6.80 – 6.74 (m, 1H), 6.74 – 6.69 (m, 2H), 5.35 – 5.24 (m, 2H).

^{13}C NMR (100 MHz, $CDCl_3$) δ 151.2, 147.7, 146.4, 142.2, 141.7, 128.9, 127.6, 125.2, 122.8, 121.1, 118.6, 117.9, 69.1, 68.9.

HRMS (ESI) calculated for $C_{16}H_{13}NBr$ $[M+H]^+$ 298.0231, found 298.0231.

9-Phenyl-1,4-dihydro-1,4-epiminonaphthalene-6-carbonitrile (3ag)

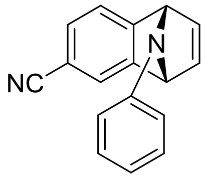

Prepared according to the **general procedure 2** on 0.5 mmol scale and obtained an isolated yield of 71% (87 mg) as a white solid.

m.p.: 187-188 °C.

^{1}H NMR (400 MHz, $CDCl_3$) δ 7.39 (s, 1H), 7.26 (m, 1H), 7.22 (m, 1H), 7.15 – 7.06 (m, 2H), 6.94 – 6.85 (m, 2H), 6.79 (t, *J* = 7.4 Hz, 1H), 6.72 (dd, *J* = 8.6, 0.9 Hz, 2H), 5.54 – 5.10 (m, 2H).

^{13}C NMR (100 MHz, $CDCl_3$) δ 154.2, 150.0, 145.9, 142.1, 141.5, 130.8, 129.0, 124.1, 122.0, 121.5, 119.3, 117.8, 108.6, 69.2, 68.8.

HRMS (ESI) calculated for $C_{17}H_{13}N_2$ $[M+H]^+$ 245.1079, found 245.1070.

6-Nitro-9-phenyl-1,4-dihydro-1,4-epiminonaphthalene (3ah)

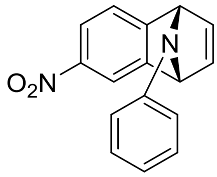

Prepared according to the **general procedure 2** on 0.5 mmol scale and obtained an isolated yield of 70% (92 mg) as a yellow oil.

^{1}H NMR (400 MHz, $CDCl_3$) δ 7.97 (d, *J* = 1.9 Hz, 1H), 7.83 (m, 1H), 7.27 (d, *J* = 7.8 Hz, 1H), 7.17 – 7.04 (m, 2H), 6.93 (d, *J* = 2.4 Hz, 1H), 6.89 (d, *J* = 2.4 Hz, 1H), 6.78 (t, *J* = 7.4 Hz, 1H), 6.72 (m, 2H), 5.43 (m, 2H).

^{13}C NMR (100 MHz, $CDCl_3$) δ 156.2, 150.8, 145.9, 145.7, 142.5, 141.4, 129.1, 122.3, 121.5, 121.4, 117.8, 116.5, 69.0, 69.0.

HRMS (ESI) calculated for $C_{16}H_{11}N_2O_2$ $[M-H]^+$ 263.0821, found 263.0825.

9-Phenyl-6-(trifluoromethyl)-1,4-dihydro-1,4-epiminonaphthalene (3ai)

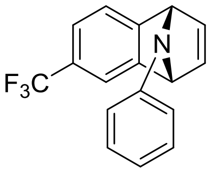

Prepared according to the **general procedure 2** on 0.5 mmol scale and obtained an isolated yield of 77% (110 mg) as a pale yellow oil.

^{1}H NMR (400 MHz, $CDCl_3$) δ 7.39 (s, 1H), 7.24 (d, J = 7.4 Hz, 1H), 7.18 – 7.13 (m, 1H), 7.10 (t, J = 7.9 Hz, 2H), 6.90 – 6.81 (m, 2H), 6.81 – 6.69 (m, 3H), 5.39 (m, 2H).

^{13}C NMR (100 MHz, $CDCl_3$) δ 152.8, 149.8, 146.3, 142.0, 141.6, 129.0, 127.3 (q, $J_{C\text{-}F}$ = 31.7 Hz), 124.3 (q, $J_{C\text{-}F}$ = 270.5 Hz), 122.9 (q, $J_{C\text{-}F}$ = 4.0 Hz), 121.3, 121.2, 118.1 (q, $J_{C\text{-}F}$ = 4.0 Hz), 117.9, 69.1.

^{19}F NMR (376 MHz, $CDCl_3$) δ -61.84 (s, 3F).

HRMS (ESI) calculated for $C_{17}H_{13}NF_3$ $[M+H]^+$ 288.1000, found 288.1003.

9-Phenyl-6-(trifluoromethoxy)-1,4-dihydro-1,4-epiminonaphthalene (3aj)

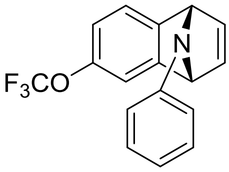

Prepared according to the **general procedure 2** on 0.5 mmol scale and obtained an isolated yield of 88% (134 mg) as a yellow oil.

^{1}H NMR (400 MHz, $CDCl_3$) δ 7.14 – 7.07 (m, 3H), 7.05 (s, 1H), 6.87 – 6.80 (m, 2H), 6.80 – 6.75 (m, 1H), 6.75 – 6.66 (m, 3H), 5.34 (m, 2H).

^{13}C NMR (100 MHz, $CDCl_3$) δ 151.2, 147.2, 146.5, 146.4, 142.0, 141.6, 129.0, 121.7, 121.2, 120.5 (q, $J_{C\text{-}F}$ = 255.1 Hz), 118.0, 117.1, 115.6, 69.3, 69.0.

^{19}F NMR (376 MHz, $CDCl_3$) δ -57.88 (s, 3F).

HRMS (ESI) calculated for $C_{17}H_{13}NOF_3$ $[M+H]^+$ 304.0949, found 304.0944.

6,9-Diphenyl-1,4-dihydro-1,4-epiminonaphthalene (3ak)

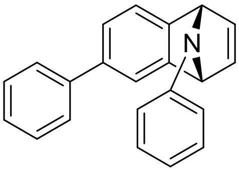

Prepared according to the **general procedure 2** on 0.5 mmol scale and obtained an isolated yield of 89% (131 mg) as a yellow oil.

^{1}H NMR (400 MHz, $CDCl_3$) δ 7.42 (m, 3H), 7.30 (m, 2H), 7.20 (m, 2H), 7.14 – 7.01 (m, 3H), 6.91 – 6.81 (m, 2H), 6.75 (m, 3H), 5.42 – 5.31 (m, 2H).

^{13}C NMR (100 MHz, $CDCl_3$) δ 149.5, 147.6, 146.9, 141.9, 141.8, 141.4, 138.4, 128.9, 128.7, 127.2, 127.1, 124.0, 121.6, 121.0, 120.8, 118.1, 69.4, 69.2.

HRMS (ESI) calculated for $C_{22}H_{18}N$ $[M+H]^+$ 296.1439, found 296.1432.

5,9-Diphenyl-1,4-dihydro-1,4-epiminonaphthalene (3al)

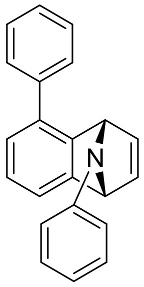

Prepared according to the **general procedure 2** on 0.5 mmol scale and obtained an isolated yield of 82% (121 mg) as a yellow oil.

^{1}H NMR (400 MHz, $CDCl_3$) δ 7.44 – 7.35 (m, 2H), 7.35 – 7.27 (m, 3H), 7.17 (dd, *J* = 5.4, 2.6 Hz, 1H), 7.08 – 7.00 (m, 2H), 6.96 (dd, *J* = 5.4, 2.2 Hz, 1H), 6.95 – 6.88 (m, 3H), 6.76 – 6.69 (m, 1H), 6.66 (dd, *J* = 8.6, 1.0 Hz, 2H), 5.46 (m, 1H), 5.42 – 5.37 (m, 1H).

^{13}C NMR (100 MHz, $CDCl_3$) δ 148.7, 146.8, 146.4, 142.2, 141.6, 140.0, 135.9, 128.8, 128.8, 128.3, 127.4, 125.2, 120.9, 120.5, 118.0, 69.6, 68.4.

HRMS (ESI) calculated for $C_{22}H_{18}N$ $[M+H]^+$ 296.1439, found 296.1436.

3-Chloro-9-phenyl-5,8-dihydro-5,8-epiminoisoquinoline (3am)

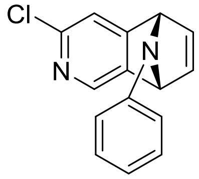

Prepared according to the **general procedure 2** on 0.5 mmol scale and obtained an isolated yield of 80% (102 mg) as a yellow oil.

^{1}H NMR (400 MHz, $CDCl_3$) δ 8.07 (s, 1H), 7.14 (s, 1H), 7.14 – 7.08 (m, 2H), 6.91 (m, 1H), 6.85 – 6.76 (m, 2H), 6.70 (m, 2H), 5.40 (m, 1H), 5.34 (m, 1H).

^{13}C NMR (100 MHz, $CDCl_3$) δ 162.1, 148.4, 145.6, 143.2, 142.7, 140.5, 140.1, 129.1, 121.7, 118.6, 117.7, 68.6, 66.7.

HRMS (ESI) calculated for $C_{15}H_{10}N_2Cl$ $[M-H]^+$ 253.0533, found 253.0536.

9-Phenyl-1,4-dihydro-1,4-epiminonaphthalene-5-carbonitrile (3an)

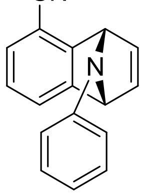

Prepared according to the **general procedure 2** on 0.5 mmol scale and obtained an isolated yield of 71% (86 mg) as a pale yellow oil.

^{1}H NMR (400 MHz, $CDCl_3$) δ 7.33 (d, *J* = 7.1 Hz, 1H), 7.15 – 7.07 (m, 2H), 7.06 – 7.01 (m, 1H), 6.96 – 6.86 (m, 3H), 6.83 – 6.75 (m, 1H), 6.75 – 6.70 (m, 2H), 5.60 (m, 1H), 5.41 (m, 1H).

^{13}C NMR (100 MHz, $CDCl_3$) δ 154.3, 150.3, 145.8, 142.8, 141.2, 129.1, 127.0, 126.0, 125.2, 121.5,

117.8, 117.2, 106.3, 69.5, 68.3.

HRMS (ESI) calculated for $C_{17}H_{13}N_2$ $[M+H]^+$ 245.1079, found 245.1070.

5-Fluoro-9-phenyl-1,4-dihydro-1,4-epiminonaphthalene (3ao)

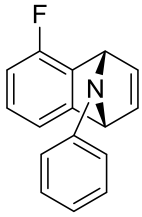

Prepared according to the **general procedure 2** on 0.5 mmol scale and obtained an isolated yield of 78% (92 mg) as a yellow oil.

^{1}H NMR (400 MHz, $CDCl_3$) δ 7.12 – 7.03 (m, 2H), 6.95 (d, *J* = 7.0 Hz, 1H), 6.90 – 6.86 (m, 2H), 6.82 – 6.76 (m, 1H), 6.76 – 6.70 (m, 3H), 6.54 (td, *J* = 8.4, 0.6 Hz, 1H), 5.60 (m, 1H), 5.35 (m, 1H).

^{13}C NMR (100 MHz, $CDCl_3$) δ 157.4 (d, J_{C-F} = 244.3 Hz), 152.3 (d, J_{C-F} = 4.6 Hz), 146.5, 142.3, 141.6, 133.0 (d, J_{C-F} = 20.5 Hz), 129.0, 127.3 (d, J_{C-F} = 6.0 Hz), 121.2, 117.9, 117.7 (d, J_{C-F} = 2.6 Hz), 113.6 (d, J_{C-F} = 22.1 Hz), 69.6, 65.7.

^{19}F NMR (376 MHz, $CDCl_3$) δ -121.42 (s, 1F).

HRMS (ESI) calculated for $C_{16}H_{13}NF$ $[M+H]^+$ 238.1032, found 238.1026.

5-Methyl-9-phenyl-1,4-dihydro-1,4-epiminonaphthalene (3ap)

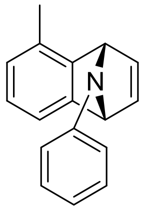

Prepared according to the **general procedure 2** on 0.5 mmol scale and obtained an isolated yield of 60% (70 mg) as a yellow oil.

^{1}H NMR (400 MHz, $CDCl_3$) δ 7.08 (m, 2H), 7.00 (d, *J* = 6.8 Hz, 1H), 6.84 (s, 2H), 6.74 (m, 4H), 6.65 (d, *J* = 7.7 Hz, 1H), 5.44 (m, 1H), 5.33 (m, 1H), 2.26 (s, 3H).

^{13}C NMR (100 MHz, $CDCl_3$) δ 148.2, 147.1, 146.9, 142.0, 141.4, 130.8, 128.8, 126.6, 124.9, 120.8, 119.0, 118.0, 69.7, 67.4, 18.2.

HRMS (ESI) calculated for $C_{17}H_{16}N$ $[M+H]^+$ 234.1283, found 234.1281.

5,7-Difluoro-9-phenyl-1,4-dihydro-1,4-epiminonaphthalene (3aq)

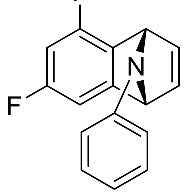

Prepared according to the **general procedure 2** on 0.5 mmol scale and obtained an isolated yield of 48% (62 mg) as a yellow oil.

^{1}H NMR (400 MHz, $CDCl_3$) δ 7.13 – 7.06 (m, 2H), 6.90 (qdd, J = 5.5, 2.4, 0.4 Hz, 2H), 6.80 – 6.74 (m, 2H), 6.73 – 6.69 (m, 2H), 6.29 (ddd, J = 9.6, 8.2, 1.9 Hz, 1H), 5.62 – 5.49 (m, 1H), 5.38 – 5.25 (m, 1H).
^{13}C NMR (100 MHz, $CDCl_3$) δ 161.0 (dd, $J_{C\text{-}F}$ = 246.9, 9.2 Hz), 156.4 (dd, $J_{C\text{-}F}$ = 246.2, 12.7 Hz), 153.9 (dd, $J_{C\text{-}F}$ = 9.4, 6.0 Hz), 146.2, 142.1, 141.8, 129.0, 128.6 (dd, $J_{C\text{-}F}$ = 21.1, 3.2 Hz), 121.4, 117.8, 107.3 (dd, $J_{C\text{-}F}$ = 25.0, 4.0 Hz), 100.6 (t, $J_{C\text{-}F}$ = 26.2 Hz), 69.7, 65.4 (d, $J_{C\text{-}F}$ = 1.4 Hz).
^{19}F NMR (376 MHz, $CDCl_3$) δ -113.42 (d, J = 5.6 Hz, 1F), -117.91 (d, J = 5.6 Hz, 1F).
HRMS (ESI) calculated for $C_{16}H_{12}NF_2$ $[M+H]^+$ 256.0938, found 256.0947.

5,6-Dimethyl-9-phenyl-1,4-dihydro-1,4-epiminonaphthalene (3ar)

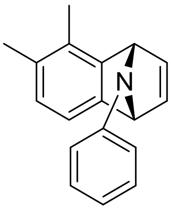

Prepared according to the **general procedure 2** on 0.5 mmol scale and obtained an isolated yield of 62% (76 mg) as a yellow oil.
^{1}H NMR (400 MHz, $CDCl_3$) δ 7.16 (m, 2H), 6.99 (d, J = 7.1 Hz, 1H), 6.95 – 6.87 (m, 2H), 6.85 – 6.76 (m, 3H), 6.69 (d, J = 7.1 Hz, 1H), 5.52 (m, 1H), 5.37 (m, 1H), 2.25 (s, 3H), 2.15 (s, 3H).
^{13}C NMR (100 MHz, $CDCl_3$) δ 147.2, 145.6, 142.3, 141.3, 133.6, 130.2, 128.8, 125.6, 120.7, 118.8, 118.0, 69.6, 67.7, 19.6, 15.6.
HRMS (ESI) calculated for $C_{18}H_{18}N$ $[M+H]^+$ 248.1439, found 248.1448.

5,7-Dimethyl-9-phenyl-1,4-dihydro-1,4-epiminonaphthalene (3as)

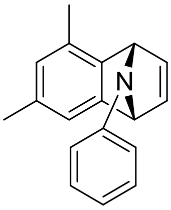

Prepared according to the **general procedure 2** on 0.5 mmol scale and obtained an isolated yield of 58% (72 mg) as a yellow oil.
^{1}H NMR (400 MHz, $CDCl_3$) δ 7.20 – 7.12 (m, 2H), 6.94 – 6.89 (m, 3H), 6.84 – 6.77 (m, 3H), 6.54 (s, 1H), 5.47 (m, 1H), 5.36 (m, 1H), 2.29 (s, 3H), 2.20 (s, 3H).
^{13}C NMR (100 MHz, $CDCl_3$) δ 148.5, 147.2, 143.9, 141.8, 141.7, 134.5, 130.5, 128.8, 126.8, 120.7, 120.4, 118.0, 69.6, 67.1, 21.2, 18.1.
HRMS (ESI) calculated for $C_{18}H_{18}N$ $[M+H]^+$ 248.1439, found 248.1442.

9-(*p*-Tolyl)-1,4-dihydro-1,4-epiminonaphthalene (3ba)

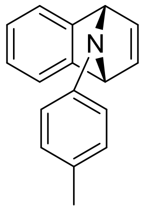

Prepared according to the **general procedure 2** on 0.5 mmol scale and obtained an isolated yield of 81% (94 mg) as a pale brown solid.

m.p.: 123-125 °C.

^{1}H NMR (400 MHz, $CDCl_3$) δ 7.34 – 7.25 (m, 2H), 7.02 (d, *J* = 8.2 Hz, 2H), 6.99 – 6.93 (m, 4H), 6.78 (d, *J* = 8.4 Hz, 2H), 5.43 (t, *J* = 1.3 Hz, 2H), 2.25 (s, 3H).

^{13}C NMR (100 MHz, $CDCl_3$) δ 148.6, 144.6, 141.8, 130.1, 129.4, 124.9, 121.5, 118.1, 69.5, 20.6.

HRMS (ESI) calculated for $C_{17}H_{16}N$ $[M+H]^+$ 234.1283, found 234.1281.

9-(4-(*tert*-Butyl)phenyl)-1,4-dihydro-1,4-epiminonaphthalene (3ca)

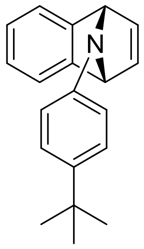

Prepared according to the **general procedure 2** on 0.5 mmol scale and obtained an isolated yield of 83% (114 mg) as a white solid.

m.p.: 107-109 °C.

^{1}H NMR (400 MHz, $CDCl_3$) δ 7.33 – 7.28 (m, 2H), 7.24 – 7.22 (m, 1H), 7.22 – 7.19 (m, 1H), 6.97 (dd, *J* = 5.1, 3.0 Hz, 2H), 6.95 (t, *J* = 1.4 Hz, 2H), 6.82 – 6.80 (m, 1H), 6.79 – 6.77 (m, 1H), 5.44 (t, *J* = 1.3 Hz, 2H), 1.28 (s, 9H).

^{13}C NMR (100 MHz, $CDCl_3$) δ 148.8, 144.3, 143.4, 141.8, 125.6, 124.8, 121.4, 117.7, 69.5, 34.0, 31.4.

HRMS (ESI) calculated for $C_{20}H_{22}N$ $[M+H]^+$ 276.1752, found 276.1745.

9-(4-Methoxyphenyl)-1,4-dihydro-1,4-epiminonaphthalene (3da)

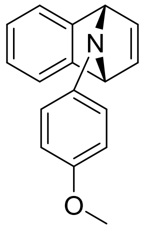

Prepared according to the **general procedure 2** on 0.5 mmol scale and obtained an isolated yield of 62% (77 mg) as a yellow oil.

^{1}H NMR (400 MHz, $CDCl_3$) δ 7.16 (dd, *J* = 5.1, 3.0 Hz, 2H), 6.86 – 6.80 (m, 4H), 6.70 – 6.62 (m, 4H), 5.24 (t, *J* = 1.4 Hz, 2H), 3.61 (s, 3H).

^{13}C NMR (100 MHz, $CDCl_3$) δ 154.1, 148.6, 141.7, 140.7, 124.9, 121.5, 119.4, 114.2, 69.9, 55.4.

HRMS (ESI) calculated for $C_{17}H_{16}NO$ $[M+H]^+$ 250.1232, found 250.1233.

9-(4-Fluorophenyl)-1,4-dihydro-1,4-epiminonaphthalene (3ea)

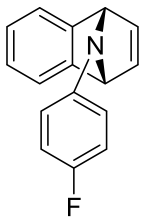

Prepared according to the **general procedure 2** on 0.5 mmol scale and obtained an isolated yield of 82% (97 mg) as a yellow oil.

^{1}H NMR (400 MHz, $CDCl_3$) δ 7.20 – 7.10 (m, 2H), 6.87 – 6.82 (m, 4H), 6.78 (t, J = 8.7 Hz, 2H), 6.73 – 6.61 (m, 2H), 5.26 (t, J = 1.4 Hz, 2H).

^{13}C NMR (100 MHz, $CDCl_3$) δ 157.7 (d, $J_{C\text{-}F}$ = 237.7 Hz), 148.3, 143.3 (d, $J_{C\text{-}F}$ = 2.4 Hz), 141.8, 125.0, 121.6, 119.2 (d, $J_{C\text{-}F}$ = 7.6 Hz), 115.4 (d, $J_{C\text{-}F}$ = 22.1 Hz), 69.8.

^{19}F NMR (376 MHz, $CDCl_3$) δ -123.68 (s, 1F).

HRMS (ESI) calculated for $C_{16}H_{13}NF$ $[M+H]^+$ 238.1032, found 238.1042.

9-(4-Chlorophenyl)-1,4-dihydro-1,4-epiminonaphthalene (3fa)

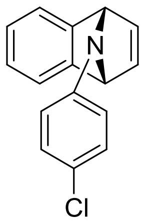

Prepared according to the **general procedure 2** on 0.5 mmol scale and obtained an isolated yield of 81% (103 mg) as a yellow solid.

m.p.: 139-141 ^{o}C.

^{1}H NMR (400 MHz, $CDCl_3$) δ 7.21 – 7.14 (m, 2H), 7.06 – 7.04 (m, 1H), 7.03 – 7.01 (m, 1H), 6.90 – 6.81 (m, 4H), 6.70 – 6.66 (m, 1H), 6.66 – 6.64 (m, 1H), 5.29 (t, J = 1.4 Hz, 2H).

^{13}C NMR (100 MHz, $CDCl_3$) δ 148.2, 145.6, 141.9, 128.8, 125.9, 125.1, 121.6, 119.2, 69.4.

HRMS (ESI) calculated for $C_{16}H_{13}NCl$ $[M+H]^+$ 254.0737, found 254.0739.

9-(4-Bromophenyl)-1,4-dihydro-1,4-epiminonaphthalene (3ga)

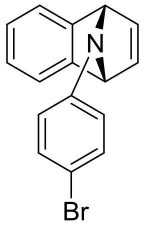

Prepared according to the **general procedure 2** on 0.5 mmol scale and obtained an isolated yield of 71% (106 mg) as a yellow solid.

m.p.: 147-148 ^{o}C.

^{1}H NMR (400 MHz, $CDCl_3$) δ 7.35 – 7.06 (m, 4H), 6.98 – 6.77 (m, 4H), 6.64 – 6.61 (m, 1H), 6.61 – 6.59 (m, 1H), 5.29 (t, J = 1.4 Hz, 2H).
^{13}C NMR (100 MHz, $CDCl_3$) δ 148.2, 146.1, 141.9, 131.7, 125.1, 121.6, 119.6, 113.3, 69.3.
HRMS (ESI) calculated for $C_{16}H_{13}NBr$ $[M+H]^+$ 298.0231, found 298.0223.

9-(4-(Trifluoromethyl)phenyl)-1,4-dihydro-1,4-epiminonaphthalene (3ha)

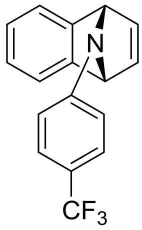

Prepared according to the **general procedure 2** on 0.5 mmol scale and obtained an isolated yield of 90% (129 mg) as a pale yellow solid.
m.p.: 97-99 ^{o}C.
^{1}H NMR (400 MHz, $CDCl_3$) δ 7.33 (d, J = 8.5 Hz, 2H), 7.20 (dd, J = 5.1, 3.0 Hz, 2H), 6.93 – 6.84 (m, 4H), 6.79 (d, J = 8.4 Hz, 2H), 5.40 (t, J = 1.4 Hz, 2H).
^{13}C NMR (100 MHz, $CDCl_3$) δ 149.7, 148.0, 142.0, 126.2 (q, J_{C-F} = 3.7 Hz), 125.2, 124.5 (q, J_{C-F} = 269.4 Hz), 122.4 (q, J_{C-F} = 32.4 Hz), 121.6, 117.2, 68.9.
^{19}F NMR (376 MHz, $CDCl_3$) δ -61.57 (s, 3F).
HRMS (ESI) calculated for $C_{17}H_{13}NF_3$ $[M+H]^+$ 288.1000, found 288.0992.

9-(4-(Trifluoromethoxy)phenyl)-1,4-dihydro-1,4-epiminonaphthalene (3ia)

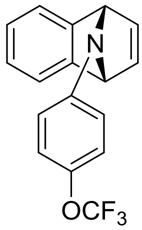

Prepared according to the **general procedure 2** on 0.5 mmol scale and obtained an isolated yield of 83% (126 mg) as a pale yellow solid.
m.p.: 74-76 ^{o}C.
^{1}H NMR (400 MHz, $CDCl_3$) δ 7.29 – 7.21 (m, 2H), 7.01 (d, J = 8.4 Hz, 2H), 6.98 – 6.90 (m, 4H), 6.81 – 6.79 (m, 1H), 6.79 – 6.75 (m, 1H), 5.38 (t, J = 1.3 Hz, 2H).
^{13}C NMR (100 MHz, $CDCl_3$) δ 148.20, 145.71, 143.02, 141.86, 125.09, 121.83, 121.67, 121.56, 119.28, 118.69, 69.45.
^{13}C NMR (100 MHz, $CDCl_3$) δ 148.2, 145.7, 143.0, 141.9, 125.1, 121.7, 121.6, 120.6 (q, J_{C-F} = 254.4 Hz), 118.7, 69.4.
^{19}F NMR (376 MHz, $CDCl_3$) δ -58.16 (s, 3F).
HRMS (ESI) calculated for $C_{17}H_{13}NOF_3$ $[M+H]^+$ 304.0949, found 304.0950.

4-(1,4-Dihydro-1,4-epiminonaphthalen-9-yl)benzonitrile (3ja)

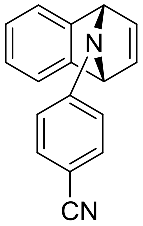

Prepared according to the **general procedure 2** on 0.5 mmol scale and obtained an isolated yield of 93% (114 mg) as a white solid.

m.p.: 191-193 °C.

^{1}H NMR (400 MHz, $CDCl_3$) δ 7.47 – 7.42 (m, 1H), 7.42 – 7.39 (m, 1H), 7.31 – 7.22 (m, 2H), 6.98 (t, J = 1.5 Hz, 2H), 6.94 (dd, J = 5.1, 3.0 Hz, 2H), 6.86 – 6.82 (m, 1H), 6.82 – 6.79 (m, 1H), 5.49 (t, J = 1.4 Hz, 2H).

^{13}C NMR (100 MHz, $CDCl_3$) δ 150.4, 147.8, 142.1, 133.2, 125.3, 121.6, 119.6, 117.3, 103.0, 68.5.

HRMS (ESI) calculated for $C_{17}H_{13}N_2$ $[M+H]^+$ 245.1079, found 245.1080.

9-([1,1'-Biphenyl]-4-yl)-1,4-dihydro-1,4-epiminonaphthalene (3ka)

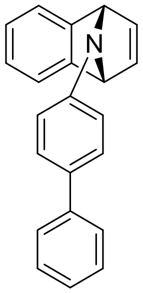

Prepared according to the **general procedure 2** on 0.5 mmol scale and obtained an isolated yield of 40% (59 mg) as a pale brown solid.

m.p.: 146-148 °C.

^{1}H NMR (400 MHz, $CDCl_3$) δ 7.53 – 7.46 (m, 2H), 7.41 (dd, J = 8.6, 1.9 Hz, 2H), 7.35 (m, 2H), 7.29 – 7.25 (m, 2H), 7.25 – 7.21 (m, 1H), 6.98 – 6.91 (m, 4H), 6.88 (dd, J = 8.6, 1.6 Hz, 2H), 5.45 (m, 2H).

^{13}C NMR (100 MHz, $CDCl_3$) δ 148.6, 146.3, 142.0, 140.8, 133.6, 128.7, 127.5, 126.6, 125.0, 121.5, 118.3, 69.3.

HRMS (ESI) calculated for $C_{22}H_{18}N$ $[M+H]^+$ 296.1439, found 296.1440.

Part 4. Synthesis and characterization of products from isomerization reaction.

General procedure 3: Synthesis of *N*-phenylamine derivatives.

To an oven-dried Schlenk tube was added 9-phenyl-1,4-dihydro-1,4-epiminonaphthalene (0.5 mmol, 1 equiv) and TsOH·H_2O (0.1 mmol, 0.2 equiv). The tube was degassed with argon for three times. Then DCE (4 mL) was added via syringe under room temperature. The mixture was stirred at 80 °C until TLC indicated that the starting materials were completely consumed. Then the reaction mixture was cooled down to room temperature and it was quenched with water (20 mL) and extracted with ethyl ether (10 mL) for three times. The combined organic layers were dried with Na_2SO_4, filtered and concentrated. The crude products were purified using flash column chromatography on silica gel to afford the desired product.

***N*-Phenylnaphthalen-1-amine (4)**

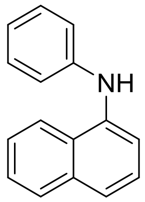

Prepared according to the **general procedure 3** on 0.5 mmol scale and obtained an isolated yield of 93% (102 mg) as a light gray solid. Spectral data is consistent with that of previous reported.[10]

^{1}H NMR (400 MHz, DMSO) δ 8.30 – 8.08 (m, 2H), 7.89 (m, 1H), 7.59 – 7.44 (m, 3H), 7.40 (t, *J* = 7.8 Hz, 1H), 7.33 (d, *J* = 7.4 Hz, 1H), 7.22 (t, *J* = 7.9 Hz, 2H), 7.13 – 6.99 (m, 2H), 6.82 (t, *J* = 7.3 Hz, 1H).

^{13}C NMR (100 MHz, DMSO) δ 145.0, 139.4, 134.4, 129.0, 128.1, 127.0, 126.1, 126.0, 125.0, 122.8, 121.4, 119.5, 117.0, 114.1.

3-Chloro-*N*-phenylisoquinolin-5-amine (5)

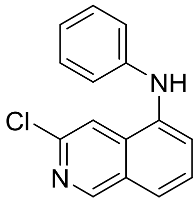

Prepared according to the **general procedure 3** on 0.5 mmol scale and obtained an isolated yield of 75% (96 mg) as a yellow solid.

m.p.: 157-159 °C.

^{1}H NMR (400 MHz, DMSO) δ 9.16 (s, 1H), 8.45 (s, 1H), 8.25 (s, 1H), 7.67 (m, 1H), 7.60 – 7.50 (m, 2H), 7.31 (t, *J* = 7.9 Hz, 2H), 7.23 – 7.13 (m, 2H), 6.95 (t, *J* = 7.3 Hz, 1H).

^{13}C NMR (100 MHz, DMSO) δ 153.1, 144.0, 143.1, 138.7, 130.4, 129.2, 128.4, 128.3, 121.1, 119.3, 118.5, 115.5, 115.0.

HRMS (ESI) calculated for $C_{15}H_{12}N_2Cl$ $[M+H]^+$ 255.0689, found 255.0692.

Part 5. Synthesis and characterization of products from hydrogenation reaction.

General procedure 4: Synthesis of hydrogenation products.

To a stirred solution of 9-phenyl-1,4-dihydro-1,4-epiminonaphthalene (0.5 mmol) in EtOH (20 mL) was carefully added wet Pd/C catalyst (0.05 mmol) under argon atmosphere. The tube was degassed with hydrogen for three times. The mixture was stirred at room temperature until TLC indicated that the starting materials were completely consumed. Then the solid was filtered off and the filtrate was concentrated in vacuo. The crude products were purified using flash column chromatography on silica gel to afford the desired product.

9-Phenyl-1,2,3,4-tetrahydro-1,4-epiminonaphthalene (6)

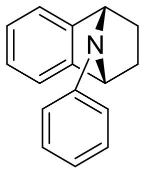

Prepared according to the **general procedure 4** on 0.5 mmol scale and obtained an isolated yield of 63% (70 mg) as a yellow oil.

^{1}H NMR (400 MHz, $CDCl_3$) δ 7.29 – 7.19 (m, 2H), 7.19 – 7.05 (m, 4H), 6.94 – 6.81 (m, 2H), 6.81 – 6.70 (m, 1H), 5.06 (dd, *J* = 2.5, 1.8 Hz, 2H), 2.26 – 2.08 (m, 2H), 1.37 – 1.29 (m, 2H).

^{13}C NMR (100 MHz, $CDCl_3$) δ 147.4, 145.0, 129.0, 126.4, 120.1, 120.1, 117.2, 63.7, 26.0.

HRMS (ESI) calculated for $C_{16}H_{16}N$ $[M+H]^+$ 222.1283, found 222.1277.

Part 6. References

1. Sundalam, S. K.; Nilova, A.; Seidl, T. L.; Stuart, D. R. *Angew. Chem. Int. Ed.* **2016**, *55*, 8431-8434.
2. Li, J.; Wang, H.; Sun, J.; Yang, Y.; Liu, L. *Org. Biomol. Chem.* **2014**, *12*, 7904-7908.
3. Allen, A. E.; MacMillan, D. C. *J. Am. Chem. Soc.* **2011**, *133*, 4260-4263.
4. Xu, Z.; Li, H.; Ren, Z.; Du, W.; Xu, W.; Lang, J. *Tetrahedron* **2011**, *67*, 5282-5288.
5. Li, Z.; Meng, F.; Zhang, J.; Xie, J.; Dai, B. *Org. Biomol. Chem.* **2016**, *14*, 10861-10865.
6. So, C. M.; Zhou, Z.; Lau, C. P.; Kwong, F. Y. *Angew. Chem. Int. Ed.* **2008**, *47*, 6402-6406.
7. Sarvari, M. H.; Razmi, Z. *RSC Adv.* **2014**, *4*, 44105-44116.
8. Naeimi, H.; Dadaei, M. *RSC Adv.* **2015**, *5*, 76221-76228.
9. Sreedhar, B.; Arundhathi, R.; Reddy, P. L.; Kantam, M. L. *J. Org. Chem.* **2009**, *74*, 7951-7954.
10. Topchiy, M. A.; Dzhevakov, P. B.; Rubina, M. S.; Morozov, O. S.; Asachenko, A. F.; Nechaev, M. S. *Eur. J. Org. Chem.* **2016**, *2016*, 1908-1914.

Part 7. Copies of ^{1}H, ^{13}C, ^{19}F NMR and HRMS (ESI) spectra

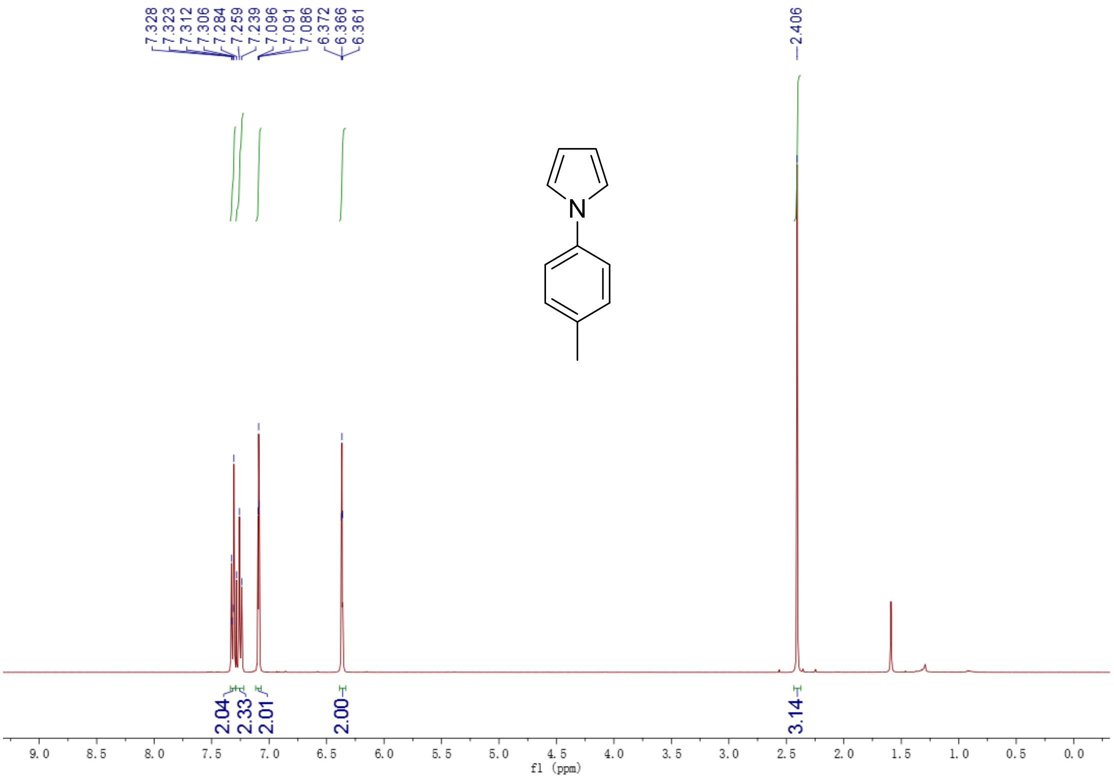

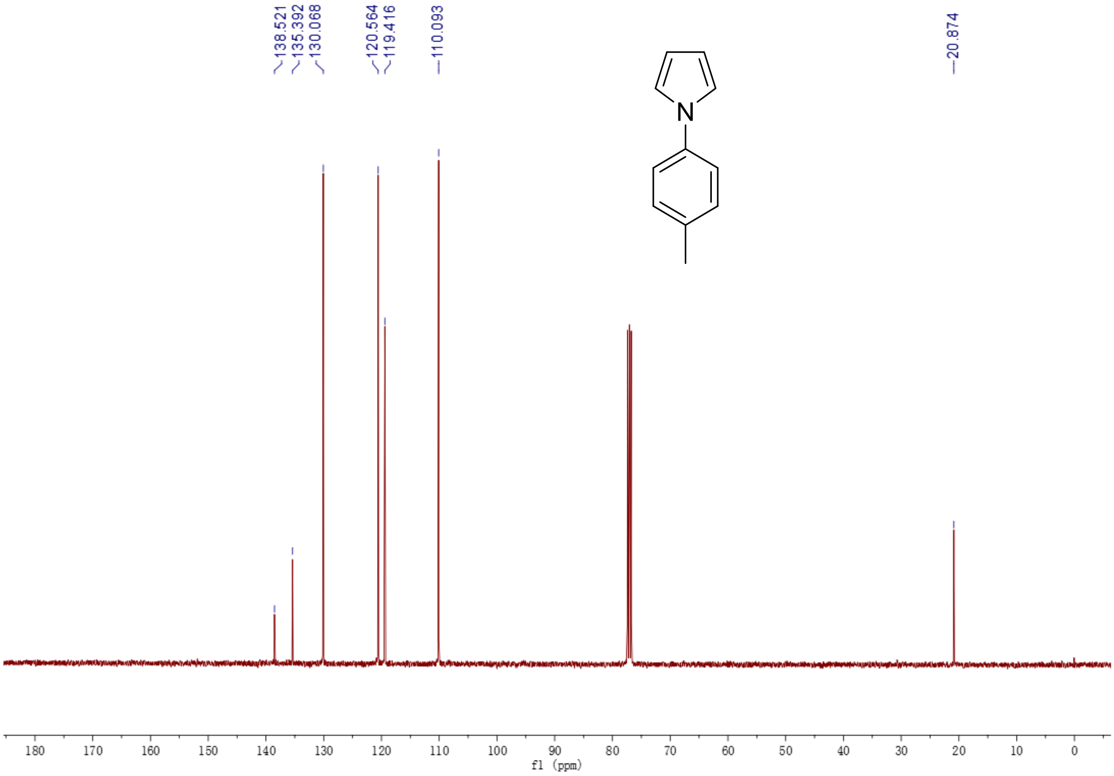

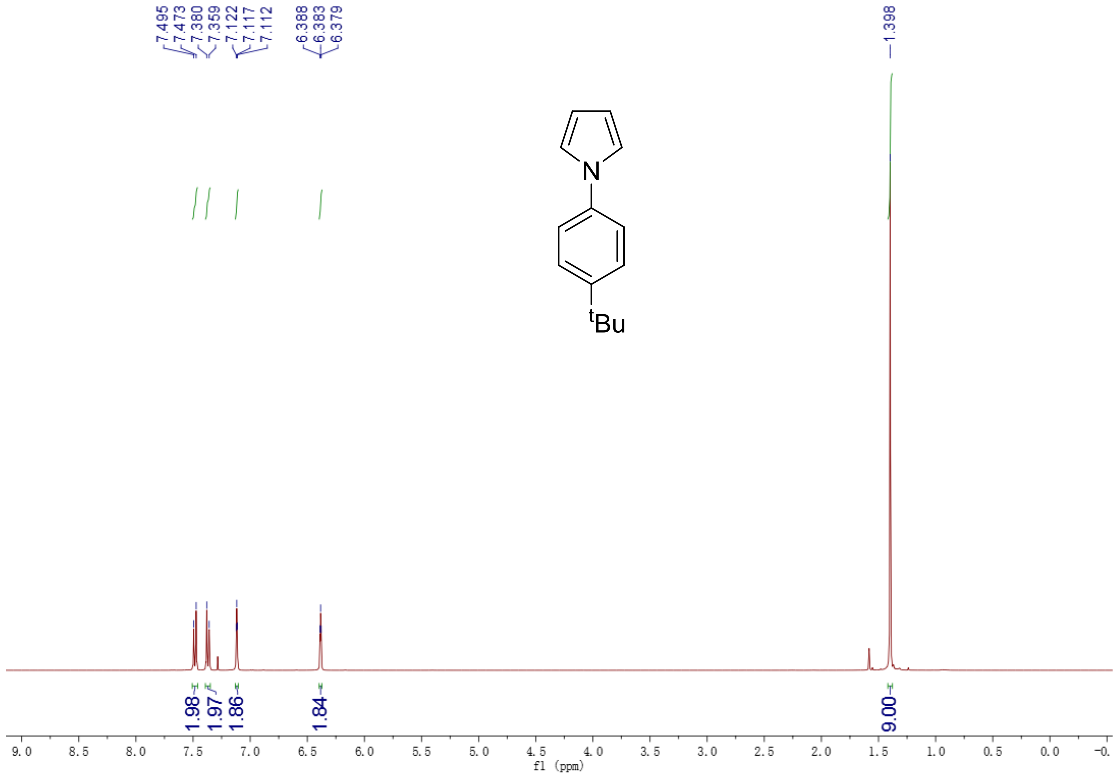
7.495
7.473
7.380
7.359
7.122
7.117
7.112
6.388
6.383
6.379
1.398
N
tBu
1.98
1.97
1.86
1.84
9.00
9.0 8.5 8.0 7.5 7.0 6.5 6.0 5.5 5.0 4.5 4.0 3.5 3.0 2.5 2.0 1.5 1.0 0.5 0.0
f1 (ppm)

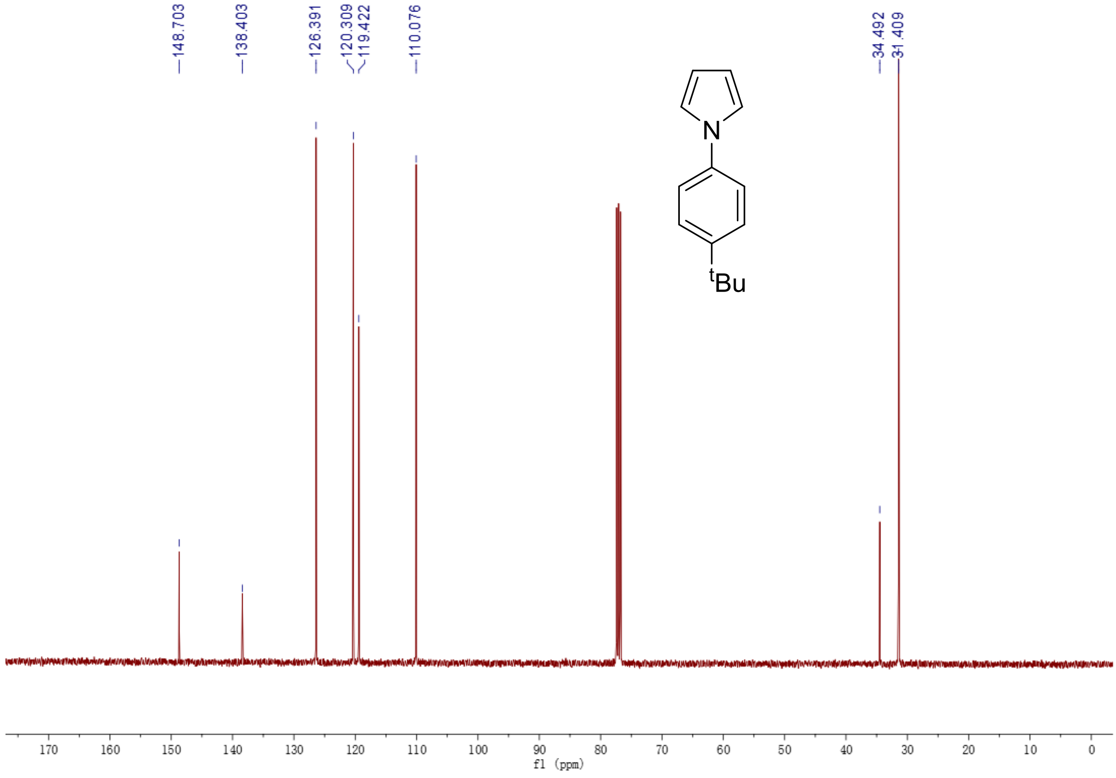
148.703
138.403
126.391
120.309
119.422
110.076
34.492
31.409
N
tBu
170 160 150 140 130 120 110 100 90 80 70 60 50 40 30 20 10 0
f1 (ppm)

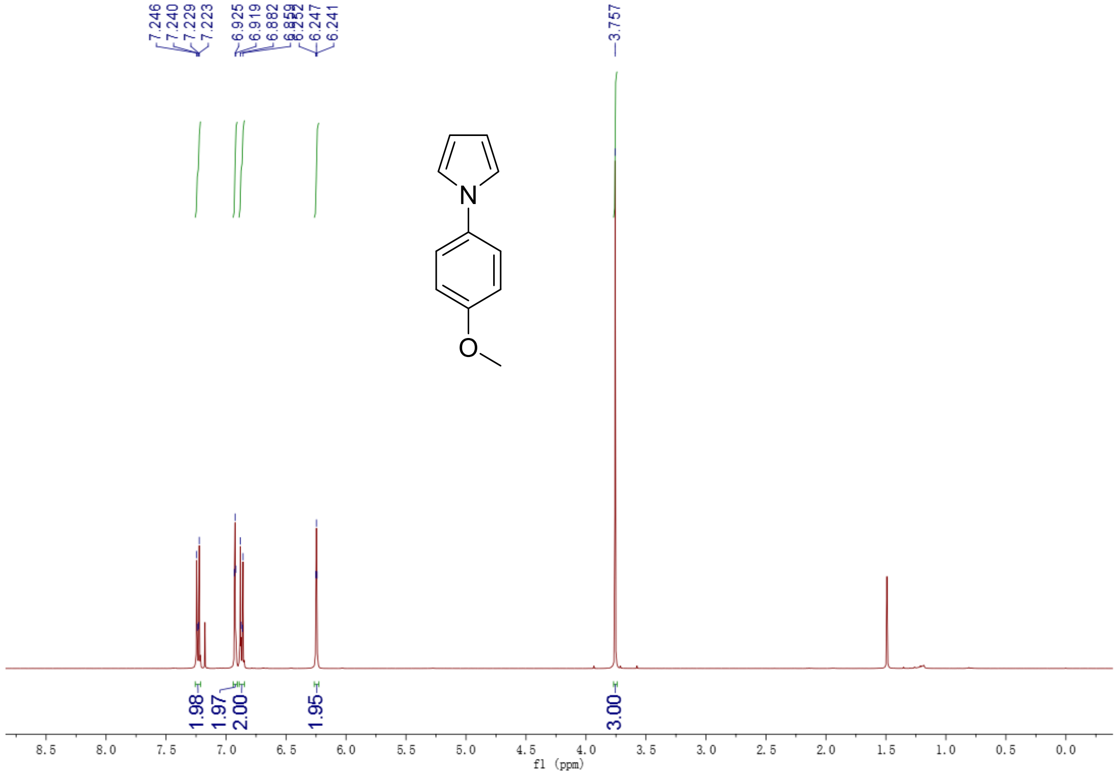
7.246
7.240
7.229
7.223
6.925
6.919
6.882
6.247
6.241
3.757
N
O
1.98
1.97
2.00
1.95
3.00
f1 (ppm)

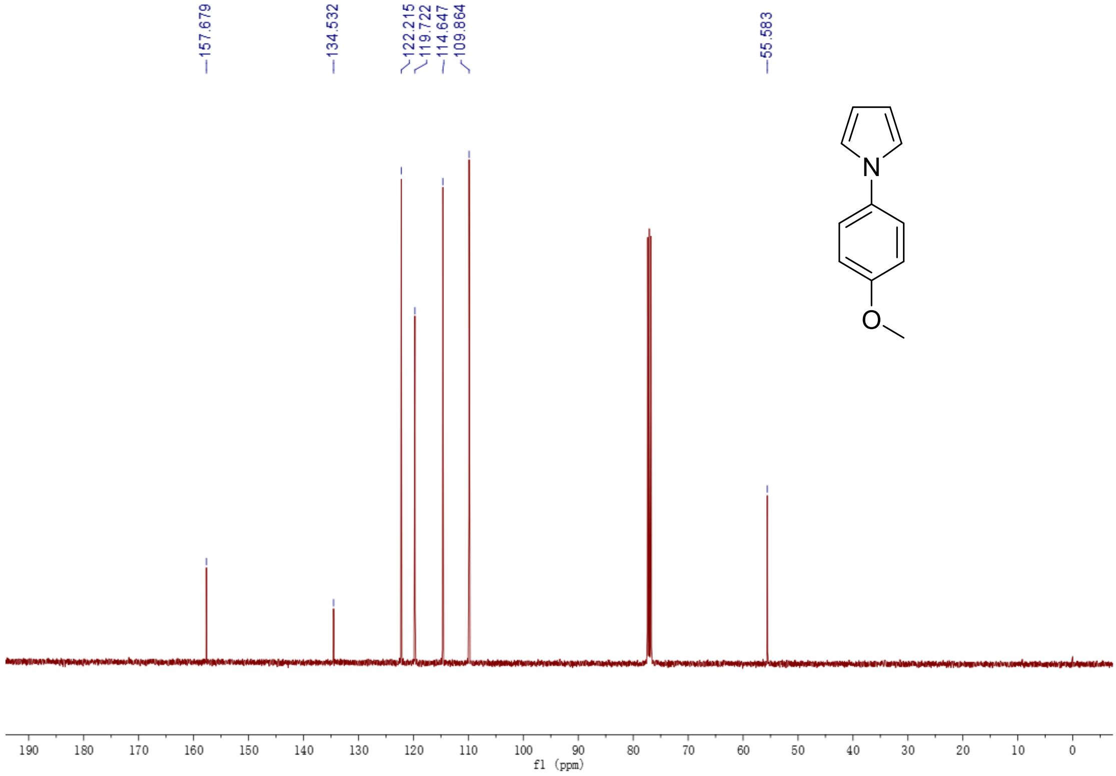
157.679
134.532
122.215
119.722
114.647
109.864
55.583
N
O
f1 (ppm)

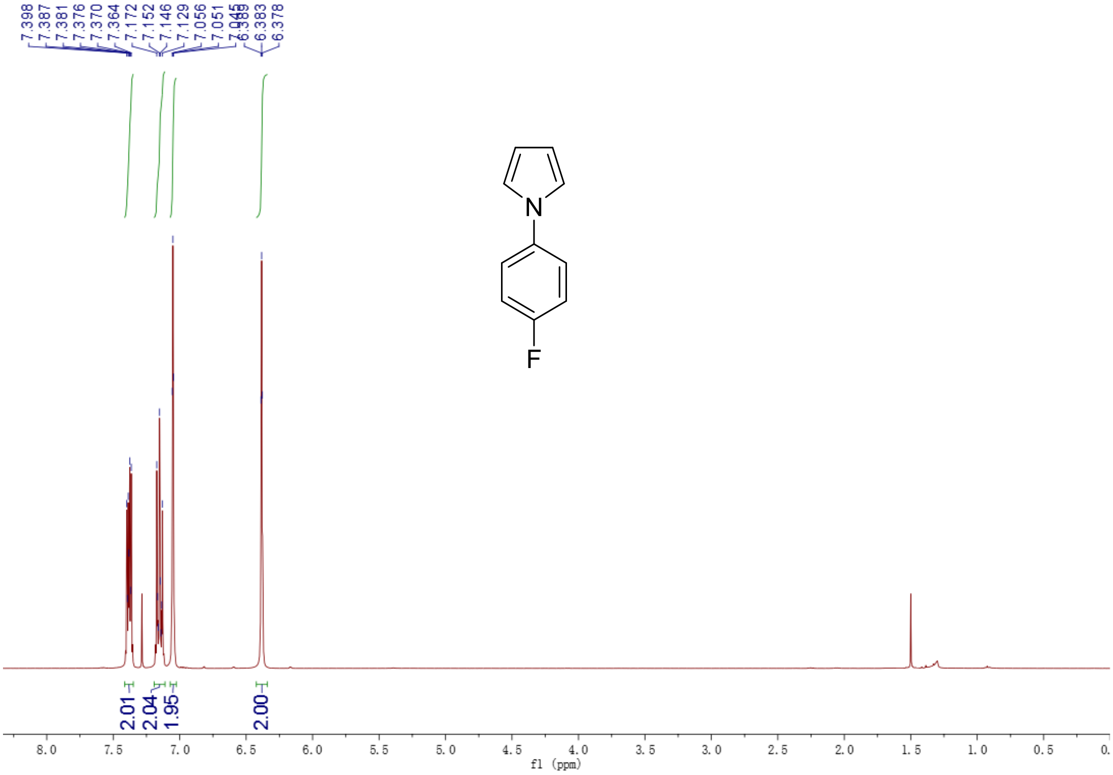
7.398
7.387
7.381
7.376
7.370
7.364
7.172
7.152
7.146
7.129
7.056
7.051
6.383
6.378
N
F
2.01
2.04
1.95
2.00
8.0
7.5
7.0
6.5
6.0
5.5
5.0
4.5
4.0
3.5
3.0
2.5
2.0
1.5
1.0
0.5
f1 (ppm)

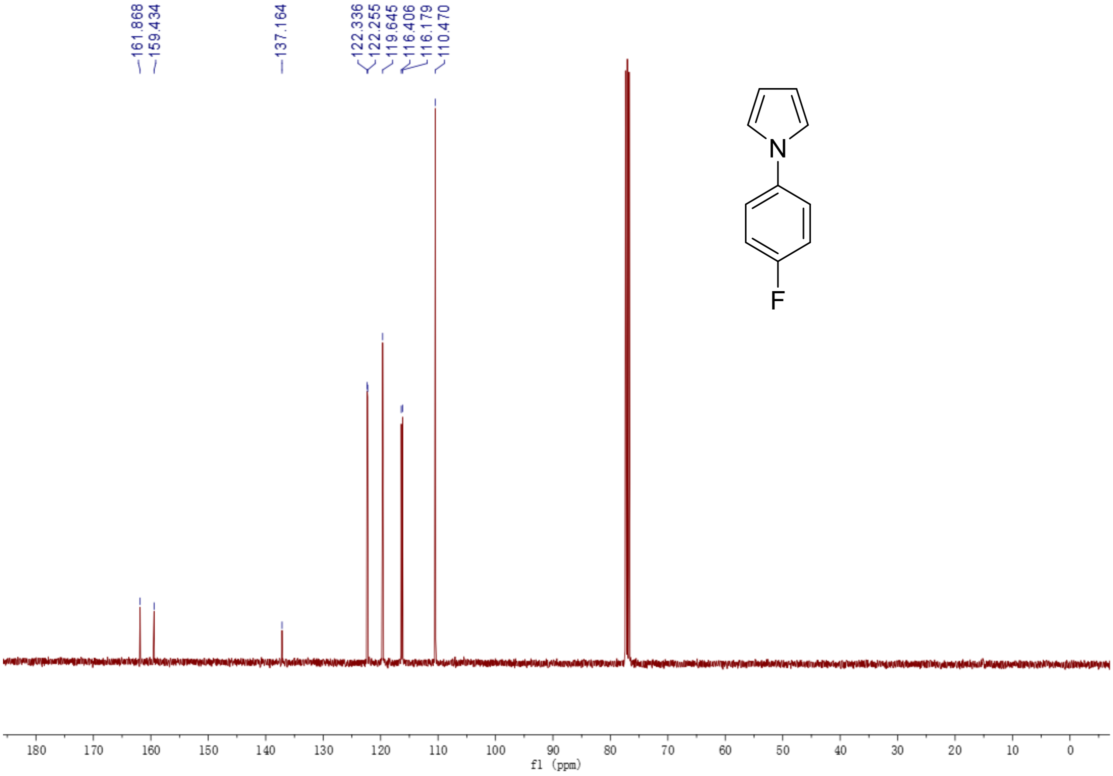
161.868
159.434
137.164
122.336
122.255
119.645
116.406
116.179
110.470
N
F
180
170
160
150
140
130
120
110
100
90
80
70
60
50
40
30
20
10
0
f1 (ppm)

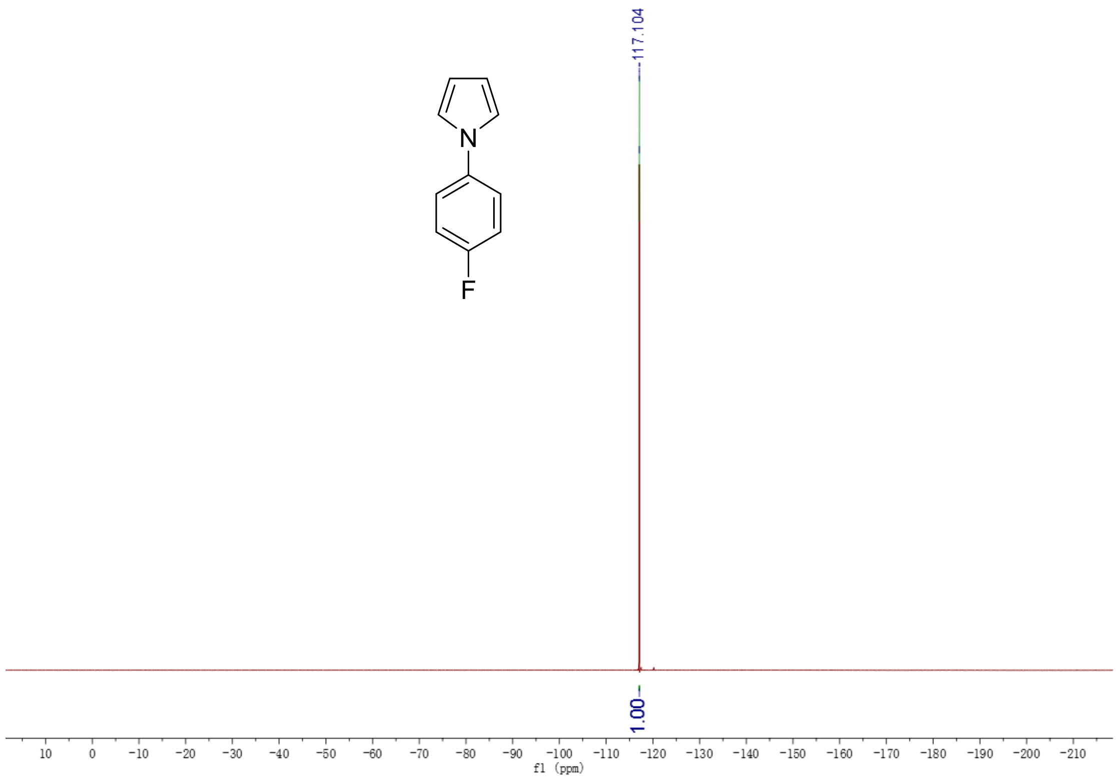
-117.104
N
F
1.00
10 0 −10 −20 −30 −40 −50 −60 −70 −80 −90 −100 −110 −120 −130 −140 −150 −160 −170 −180 −190 −200 −210
f1 (ppm)

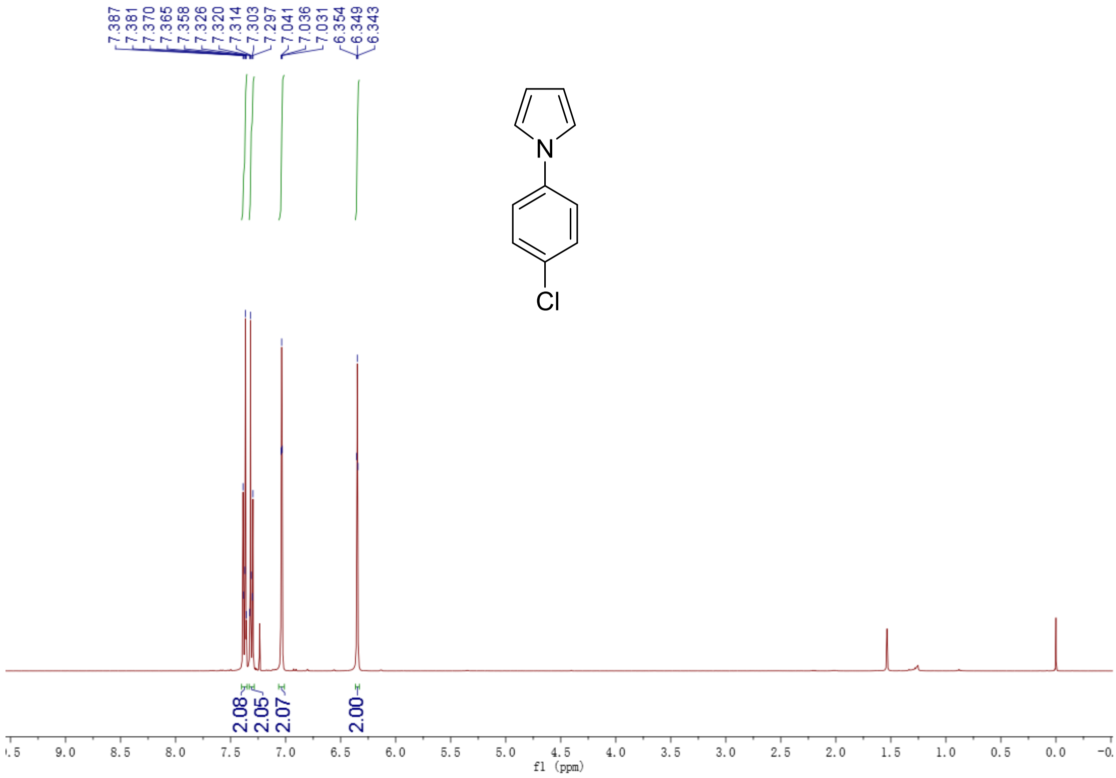
7.387 7.381 7.370 7.365 7.358 7.326 7.320 7.314 7.303 7.297 7.041 7.036 7.031 6.354 6.349 6.343
N
Cl
2.08 2.05 2.07 2.00
9.0 8.5 8.0 7.5 7.0 6.5 6.0 5.5 5.0 4.5 4.0 3.5 3.0 2.5 2.0 1.5 1.0 0.5 0.0
f1 (ppm)

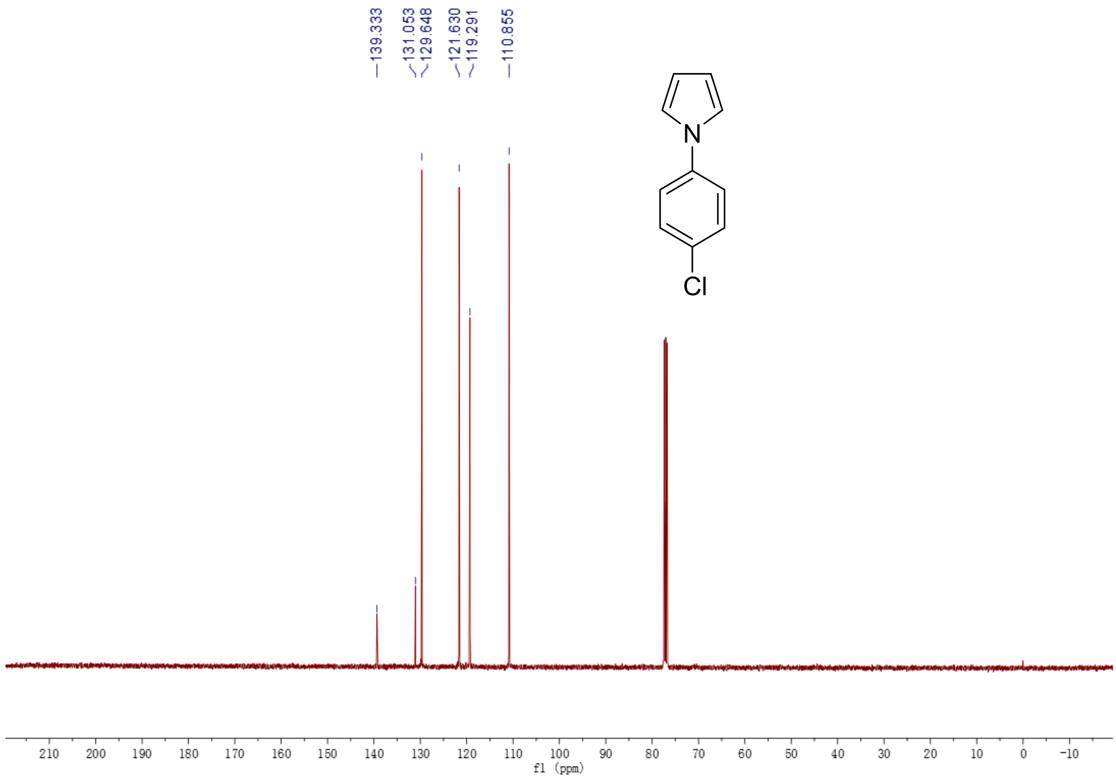
139.333
131.053
129.648
121.630
119.291
110.855
N
Cl
210 200 190 180 170 160 150 140 130 120 110 100 90 80 70 60 50 40 30 20 10 0 -10
f1 (ppm)

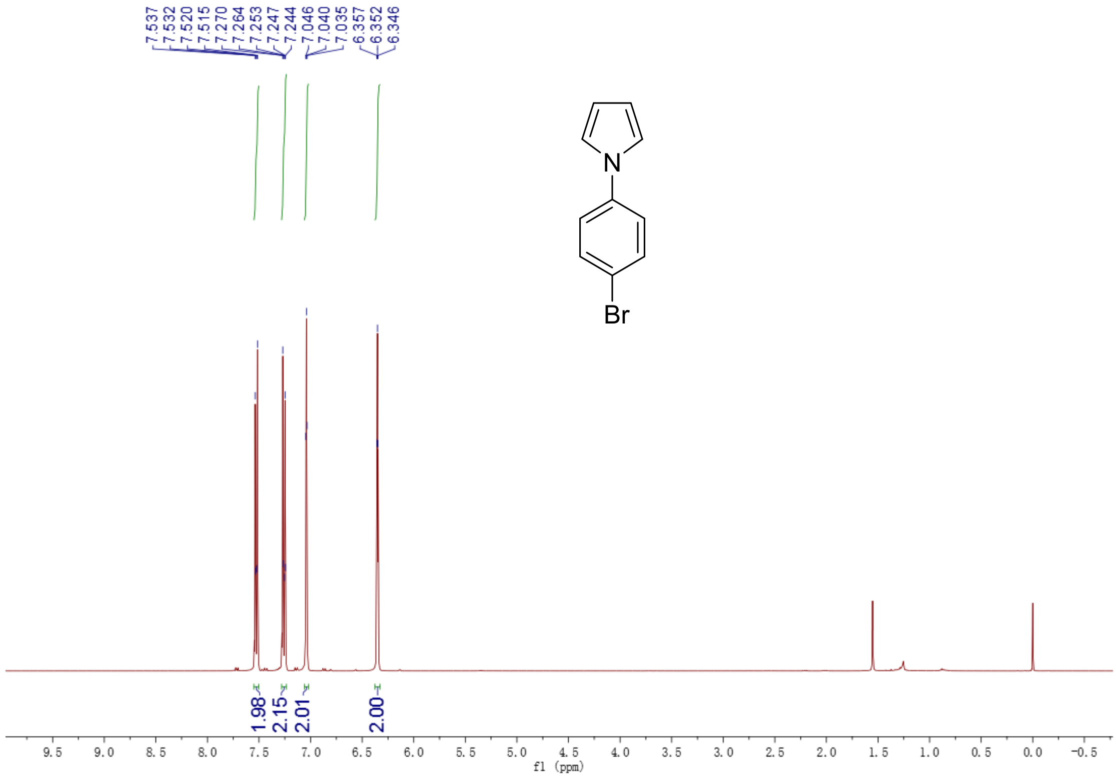
7.537
7.532
7.520
7.515
7.270
7.264
7.253
7.247
7.244
7.046
7.040
7.035
6.357
6.352
6.346
N
Br
1.98
2.15
2.01
2.00
9.5 9.0 8.5 8.0 7.5 7.0 6.5 6.0 5.5 5.0 4.5 4.0 3.5 3.0 2.5 2.0 1.5 1.0 0.5 0.0 -0.5
f1 (ppm)

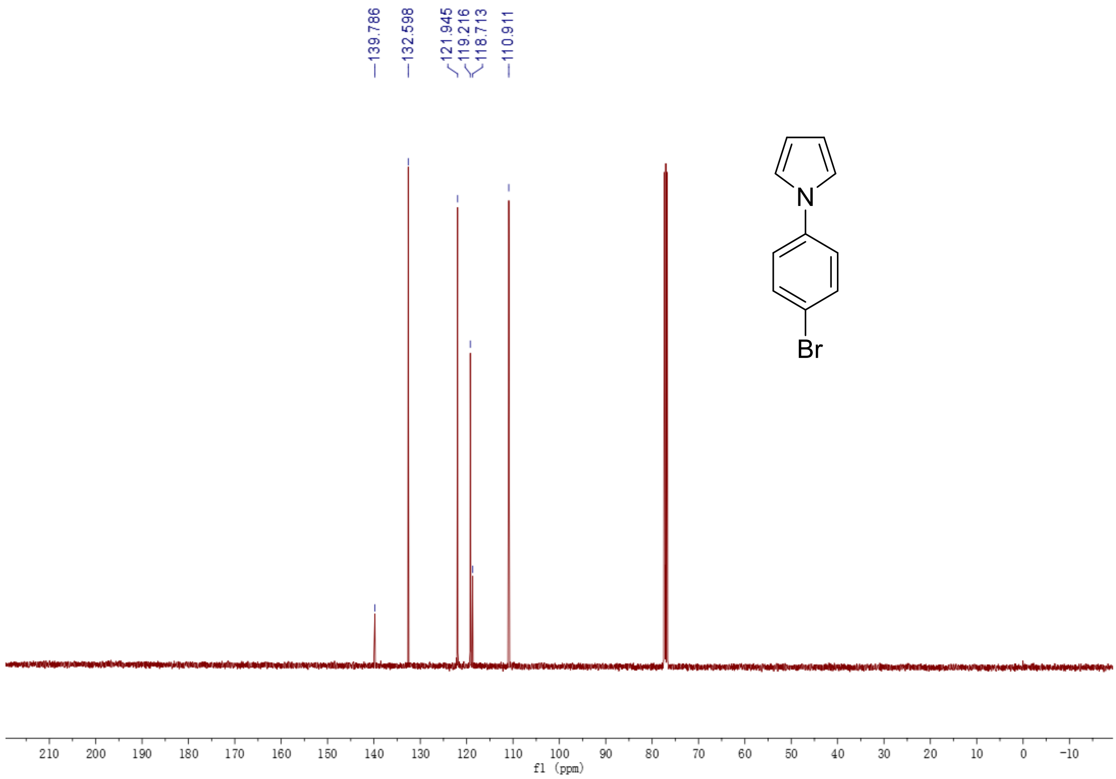
139.786
132.598
121.945
119.216
118.713
110.911
N
Br
210 200 190 180 170 160 150 140 130 120 110 100 90 80 70 60 50 40 30 20 10 0 -10
f1 (ppm)

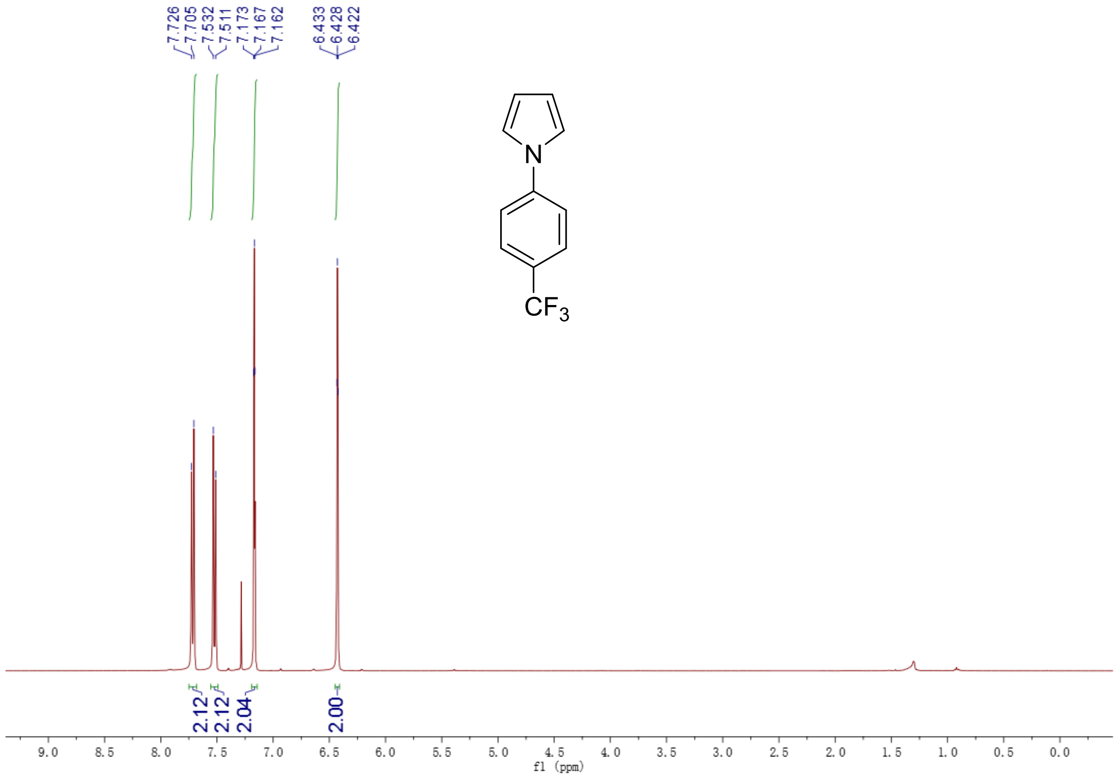
7.726
7.705
7.532
7.511
7.173
7.167
7.162
6.433
6.428
6.422
N
CF_3
2.12
2.12
2.04
2.00
9.0 8.5 8.0 7.5 7.0 6.5 6.0 5.5 5.0 4.5 4.0 3.5 3.0 2.5 2.0 1.5 1.0 0.5 0.0
f1 (ppm)

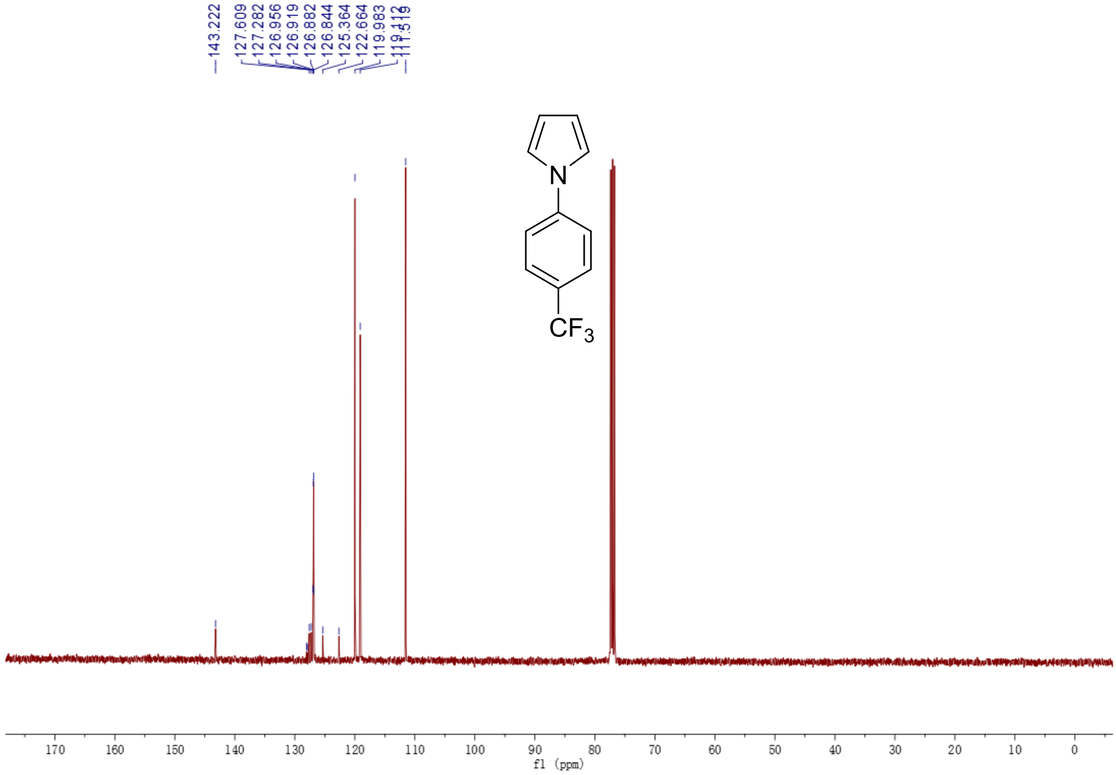
143.222
127.609
127.282
126.956
126.919
126.882
126.844
125.364
122.664
119.983
119.112
111.519
N
CF3
170 160 150 140 130 120 110 100 90 80 70 60 50 40 30 20 10 0
f1 (ppm)

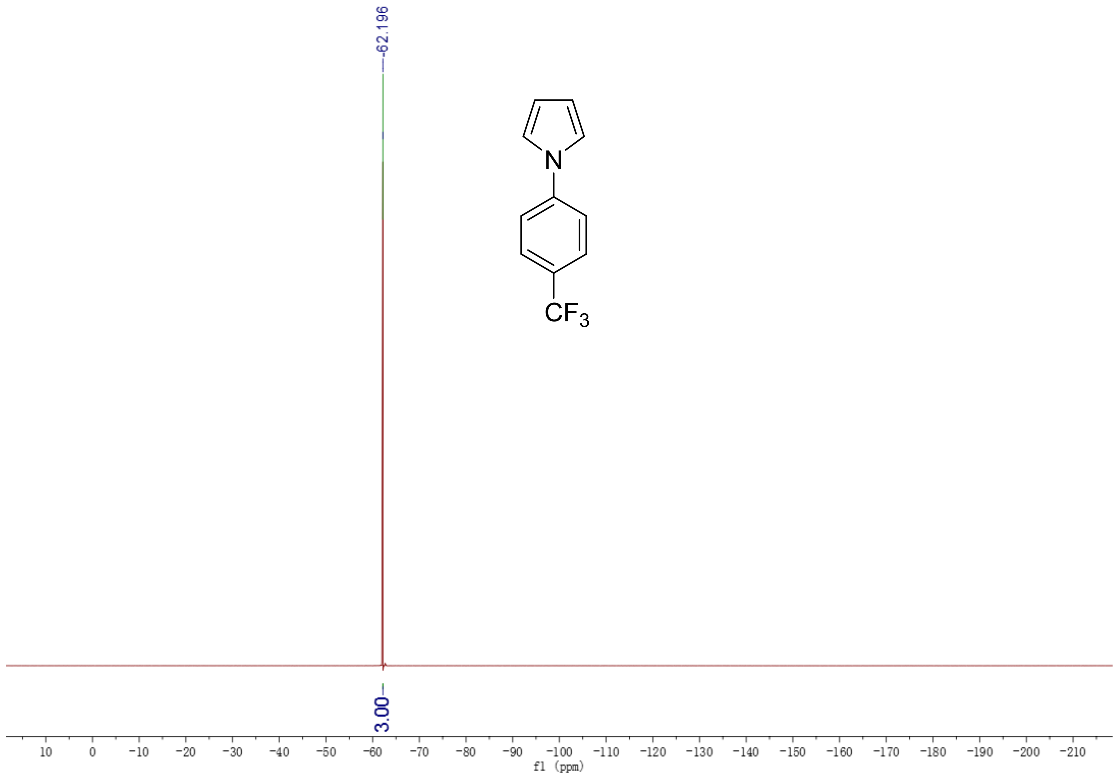
-62.196
N
CF3
3.00
10 0 −10 −20 −30 −40 −50 −60 −70 −80 −90 −100 −110 −120 −130 −140 −150 −160 −170 −180 −190 −200 −210
f1 (ppm)

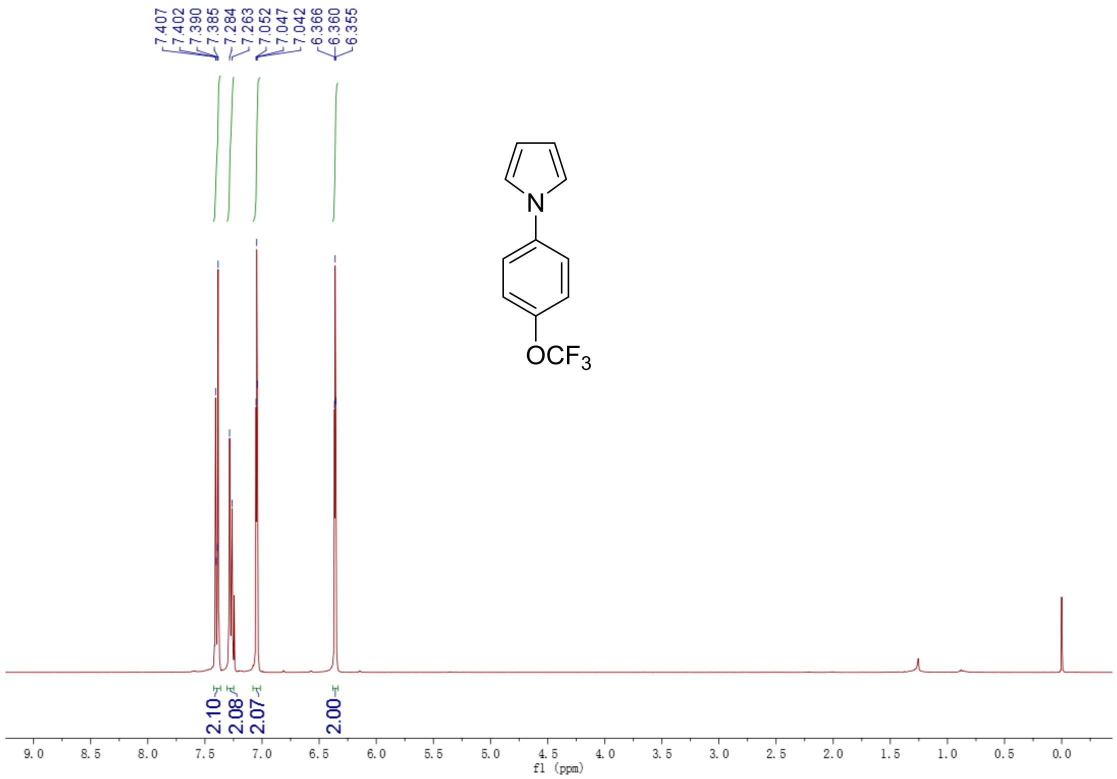
7.407
7.402
7.390
7.385
7.284
7.263
7.052
7.047
7.042
6.366
6.360
6.355
N
OCF3
2.10
2.08
2.07
2.00
9.0 8.5 8.0 7.5 7.0 6.5 6.0 5.5 5.0 4.5 4.0 3.5 3.0 2.5 2.0 1.5 1.0 0.5 0.0
f1 (ppm)

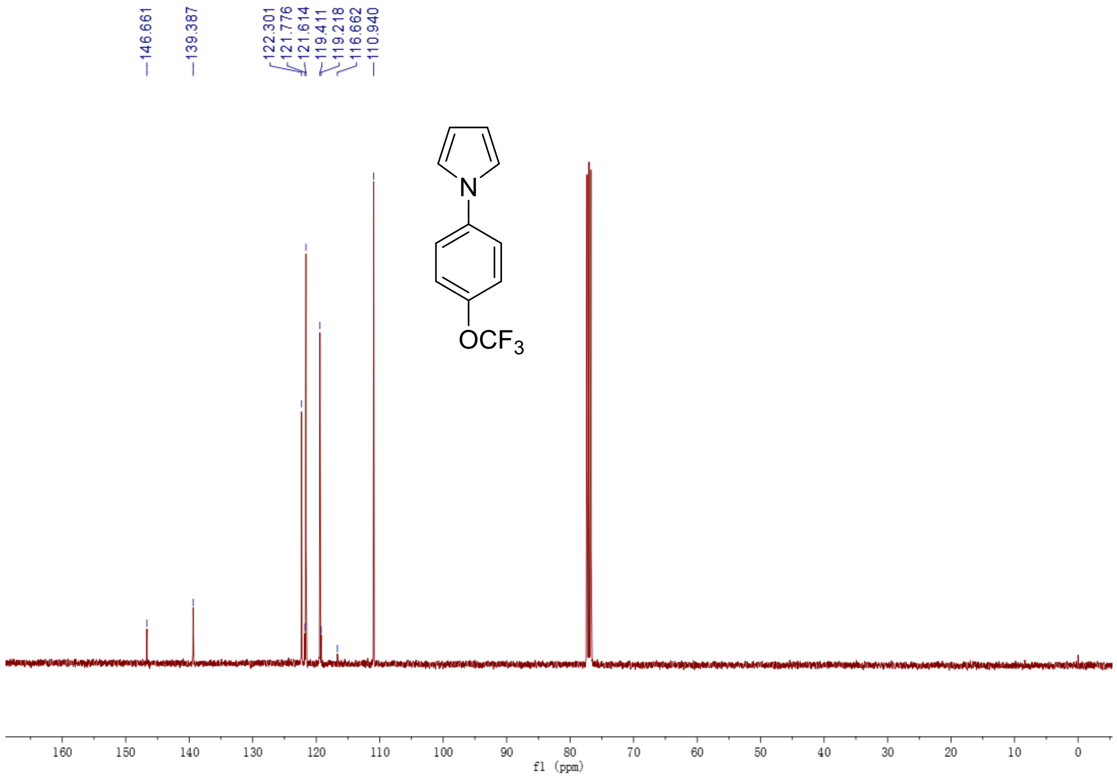
146.661
139.387
122.301
121.776
121.614
119.411
119.218
116.662
110.940
N
OCF3
160 150 140 130 120 110 100 90 80 70 60 50 40 30 20 10 0
f1 (ppm)

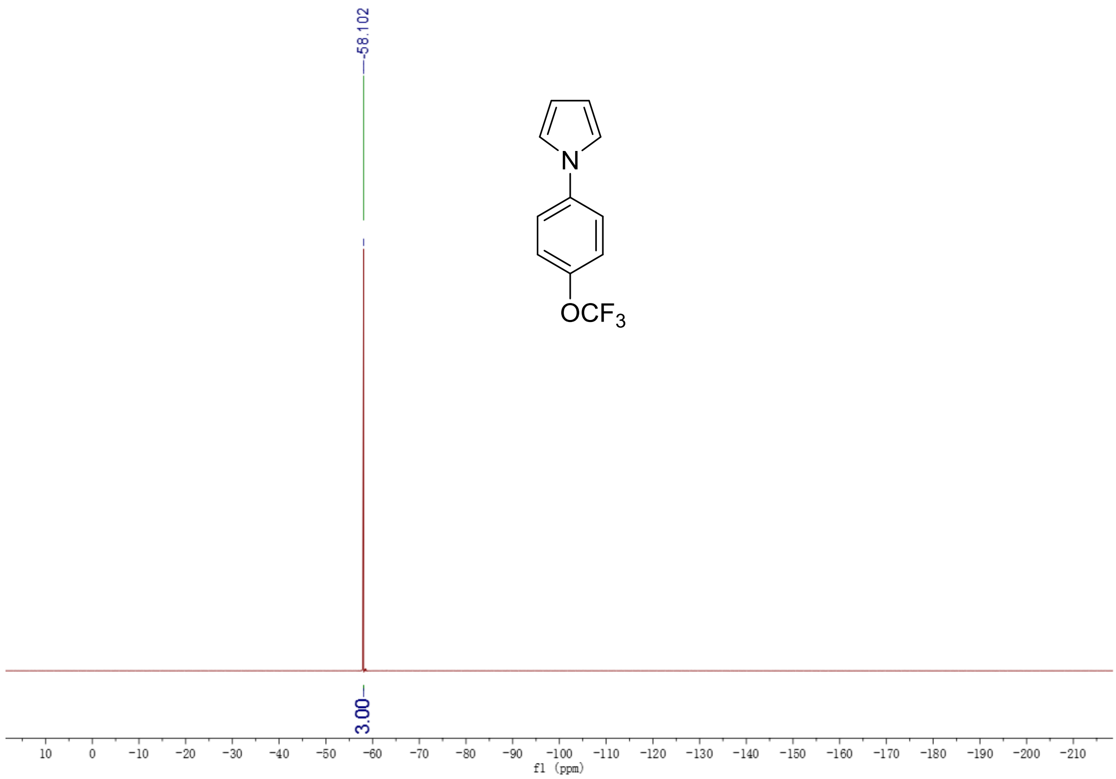
-58.102
OCF3
3.00
10
0
-10
-20
-30
-40
-50
-60
-70
-80
-90
-100
-110
-120
-130
-140
-150
-160
-170
-180
-190
-200
-210
f1 (ppm)

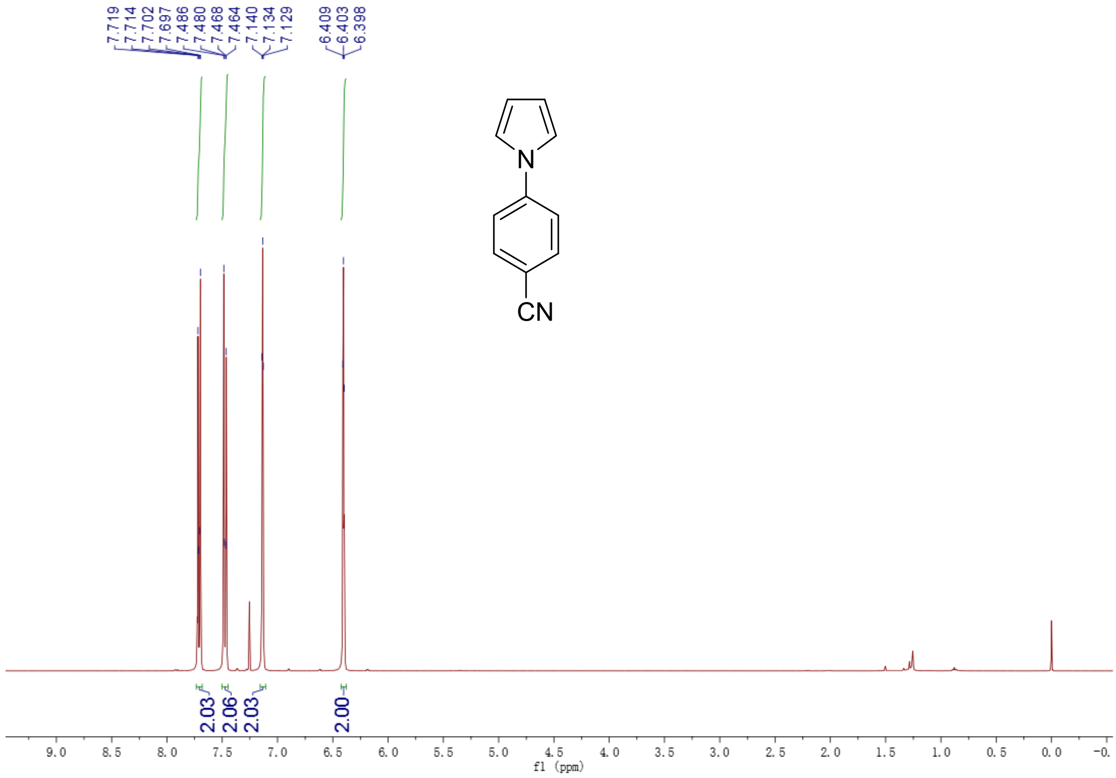
7.719
7.714
7.702
7.697
7.486
7.480
7.468
7.464
7.140
7.134
7.129
6.409
6.403
6.398
CN
2.03
2.06
2.03
2.00
9.0
8.5
8.0
7.5
7.0
6.5
6.0
5.5
5.0
4.5
4.0
3.5
3.0
2.5
2.0
1.5
1.0
0.5
0.0
f1 (ppm)

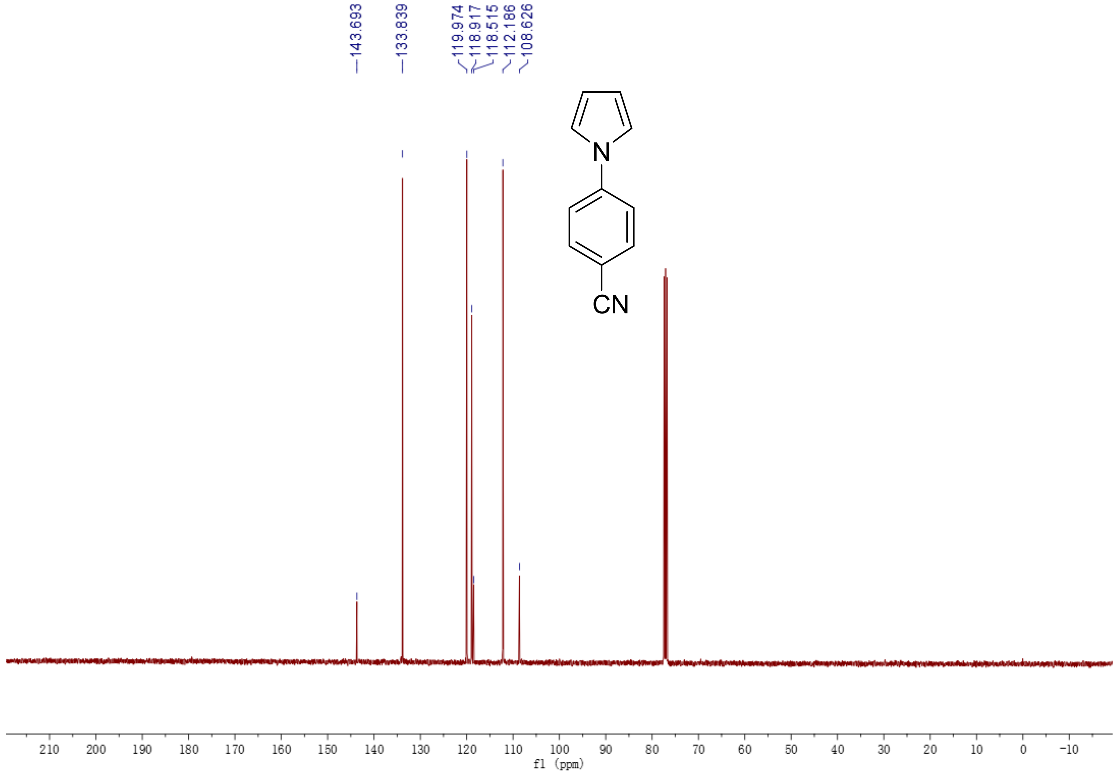
143.693
133.839
119.974
118.917
118.515
112.186
108.626
N
CN
210 200 190 180 170 160 150 140 130 120 110 100 90 80 70 60 50 40 30 20 10 0 -10
f1 (ppm)

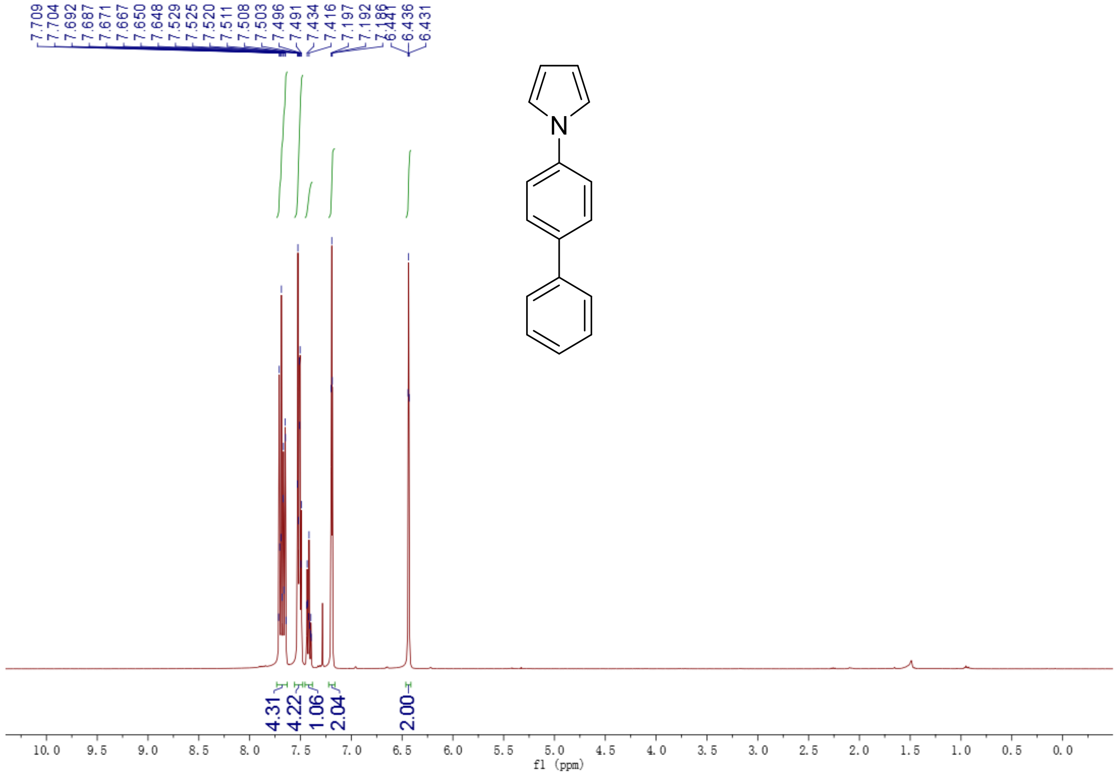
7.709
7.704
7.692
7.687
7.671
7.667
7.650
7.648
7.529
7.525
7.520
7.511
7.508
7.503
7.496
7.491
7.434
7.416
7.197
7.192
6.436
6.431
N
4.31
4.22
1.06
2.04
2.00
10.0 9.5 9.0 8.5 8.0 7.5 7.0 6.5 6.0 5.5 5.0 4.5 4.0 3.5 3.0 2.5 2.0 1.5 1.0 0.5 0.0
f1 (ppm)

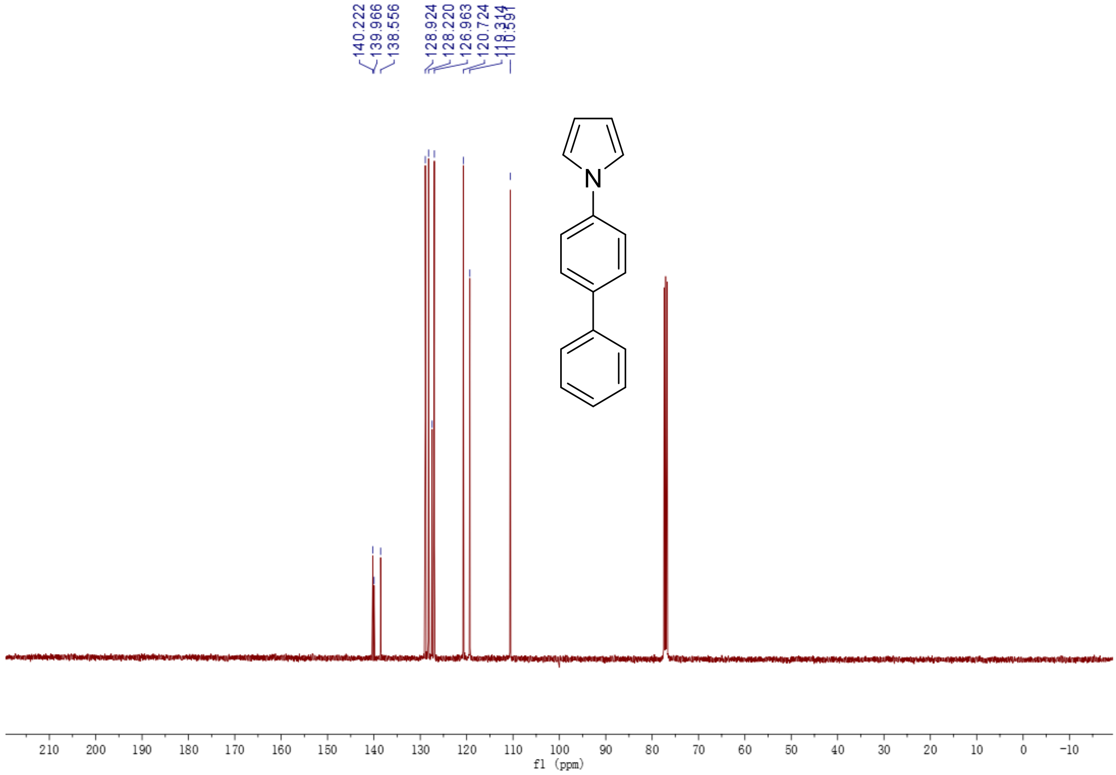
140.222
139.966
138.556
128.924
128.220
126.963
120.724
119.314
110.591
N
f1 (ppm)

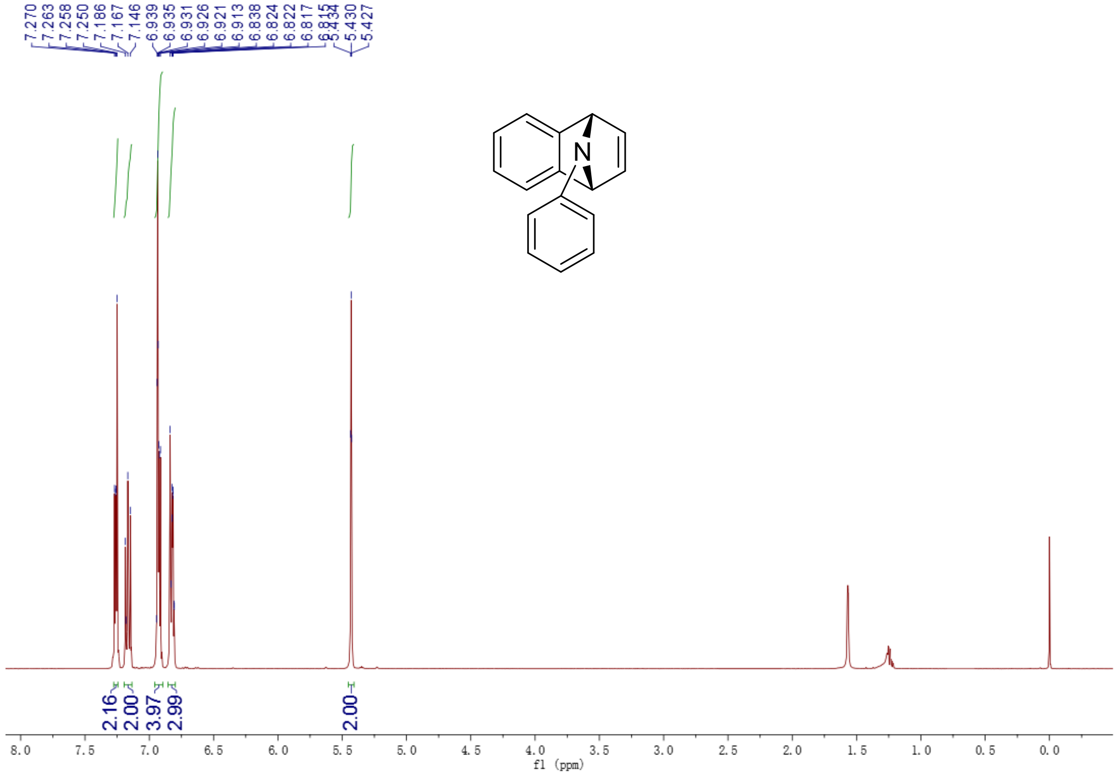
7.270
7.263
7.258
7.250
7.186
7.167
7.146
6.939
6.935
6.931
6.926
6.921
6.913
6.838
6.824
6.822
6.817
6.815
5.434
5.430
5.427
N
2.16
2.00
3.97
2.99
2.00
f1 (ppm)

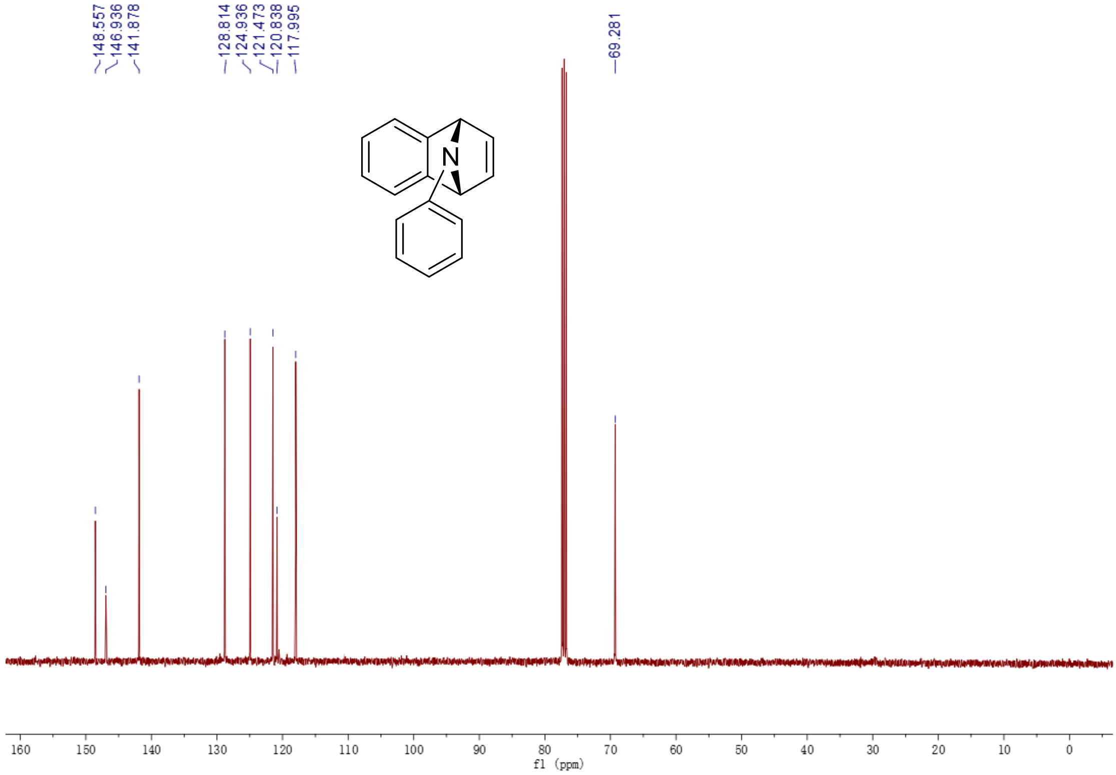
148.557
146.936
141.878
128.814
124.936
121.473
120.838
117.995
69.281
N
f1 (ppm)

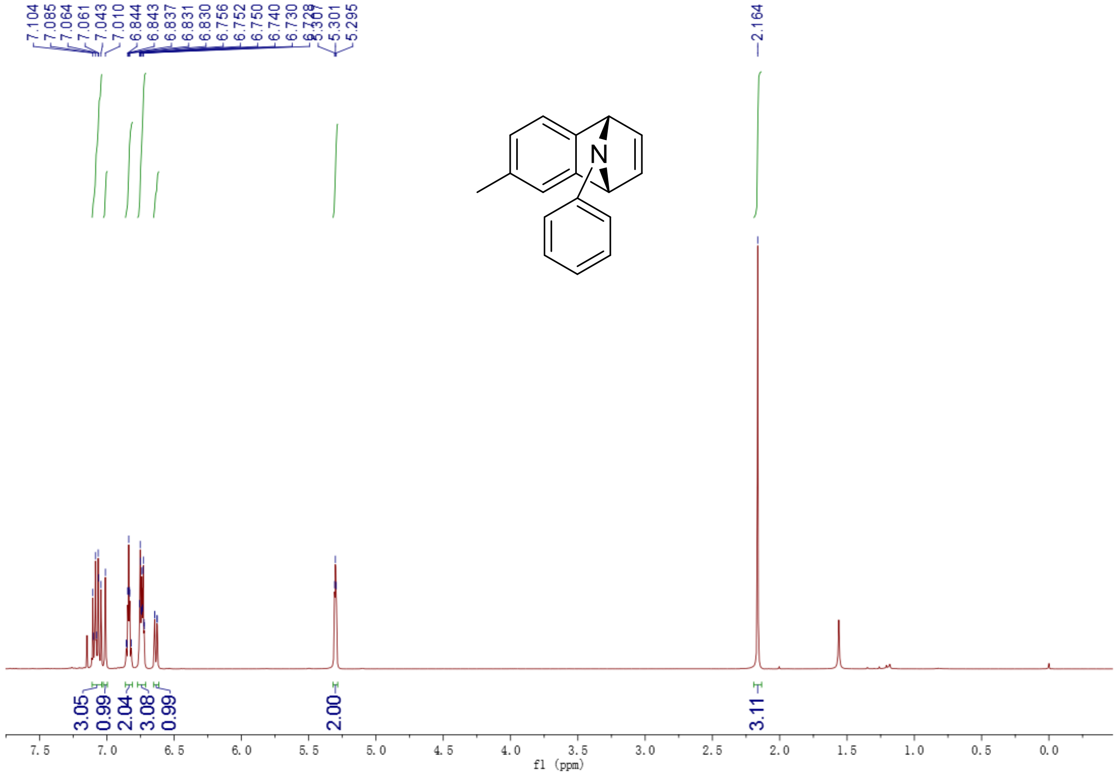
7.104
7.085
7.064
7.061
7.043
7.010
6.844
6.843
6.837
6.831
6.830
6.756
6.752
6.750
6.740
6.730
5.307
5.301
5.295
2.164
N
3.05
0.99
2.04
3.08
0.99
2.00
3.11
f1 (ppm)

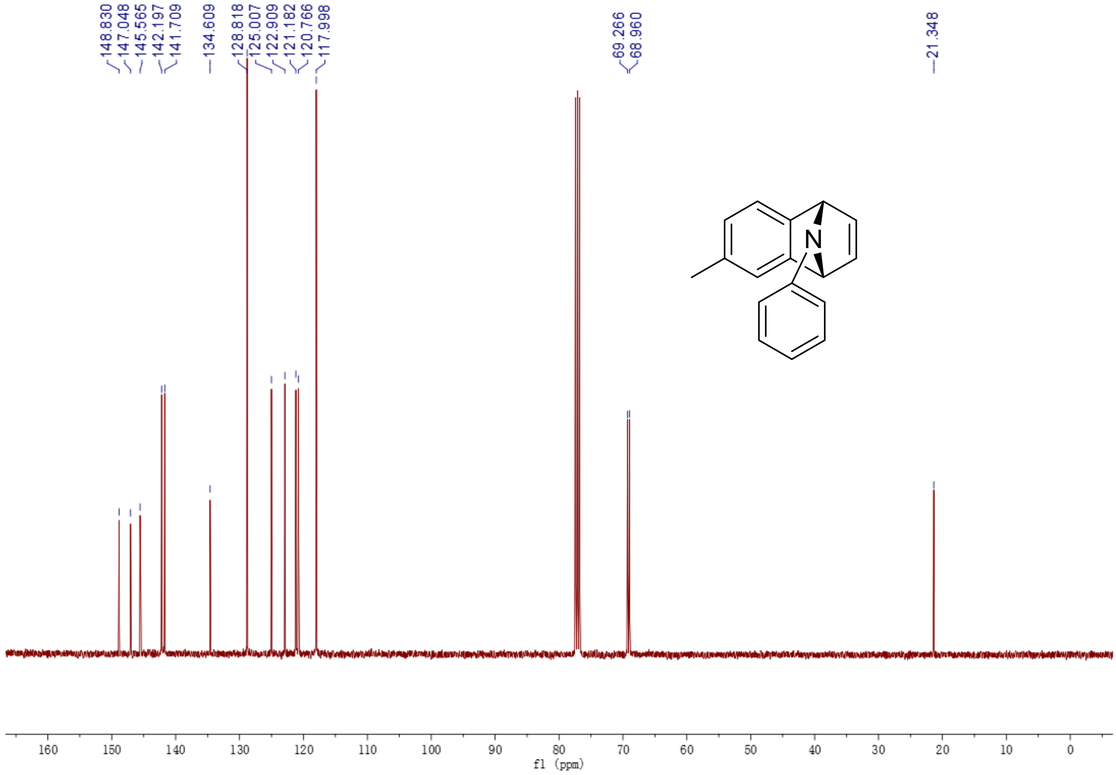
148.830
147.048
145.565
142.197
141.709
134.609
128.818
125.007
122.909
121.182
120.766
117.998
69.266
68.960
21.348
N
160 150 140 130 120 110 100 90 80 70 60 50 40 30 20 10 0
f1 (ppm)

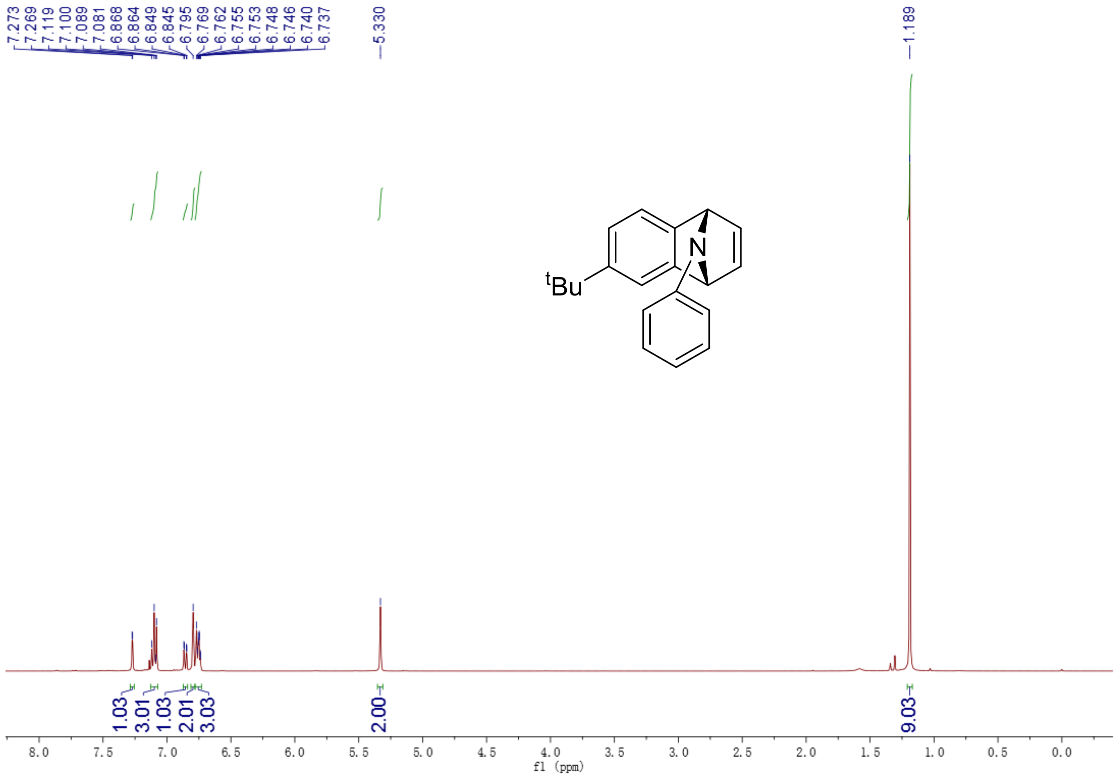
7.273
7.269
7.119
7.100
7.089
7.081
6.868
6.864
6.849
6.845
6.795
6.769
6.762
6.755
6.753
6.748
6.746
6.740
6.737
5.330
1.189
N
tBu
1.03
3.01
1.03
2.01
3.03
2.00
9.03
8.0 7.5 7.0 6.5 6.0 5.5 5.0 4.5 4.0 3.5 3.0 2.5 2.0 1.5 1.0 0.5 0.0
f1 (ppm)

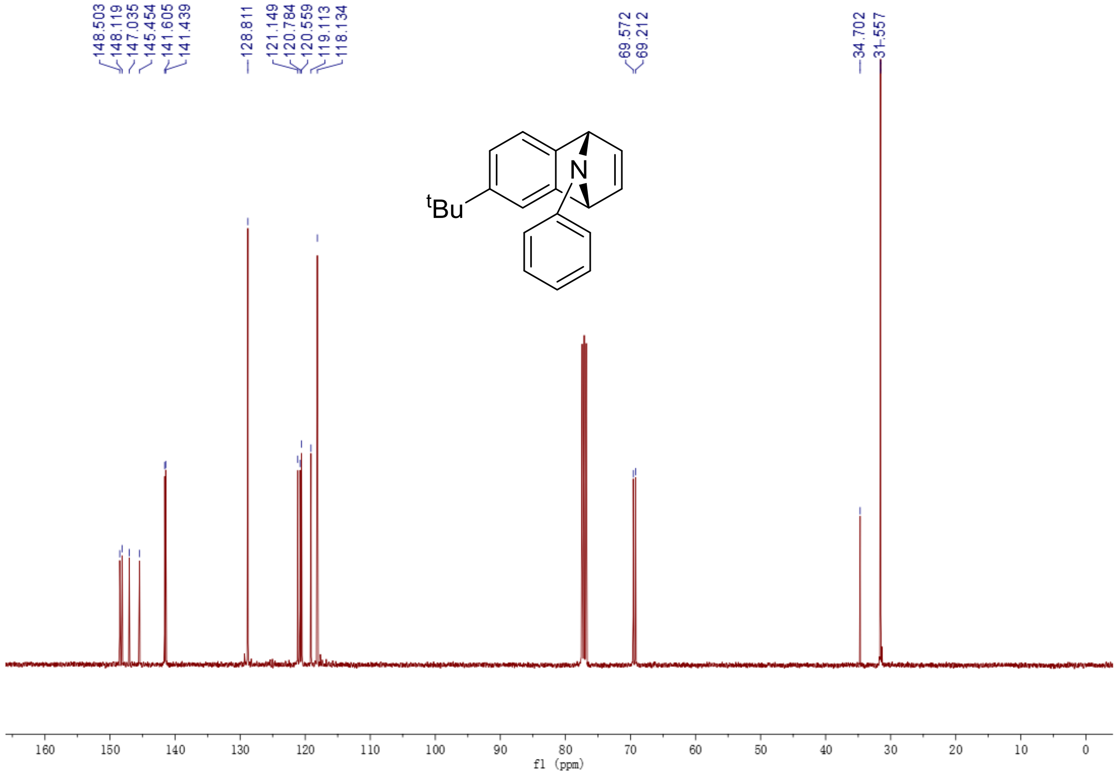
148.503
148.119
147.035
145.454
141.605
141.439
128.811
121.149
120.784
120.559
119.113
118.134
69.572
69.212
34.702
31.557
tBu
N
160 150 140 130 120 110 100 90 80 70 60 50 40 30 20 10 0
f1 (ppm)

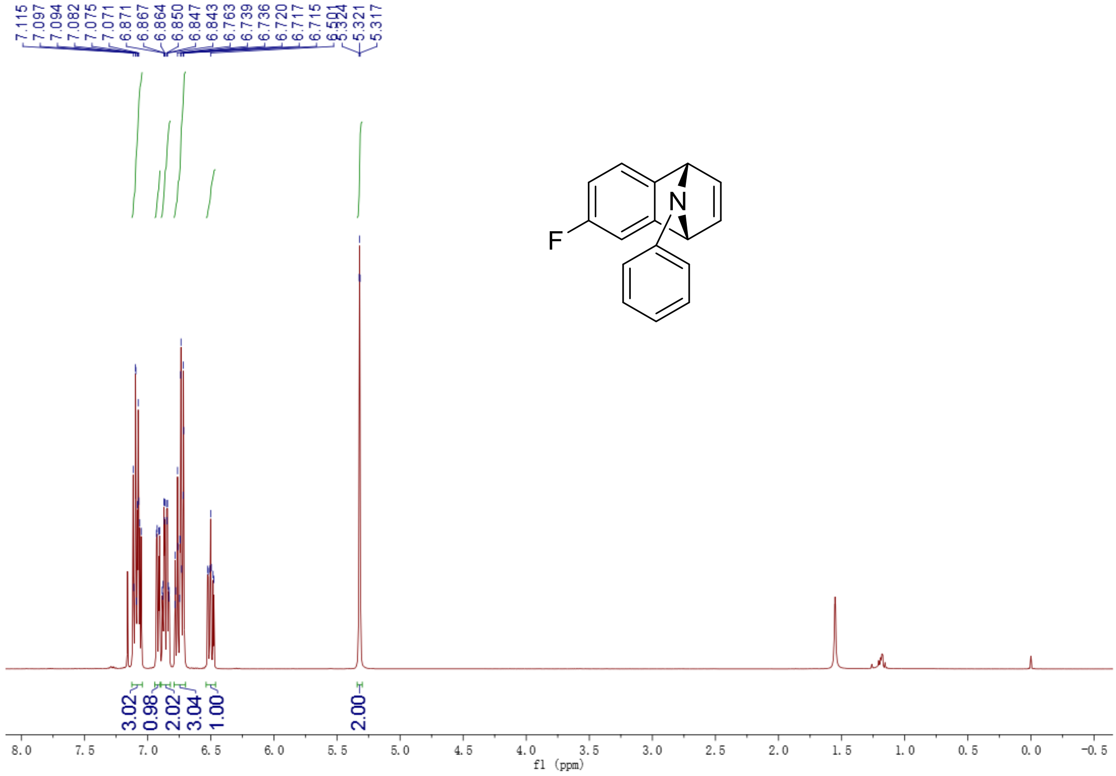
7.115
7.097
7.094
7.082
7.075
7.071
6.871
6.867
6.864
6.850
6.847
6.843
6.763
6.739
6.736
6.720
6.717
6.715
6.501
5.324
5.321
5.317
F
N
3.02
0.98
2.02
3.04
1.00
2.00
8.0 7.5 7.0 6.5 6.0 5.5 5.0 4.5 4.0 3.5 3.0 2.5 2.0 1.5 1.0 0.5 0.0 -0.5
f1 (ppm)

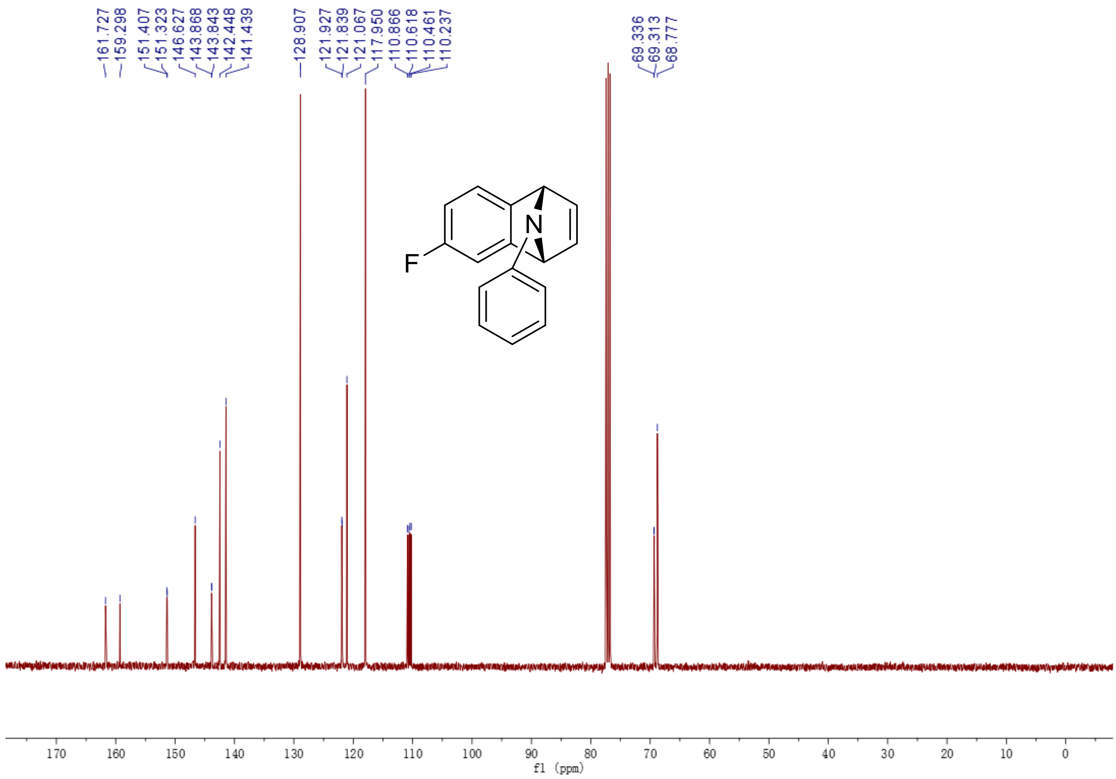
161.727
159.298
151.407
151.323
146.627
143.868
143.843
142.448
141.439
128.907
121.927
121.839
121.067
117.950
110.866
110.618
110.461
110.237
69.336
69.313
68.777
F
N
f1 (ppm)

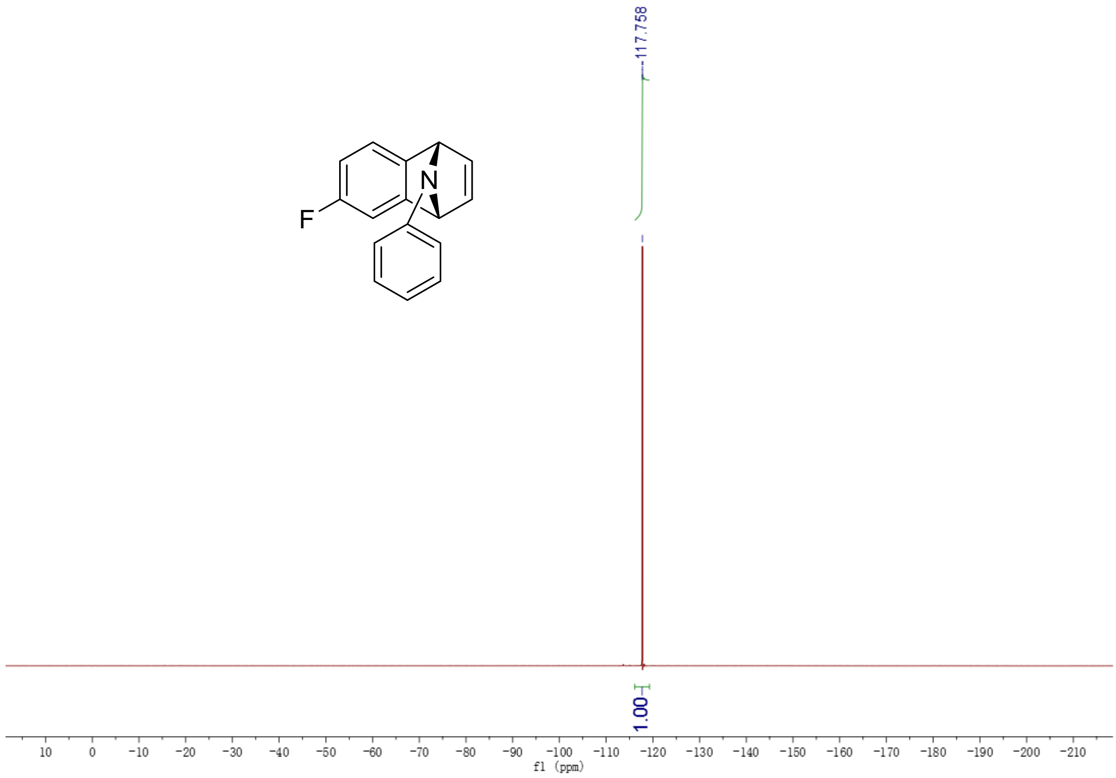
-117.758
F
N
1.00
f1 (ppm)

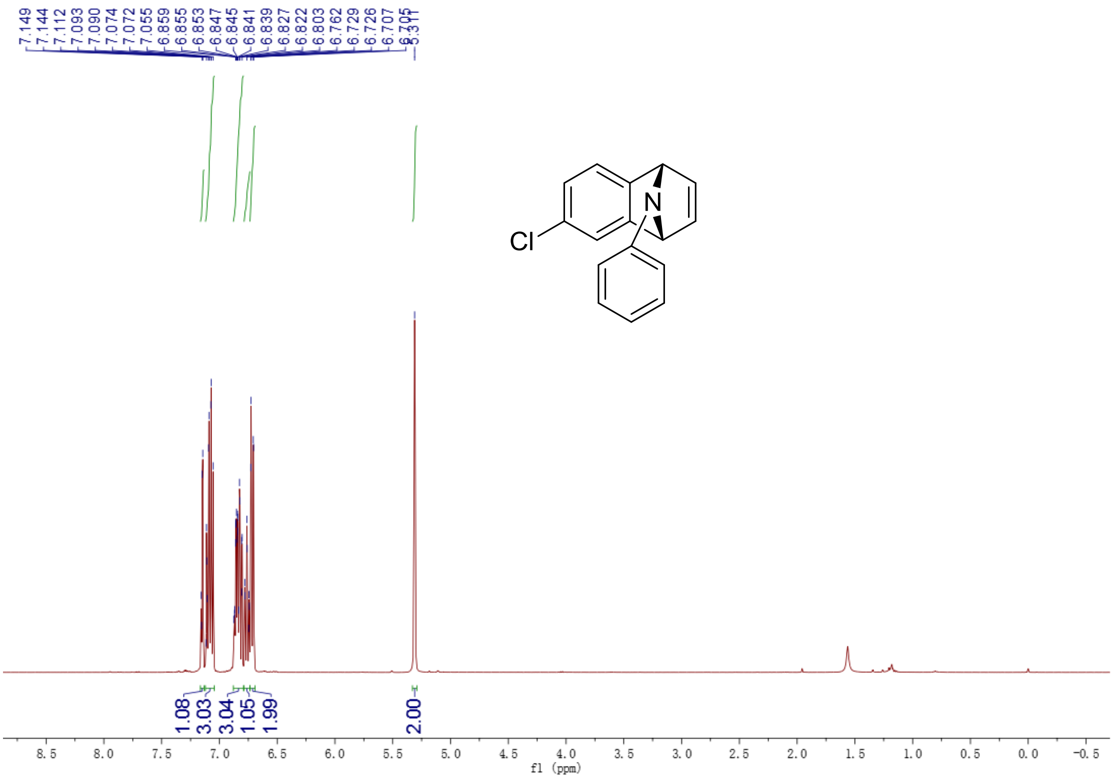
7.149
7.144
7.112
7.093
7.090
7.074
7.072
7.055
6.859
6.855
6.853
6.847
6.845
6.841
6.839
6.827
6.822
6.803
6.762
6.729
6.726
6.707
6.705
5.311
N
Cl
1.08
3.03
3.04
1.05
1.99
2.00
8.5 8.0 7.5 7.0 6.5 6.0 5.5 5.0 4.5 4.0 3.5 3.0 2.5 2.0 1.5 1.0 0.5 0.0 -0.5
f1 (ppm)

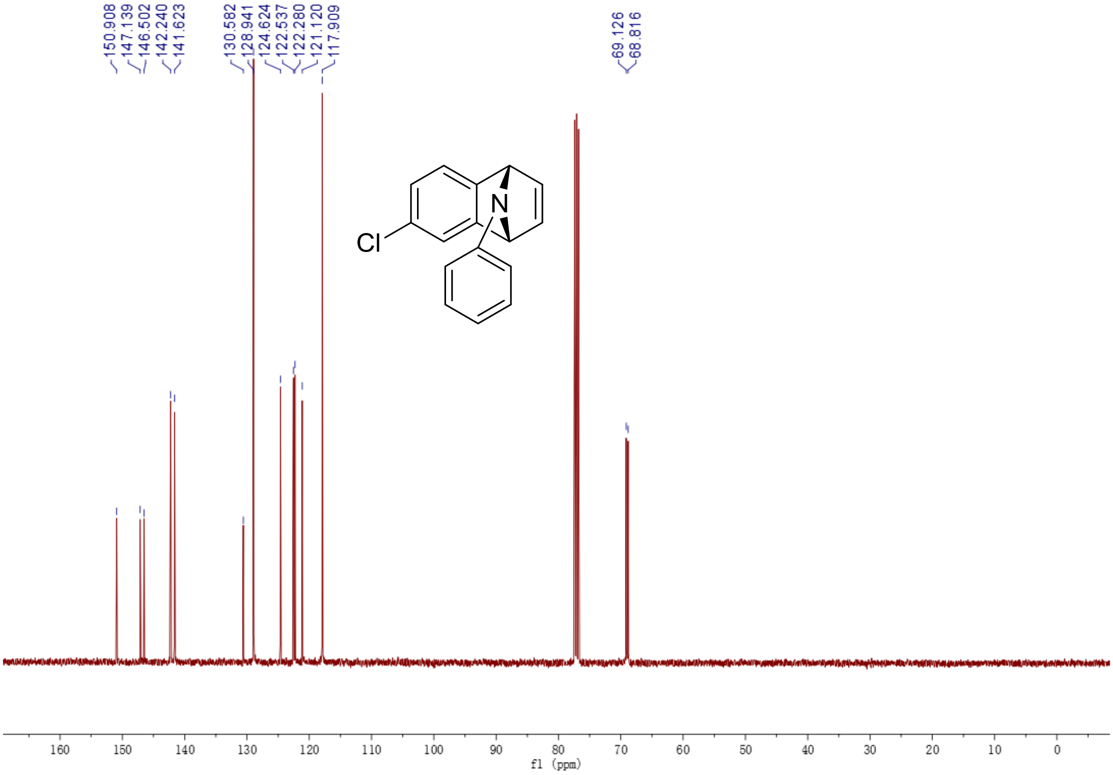
150.908
147.139
146.502
142.240
141.623
130.582
128.941
124.624
122.537
122.280
121.120
117.909
69.126
68.816
N
Cl
160 150 140 130 120 110 100 90 80 70 60 50 40 30 20 10 0
f1 (ppm)

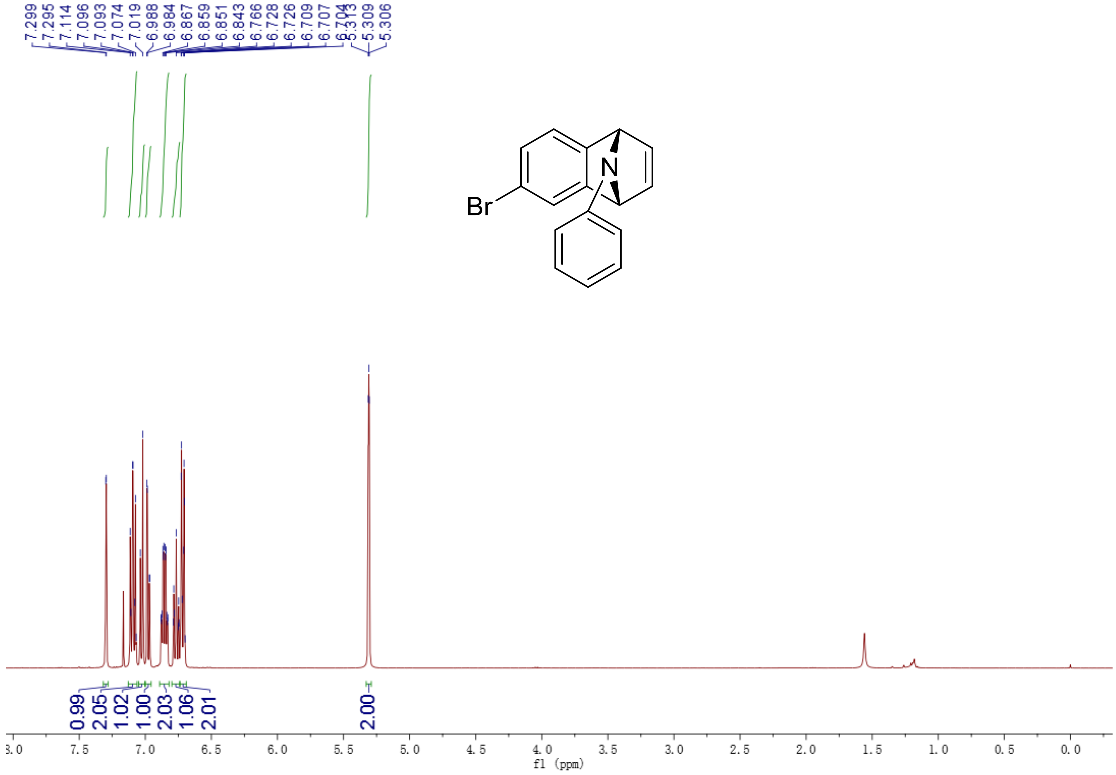
7.299
7.295
7.114
7.096
7.093
7.074
7.019
6.988
6.984
6.867
6.859
6.851
6.843
6.766
6.728
6.726
6.709
6.707
6.704
5.313
5.309
5.306
Br
N
0.99
2.05
1.02
1.00
2.03
1.06
2.01
2.00
f1 (ppm)

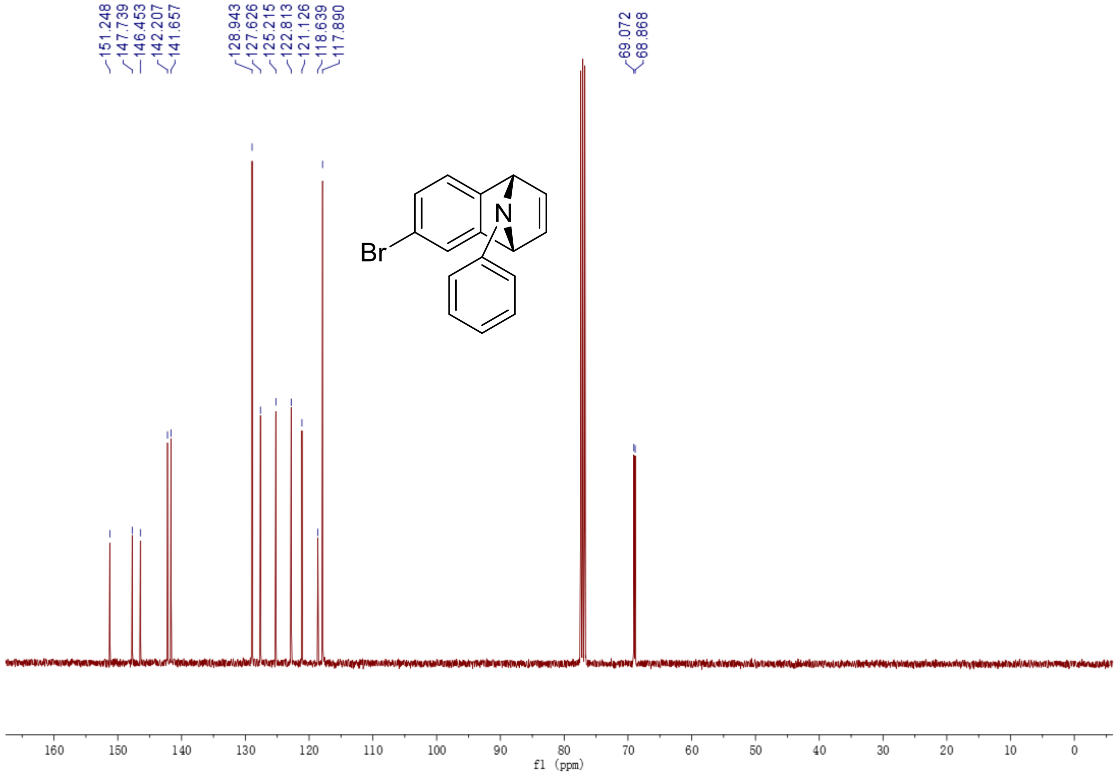
151.248
147.739
146.453
142.207
141.657
128.943
127.626
125.215
122.813
121.126
118.639
117.890
69.072
68.868
Br
N
f1 (ppm)

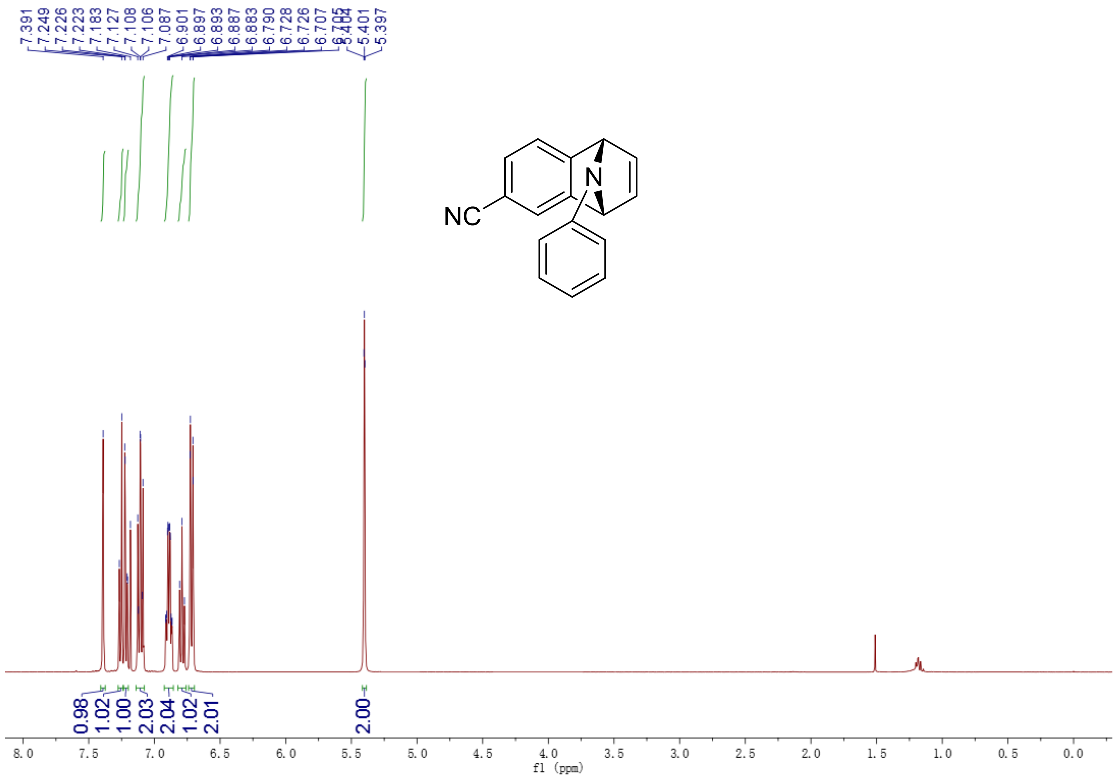

7.391
7.249
7.226
7.223
7.183
7.127
7.108
7.106
7.087
6.901
6.897
6.893
6.887
6.883
6.790
6.728
6.726
6.707
5.401
5.397
NC
N
0.98
1.02
1.00
2.03
2.04
1.02
2.01
2.00
f1 (ppm)

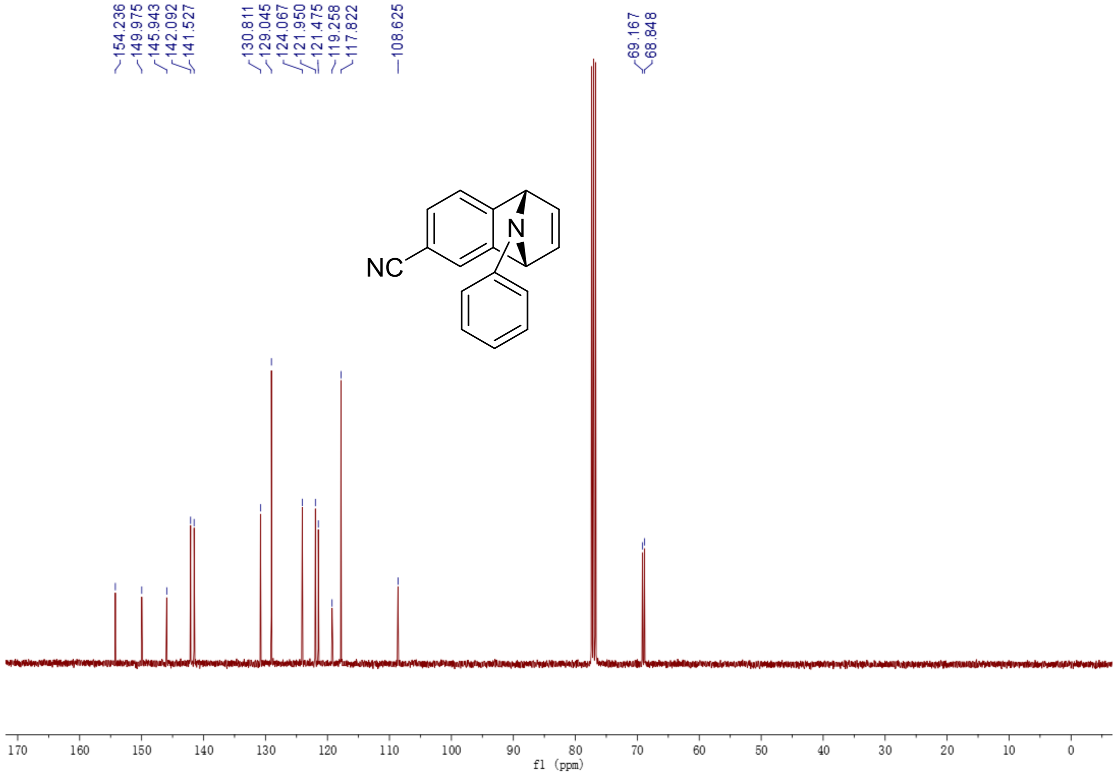

154.236
149.975
145.943
142.092
141.527
130.811
129.045
124.067
121.950
121.475
119.258
117.822
108.625
69.167
68.848
NC
N
f1 (ppm)

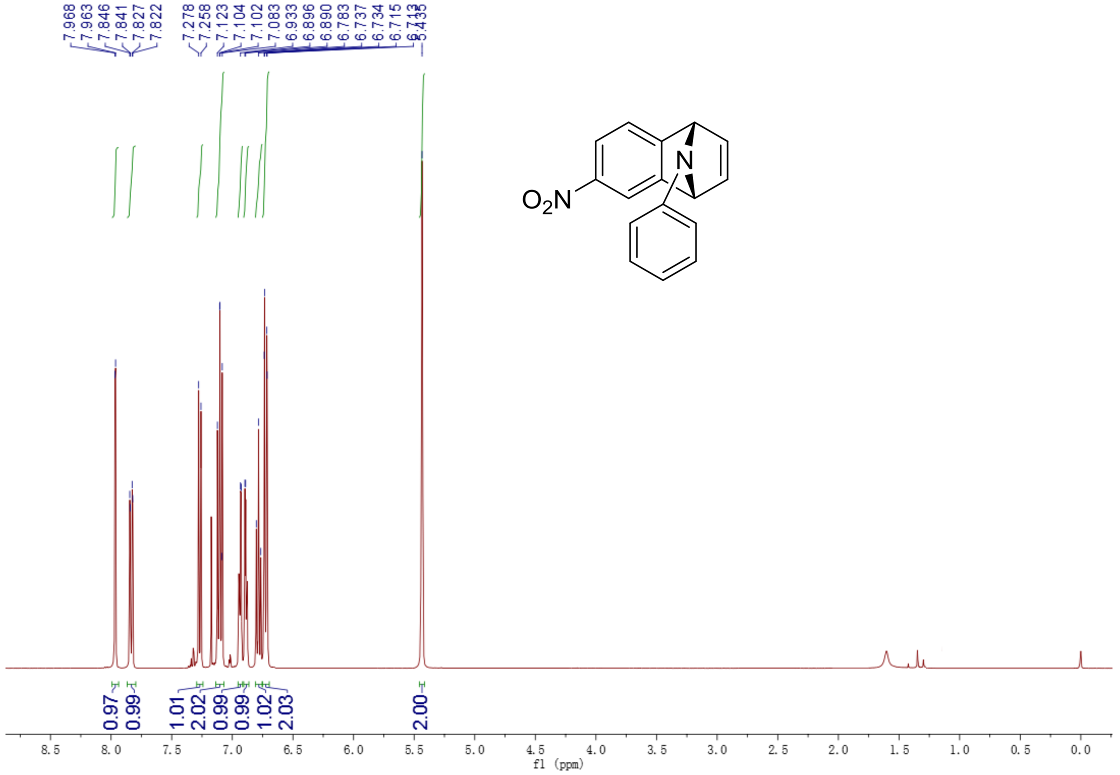

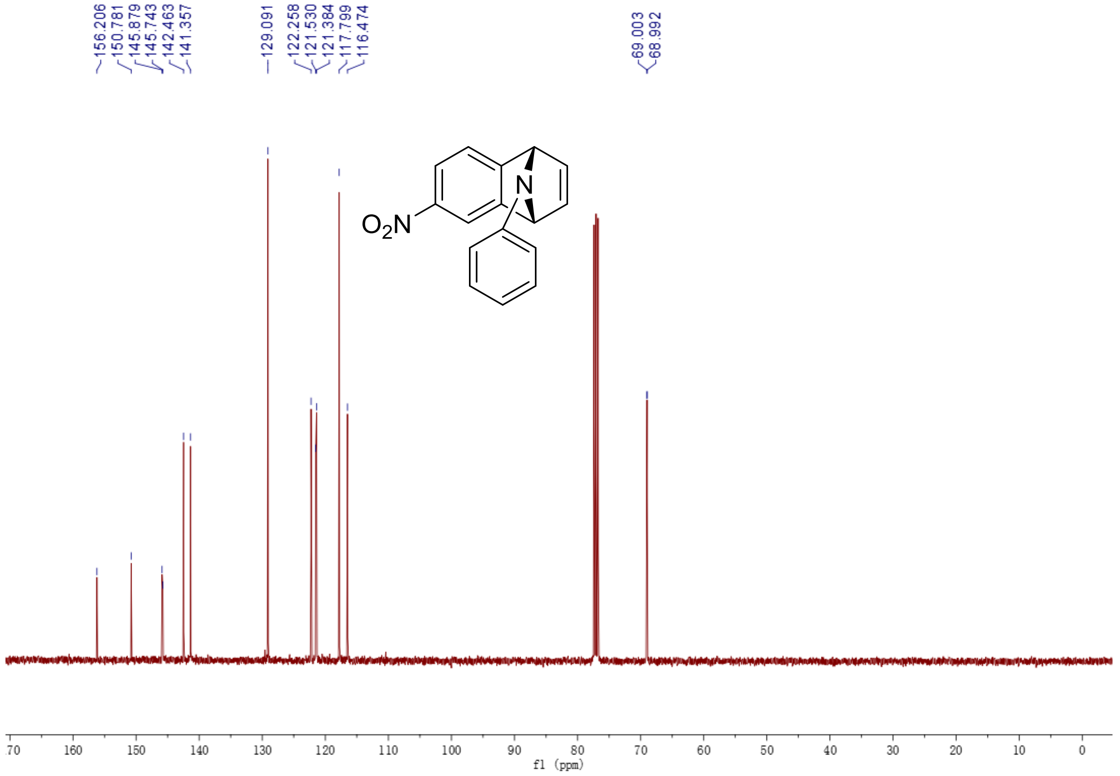

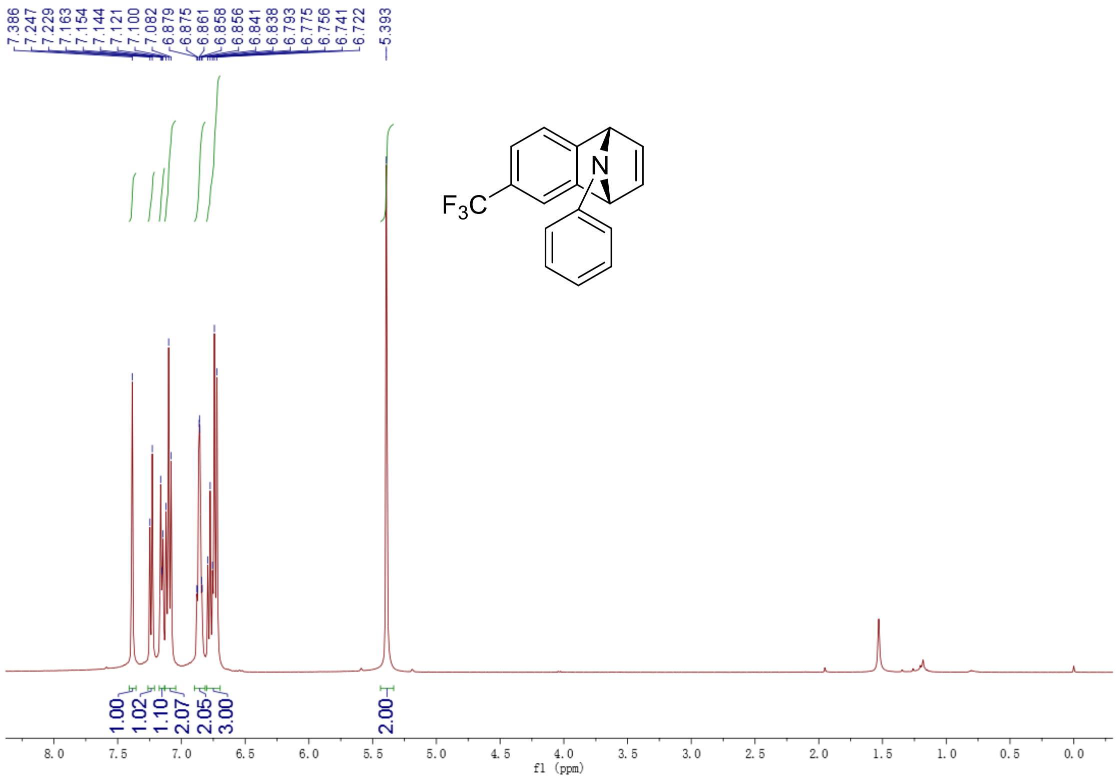
7.386
7.247
7.229
7.163
7.154
7.144
7.121
7.100
7.082
6.879
6.875
6.861
6.858
6.856
6.841
6.838
6.793
6.775
6.756
6.741
6.722
5.393
F3C
N
1.00
1.02
1.10
2.07
2.05
3.00
2.00
8.0
7.5
7.0
6.5
6.0
5.5
5.0
4.5
4.0
3.5
3.0
2.5
2.0
1.5
1.0
0.5
0.0
f1 (ppm)

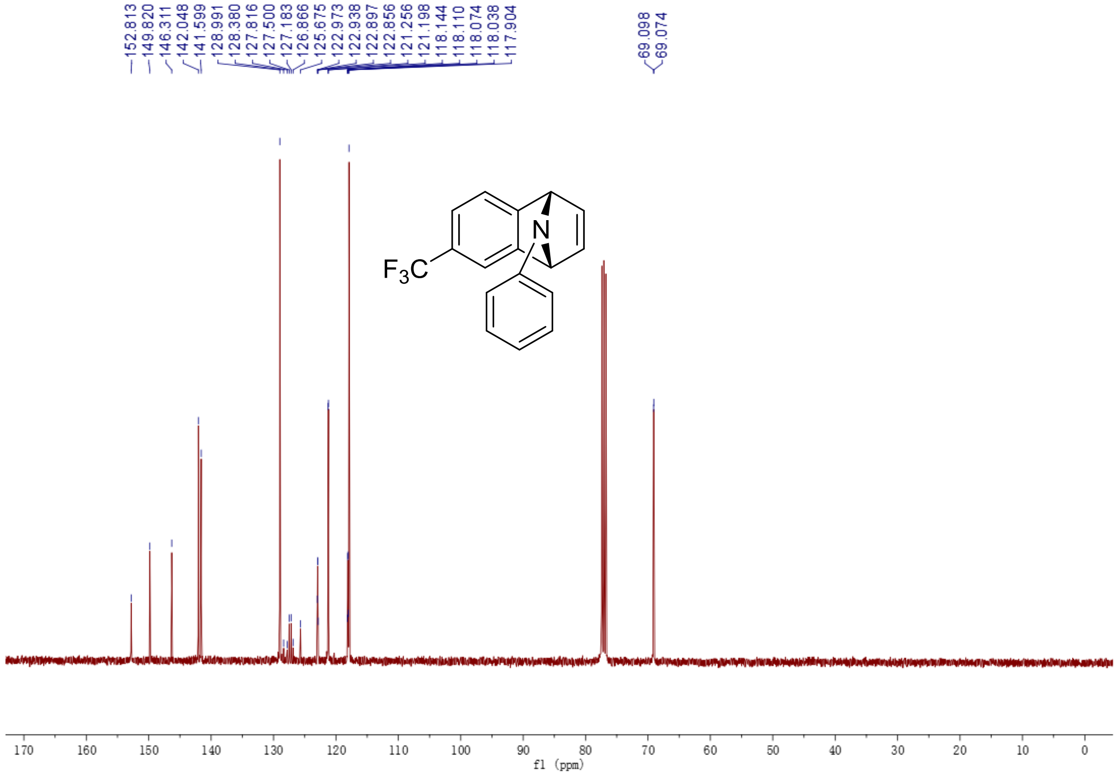
152.813
149.820
146.311
142.048
141.599
128.991
128.380
127.816
127.500
127.183
126.866
125.675
122.973
122.938
122.897
122.856
121.256
121.198
118.144
118.110
118.074
118.038
117.904
69.098
69.074
F3C
N
170
160
150
140
130
120
110
100
90
80
70
60
50
40
30
20
10
0
f1 (ppm)

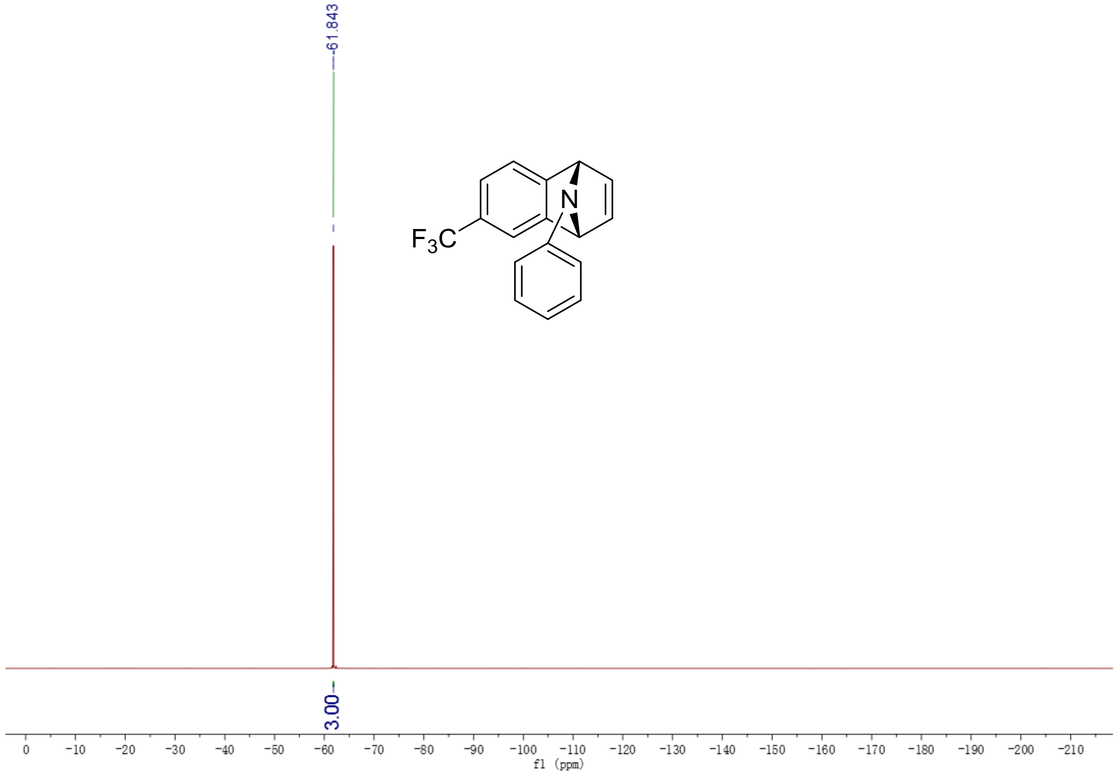
-61.843
F3C
N
3.00
0 -10 -20 -30 -40 -50 -60 -70 -80 -90 -100 -110 -120 -130 -140 -150 -160 -170 -180 -190 -200 -210
f1 (ppm)

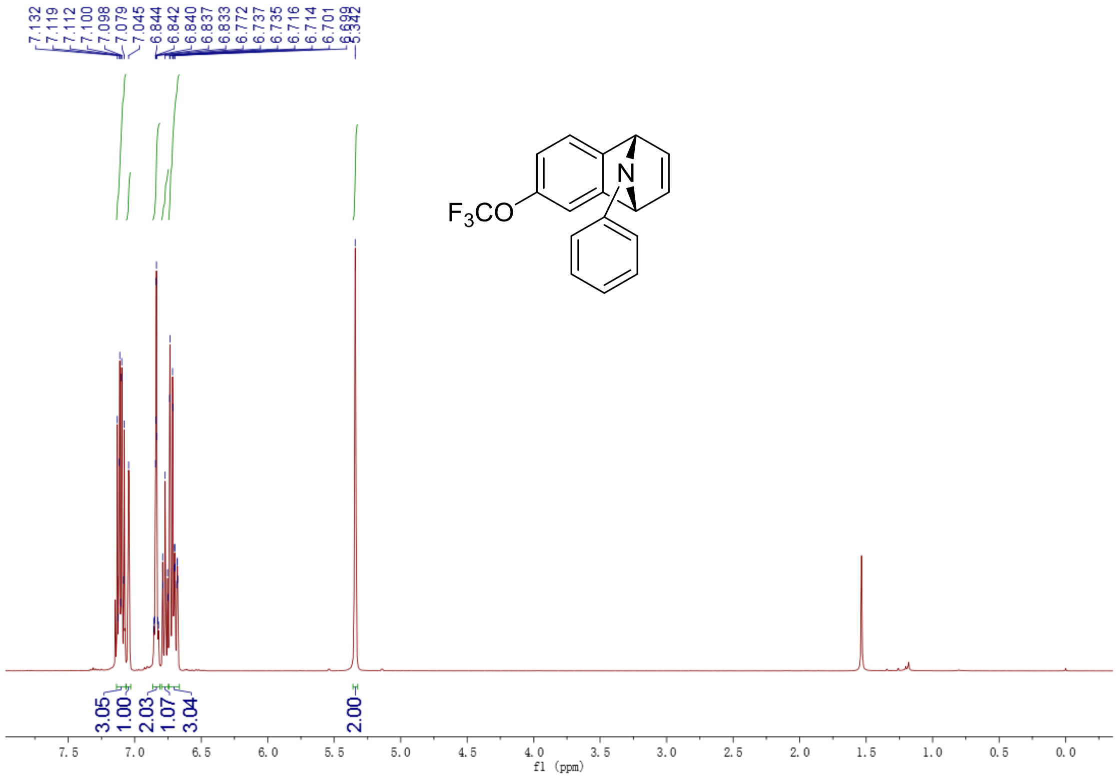
7.132
7.119
7.112
7.100
7.098
7.079
7.045
6.844
6.842
6.840
6.837
6.833
6.772
6.737
6.735
6.716
6.714
6.701
6.699
5.342
F3CO
N
3.05
1.00
2.03
1.07
3.04
2.00
7.5 7.0 6.5 6.0 5.5 5.0 4.5 4.0 3.5 3.0 2.5 2.0 1.5 1.0 0.5 0.0
f1 (ppm)

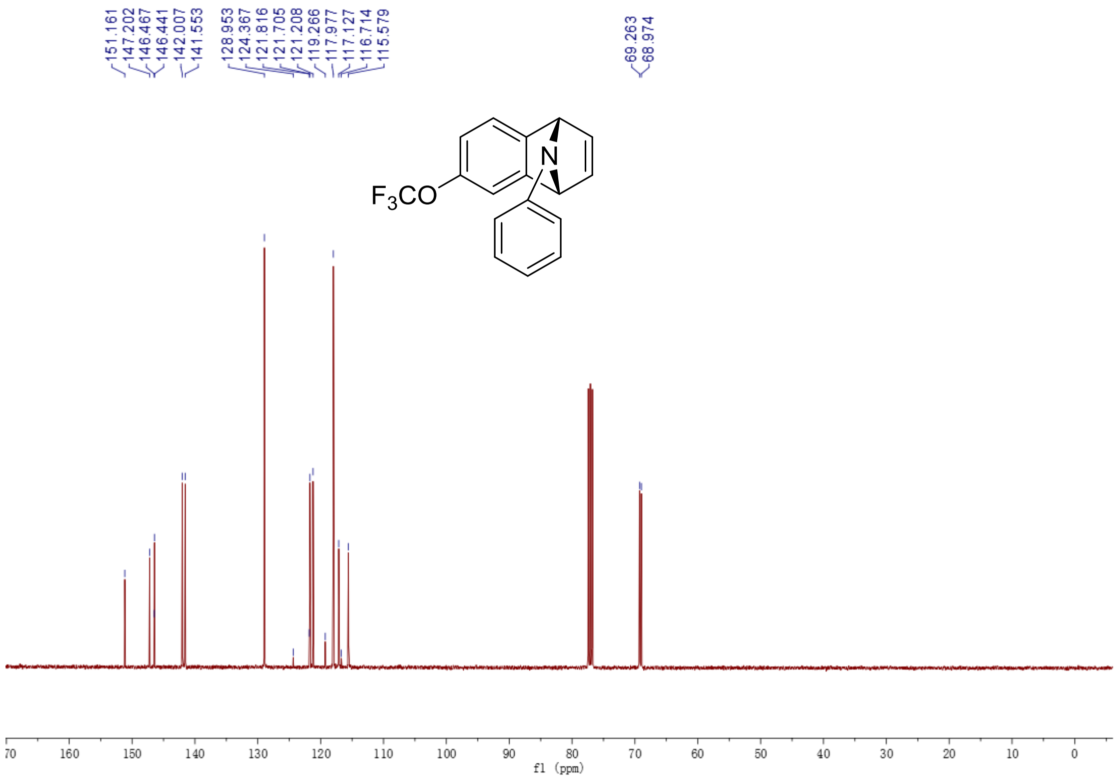

151.161
147.202
146.467
146.441
142.007
141.553
128.953
124.367
121.816
121.705
121.208
119.266
117.977
117.127
116.714
115.579
69.263
68.974
F3CO
N
170
160
150
140
130
120
110
100
90
80
70
60
50
40
30
20
10
0
f1 (ppm)

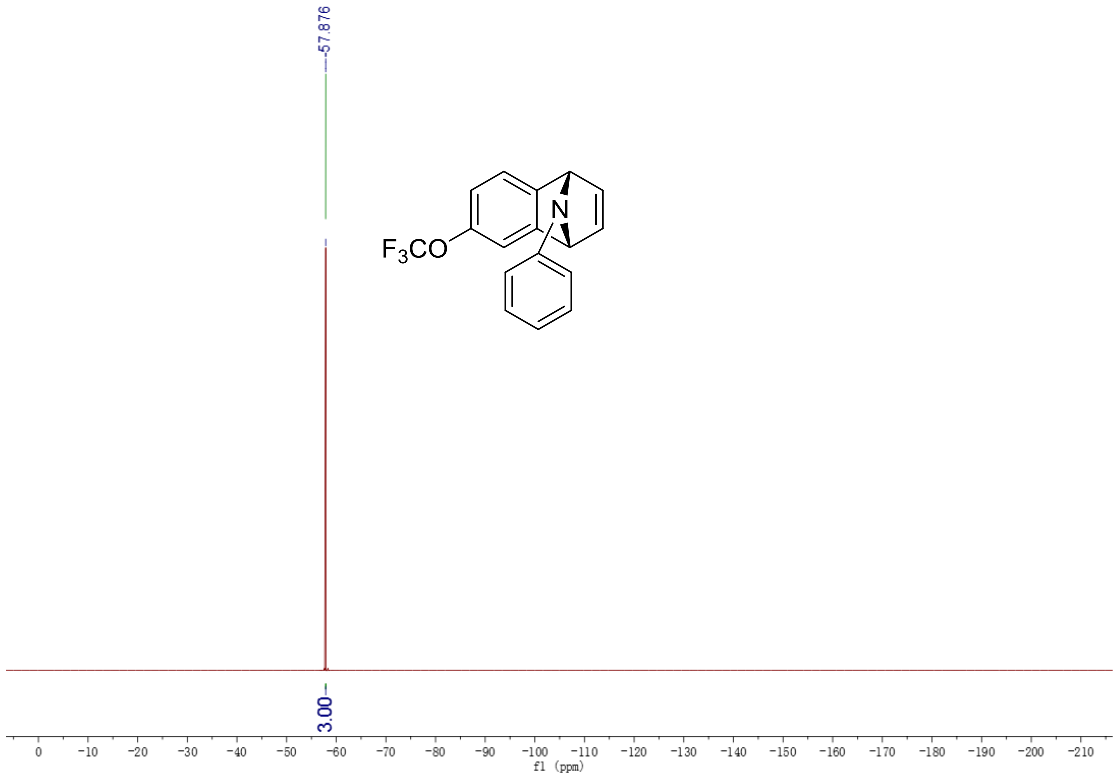

-57.876
F3CO
N
3.00
0
-10
-20
-30
-40
-50
-60
-70
-80
-90
-100
-110
-120
-130
-140
-150
-160
-170
-180
-190
-200
-210
f1 (ppm)

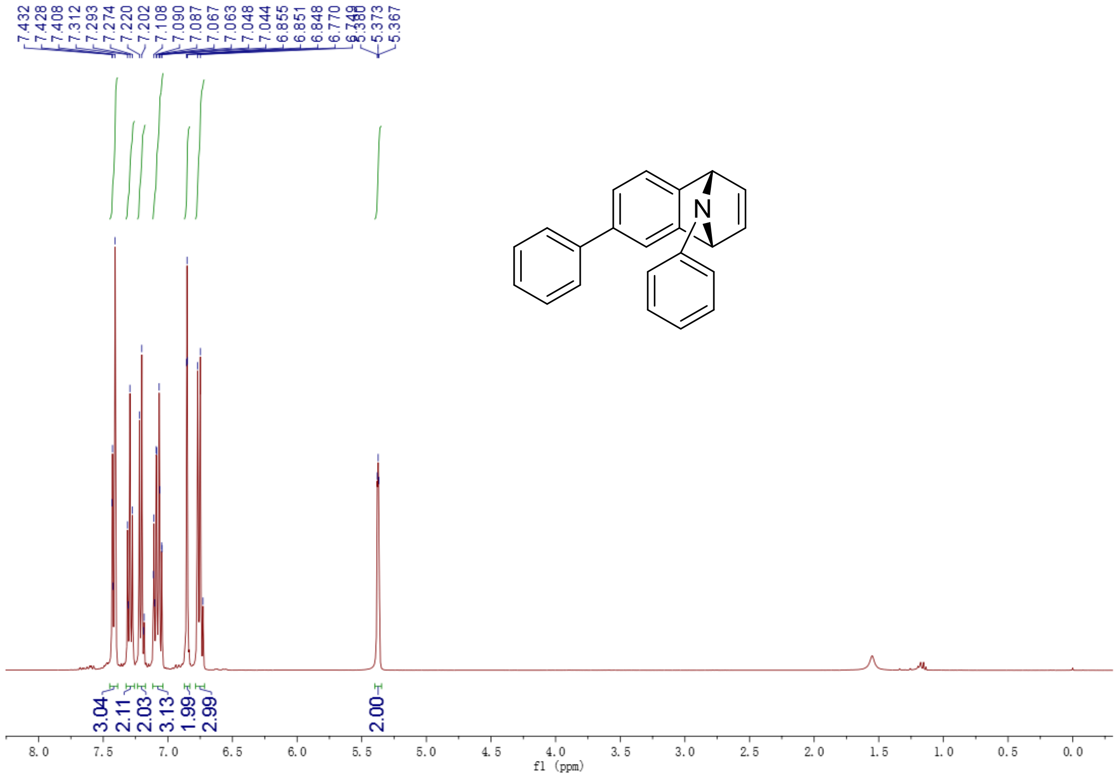
7.432
7.428
7.408
7.312
7.293
7.274
7.220
7.202
7.108
7.090
7.087
7.067
7.063
7.048
7.044
6.855
6.851
6.848
6.770
6.749
5.380
5.373
5.367
3.04
2.11
2.03
3.13
1.99
2.99
2.00
N
f1 (ppm)

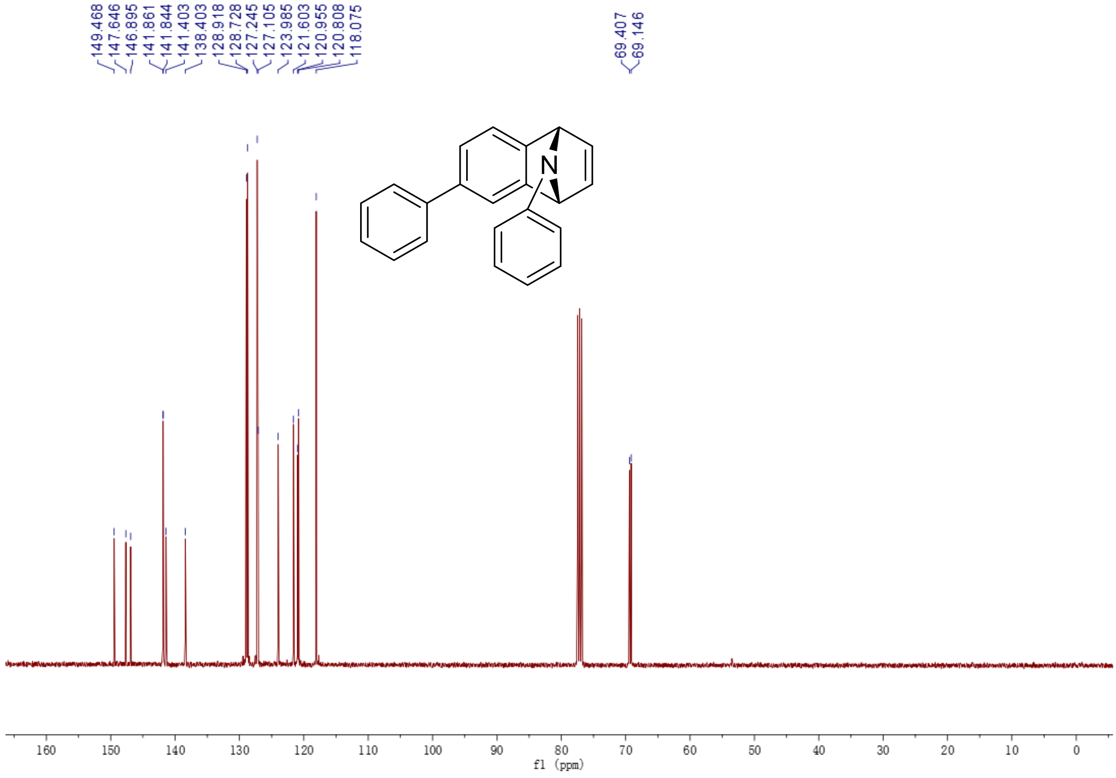
149.468
147.646
146.895
141.861
141.844
141.403
138.403
128.918
128.728
127.245
127.105
123.985
121.603
120.955
120.808
118.075
69.407
69.146
N
f1 (ppm)

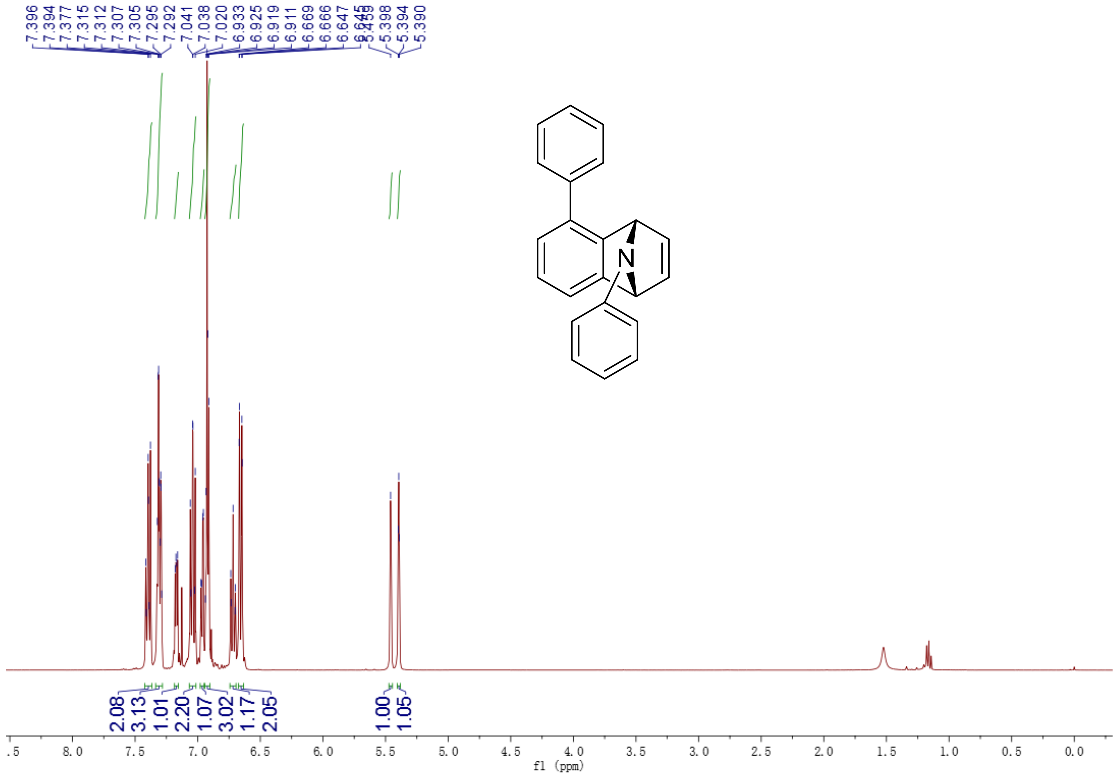
N
f1 (ppm)

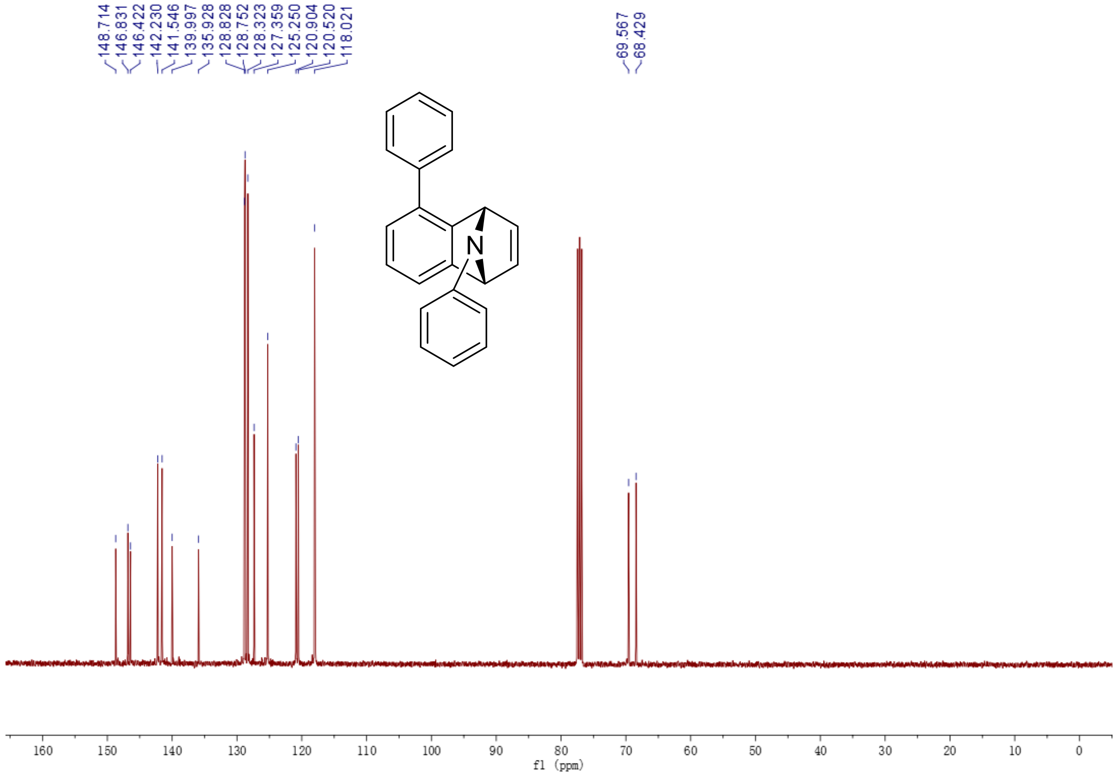
N
f1 (ppm)

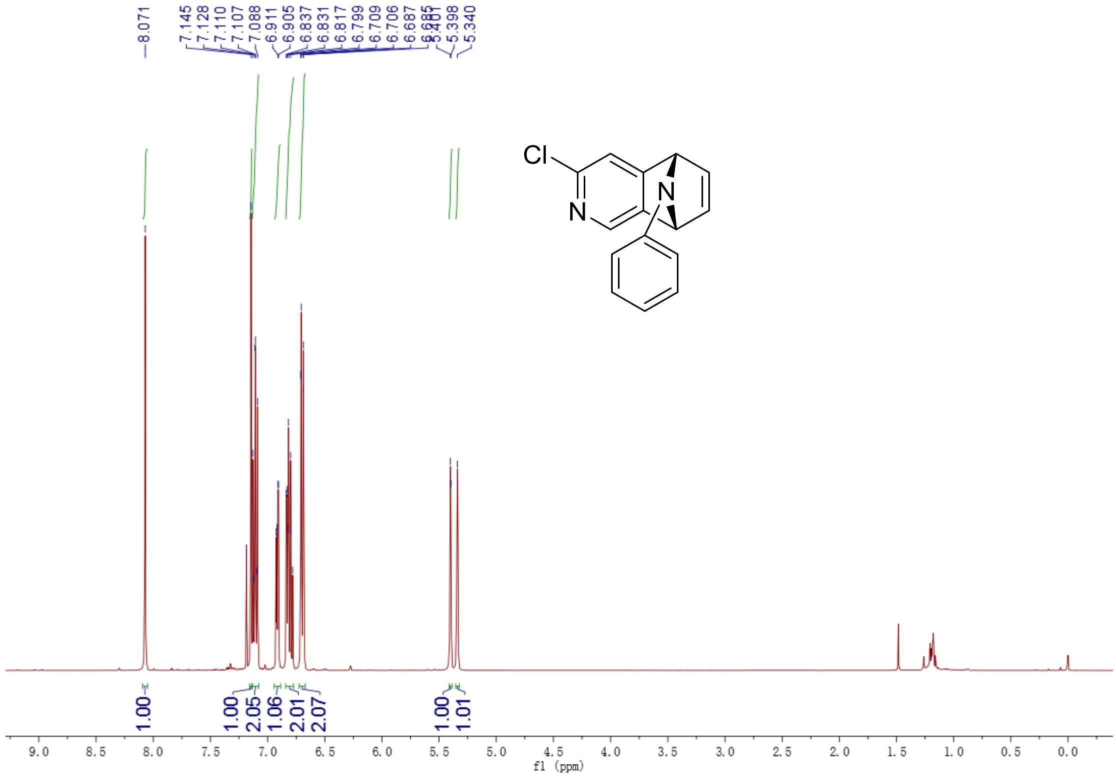
8.071
7.145
7.128
7.110
7.107
7.088
6.911
6.905
6.837
6.831
6.817
6.799
6.709
6.706
6.687
5.398
5.340
Cl
N
N
1.00
1.00
2.05
1.06
2.01
2.07
1.00
1.01
f1 (ppm)

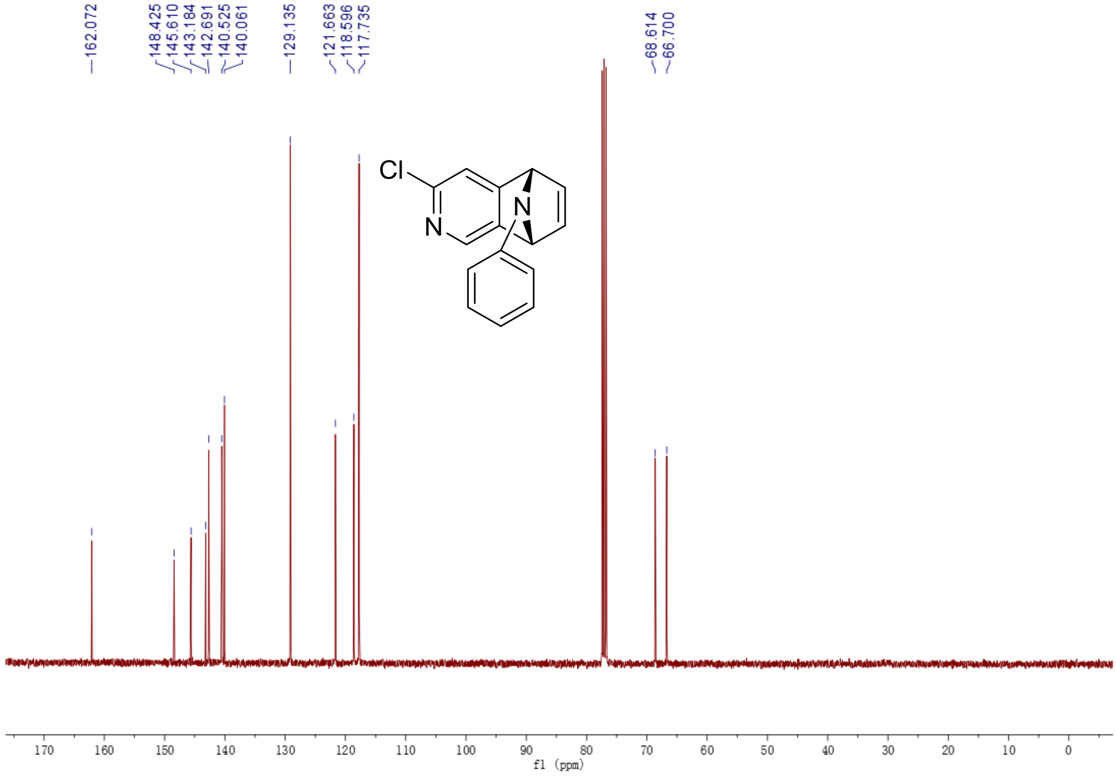
162.072
148.425
145.610
143.184
142.691
140.525
140.061
129.135
121.663
118.596
117.735
68.614
66.700
Cl
N
N
f1 (ppm)

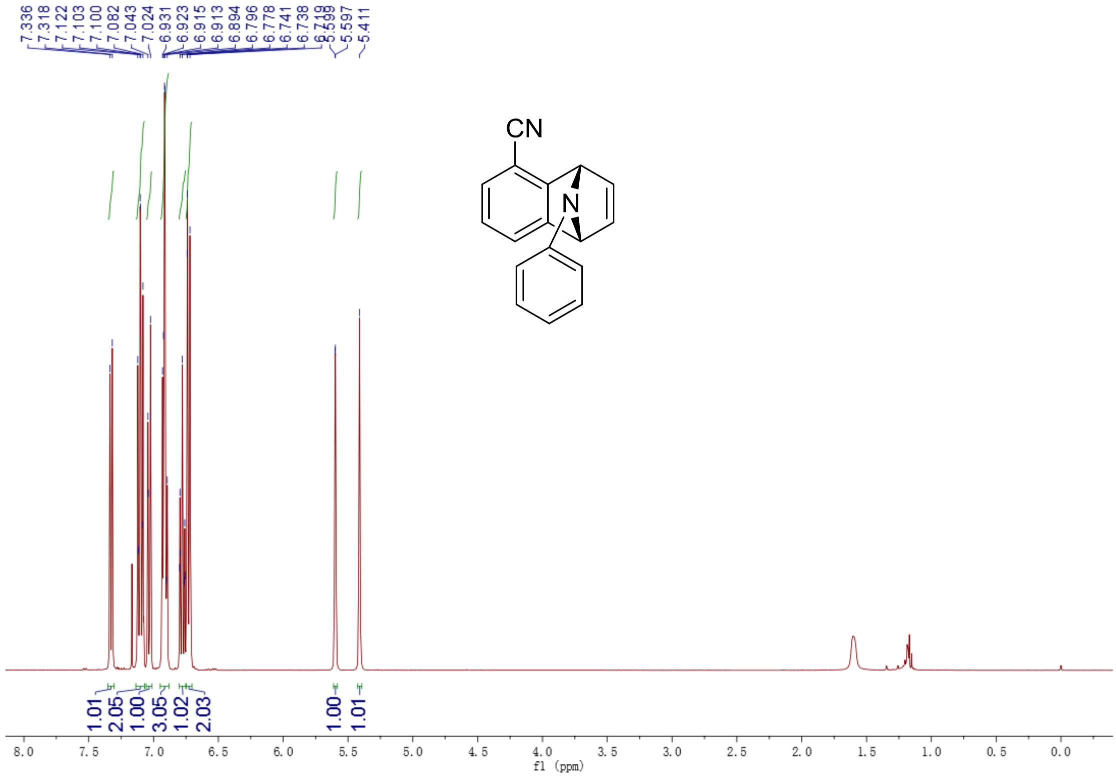

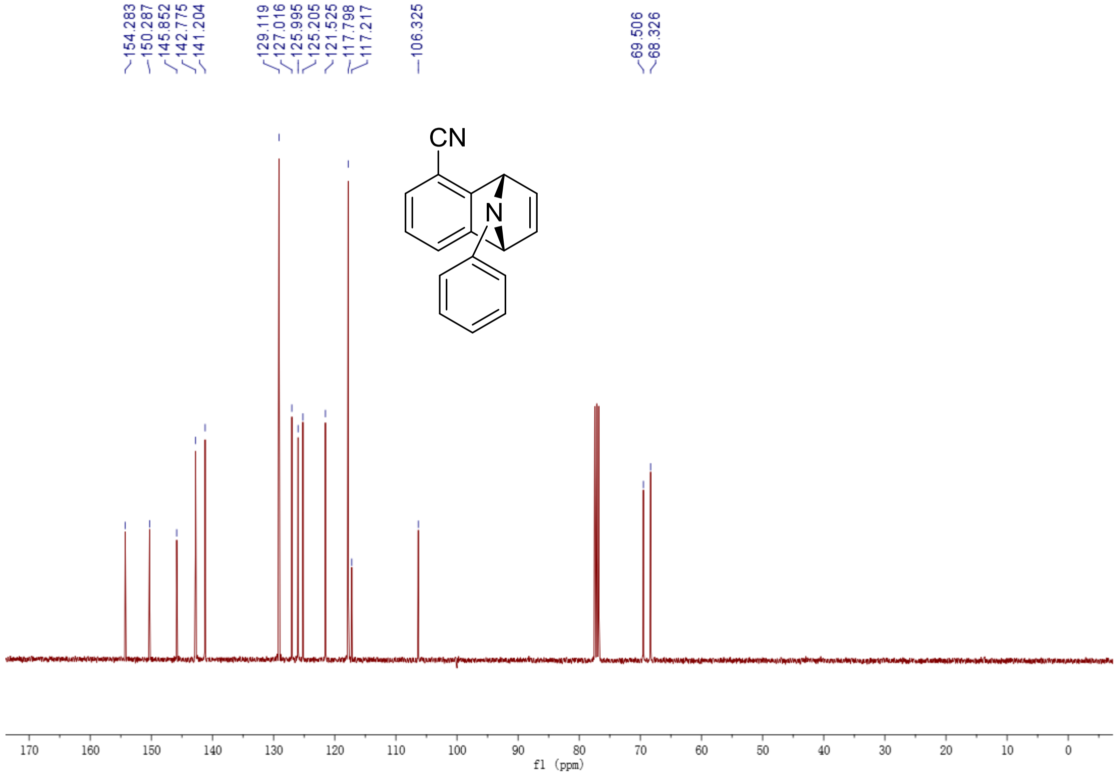

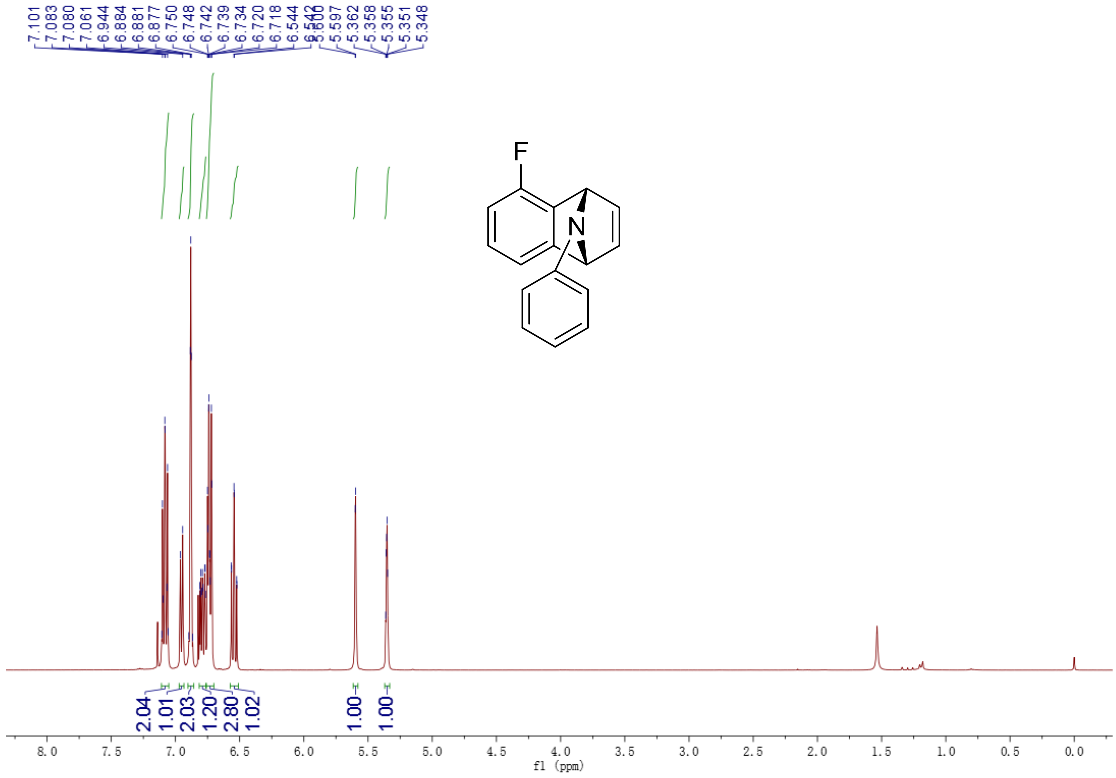

7.101
7.083
7.080
7.061
6.944
6.884
6.881
6.877
6.750
6.748
6.742
6.739
6.734
6.720
6.718
6.544
5.597
5.362
5.358
5.355
5.351
5.348
F
N
2.04
1.01
2.03
1.20
2.80
1.02
1.00
1.00
8.0 7.5 7.0 6.5 6.0 5.5 5.0 4.5 4.0 3.5 3.0 2.5 2.0 1.5 1.0 0.5 0.0
f1 (ppm)

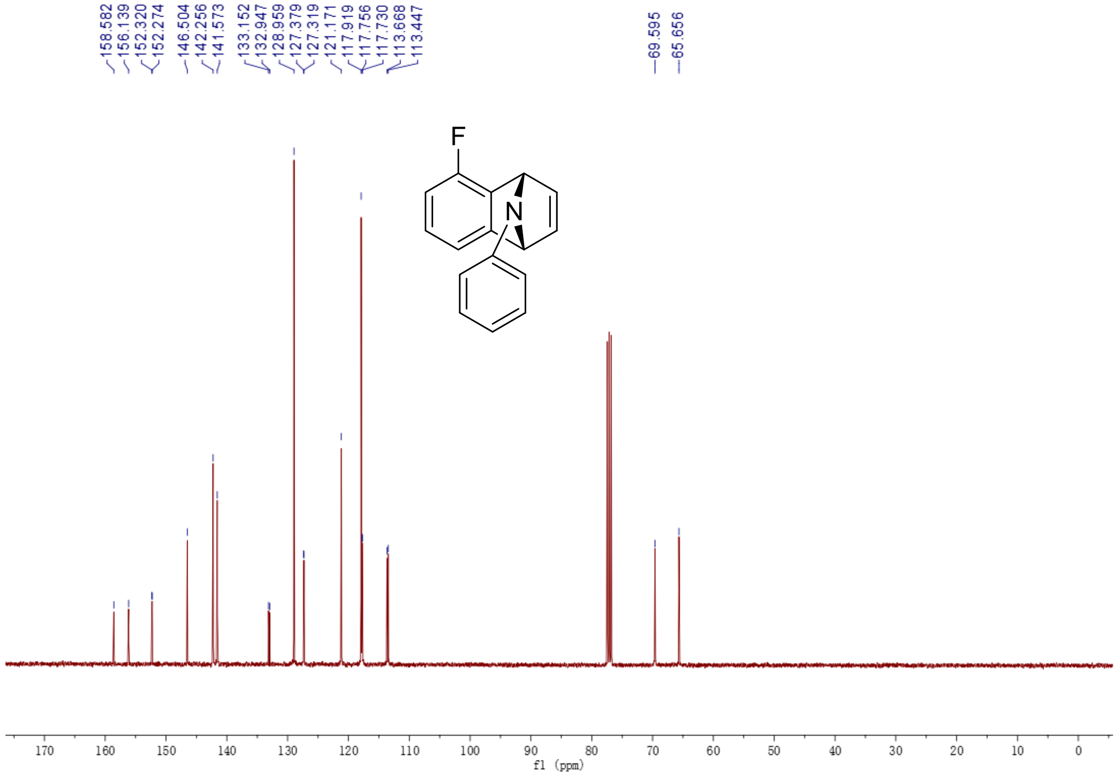

158.582
156.139
152.320
152.274
146.504
142.256
141.573
133.152
132.947
128.959
127.379
127.319
121.171
117.919
117.756
117.730
113.668
113.447
69.595
65.656
F
N
170 160 150 140 130 120 110 100 90 80 70 60 50 40 30 20 10 0
f1 (ppm)

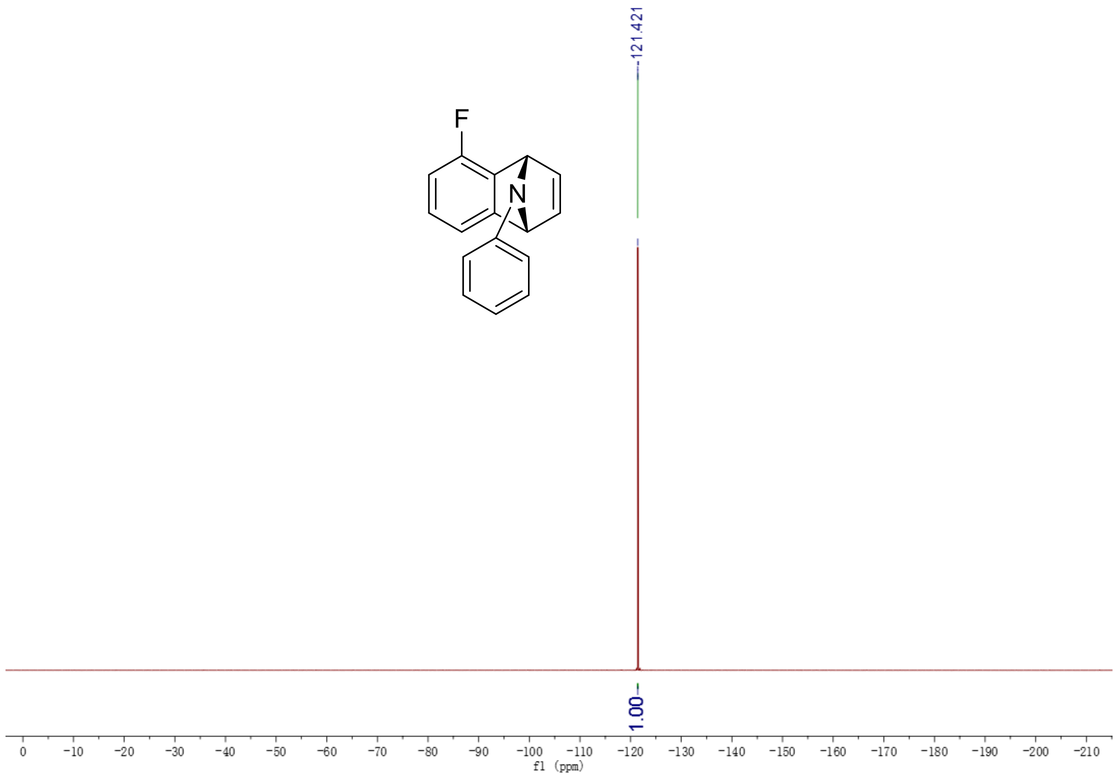

-121.421
F
N
1.00
0
-10
-20
-30
-40
-50
-60
-70
-80
-90
-100
-110
-120
-130
-140
-150
-160
-170
-180
-190
-200
-210
f1 (ppm)

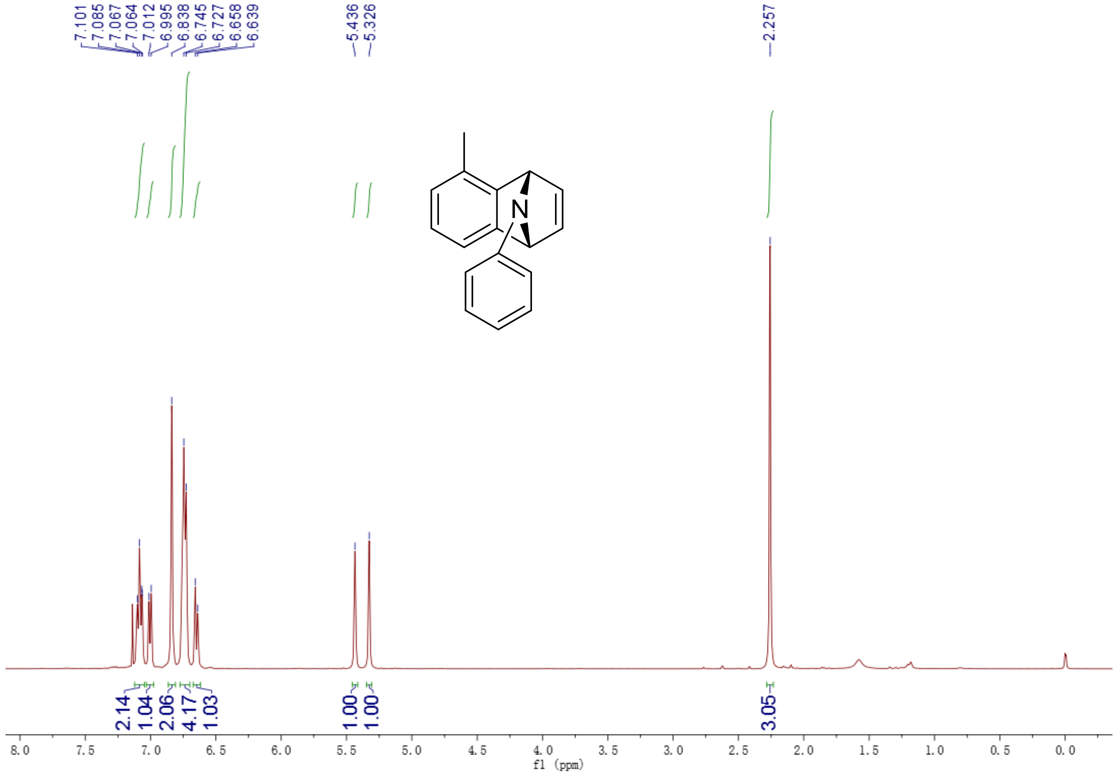

7.101
7.085
7.067
7.064
7.012
6.995
6.838
6.745
6.727
6.658
6.639
5.436
5.326
2.257
N
2.14
1.04
2.06
4.17
1.03
1.00
1.00
3.05
8.0
7.5
7.0
6.5
6.0
5.5
5.0
4.5
4.0
3.5
3.0
2.5
2.0
1.5
1.0
0.5
0.0
f1 (ppm)

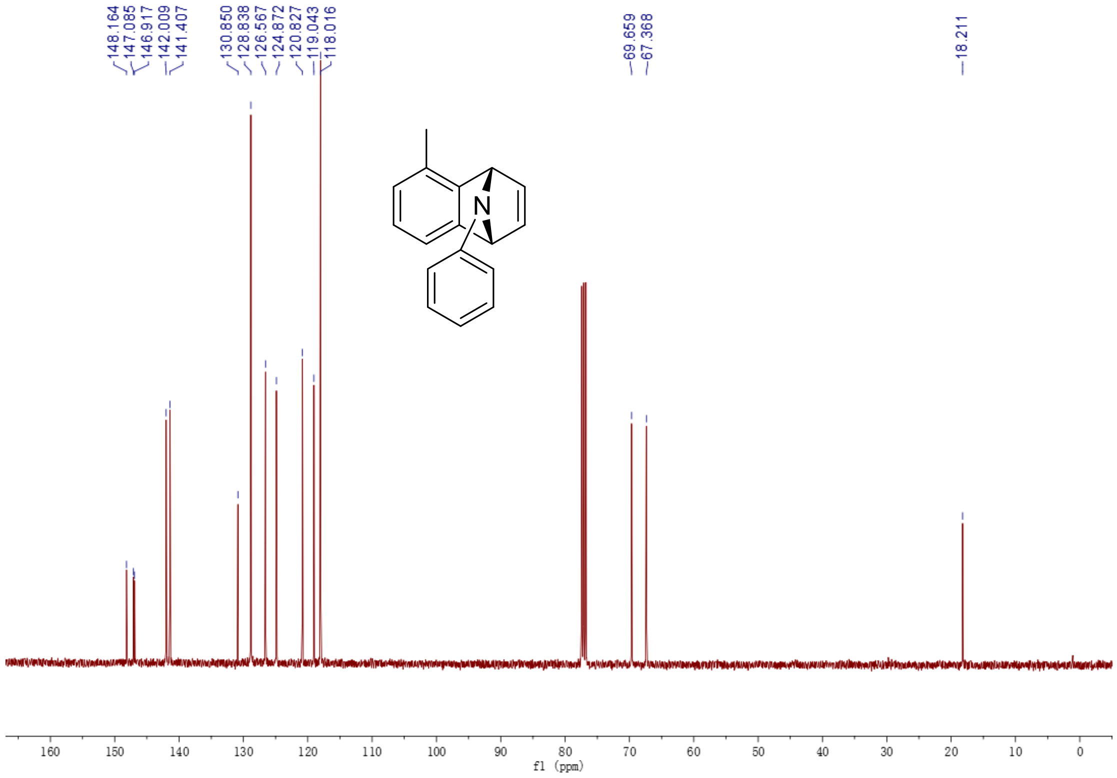
148.164
147.085
146.917
142.009
141.407
130.850
128.838
126.567
124.872
120.827
119.043
118.016
69.659
67.368
18.211
N
160 150 140 130 120 110 100 90 80 70 60 50 40 30 20 10 0
f1 (ppm)

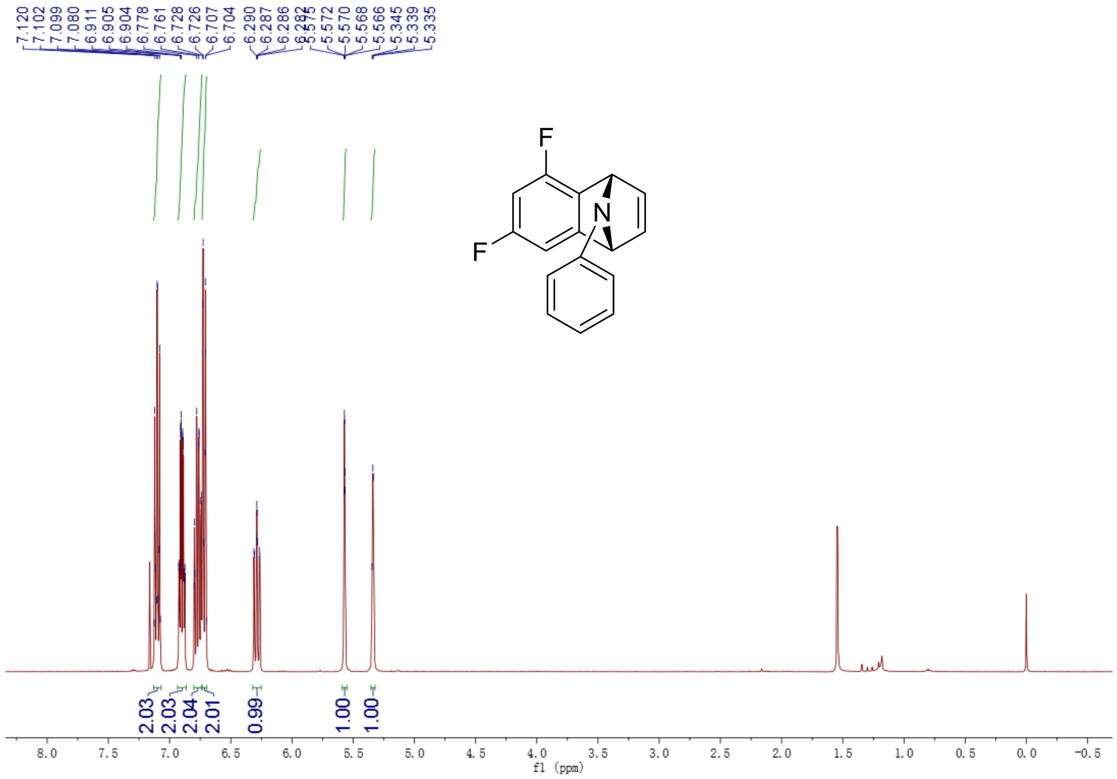
7.120
7.102
7.099
7.080
6.911
6.905
6.904
6.778
6.761
6.728
6.726
6.707
6.704
6.290
6.287
6.286
5.572
5.570
5.568
5.566
5.345
5.339
5.335
F
F
N
2.03
2.03
2.04
2.01
0.99
1.00
1.00
8.0 7.5 7.0 6.5 6.0 5.5 5.0 4.5 4.0 3.5 3.0 2.5 2.0 1.5 1.0 0.5 0.0 -0.5
f1 (ppm)

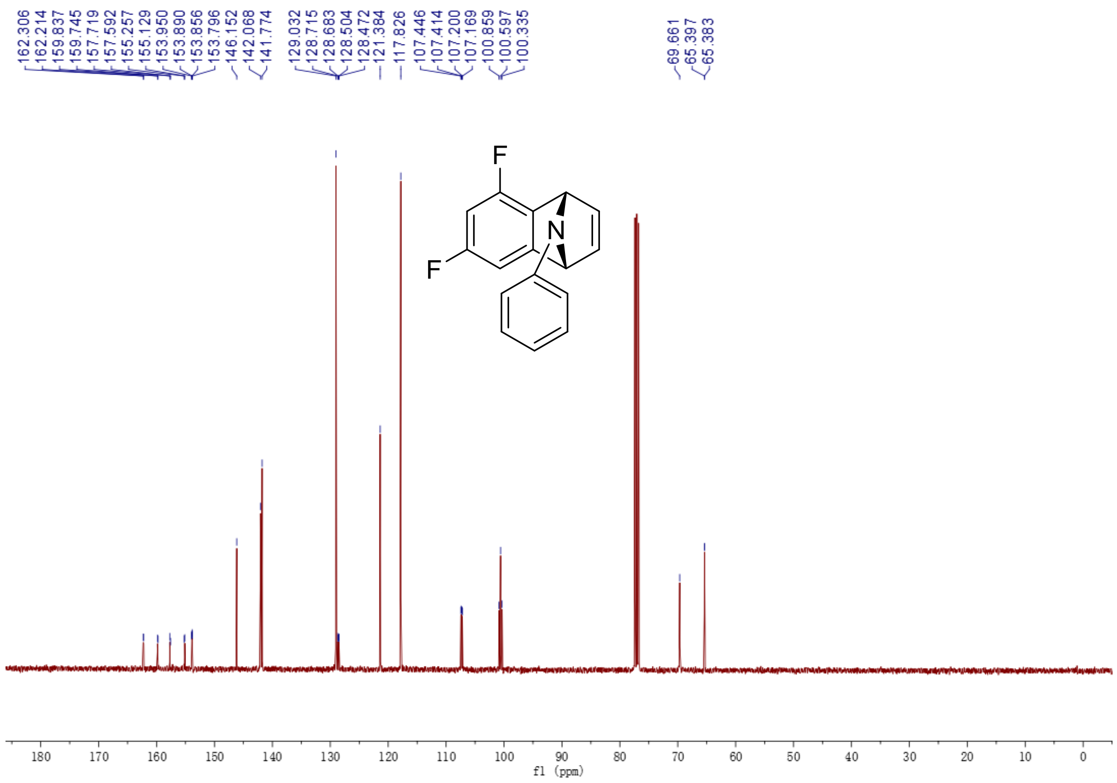
162.306
162.214
159.837
159.745
157.719
157.592
155.257
155.129
153.950
153.890
153.856
153.796
146.152
142.068
141.774
129.032
128.715
128.683
128.504
128.472
121.384
117.826
107.446
107.414
107.200
107.169
100.859
100.597
100.335
69.661
65.397
65.383
F
F
N
180 170 160 150 140 130 120 110 100 90 80 70 60 50 40 30 20 10 0
f1 (ppm)

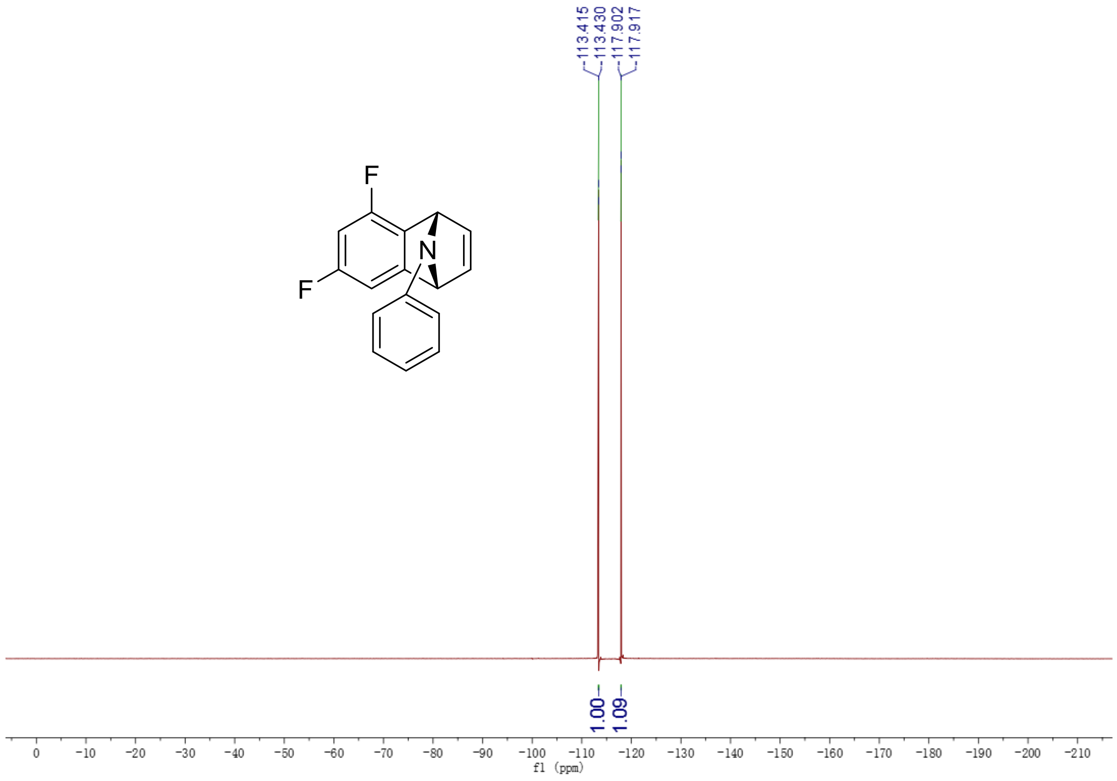
-113.415
-113.430
-117.902
-117.917
F
F
N
1.00
1.09
0 −10 −20 −30 −40 −50 −60 −70 −80 −90 −100 −110 −120 −130 −140 −150 −160 −170 −180 −190 −200 −210
f1 (ppm)

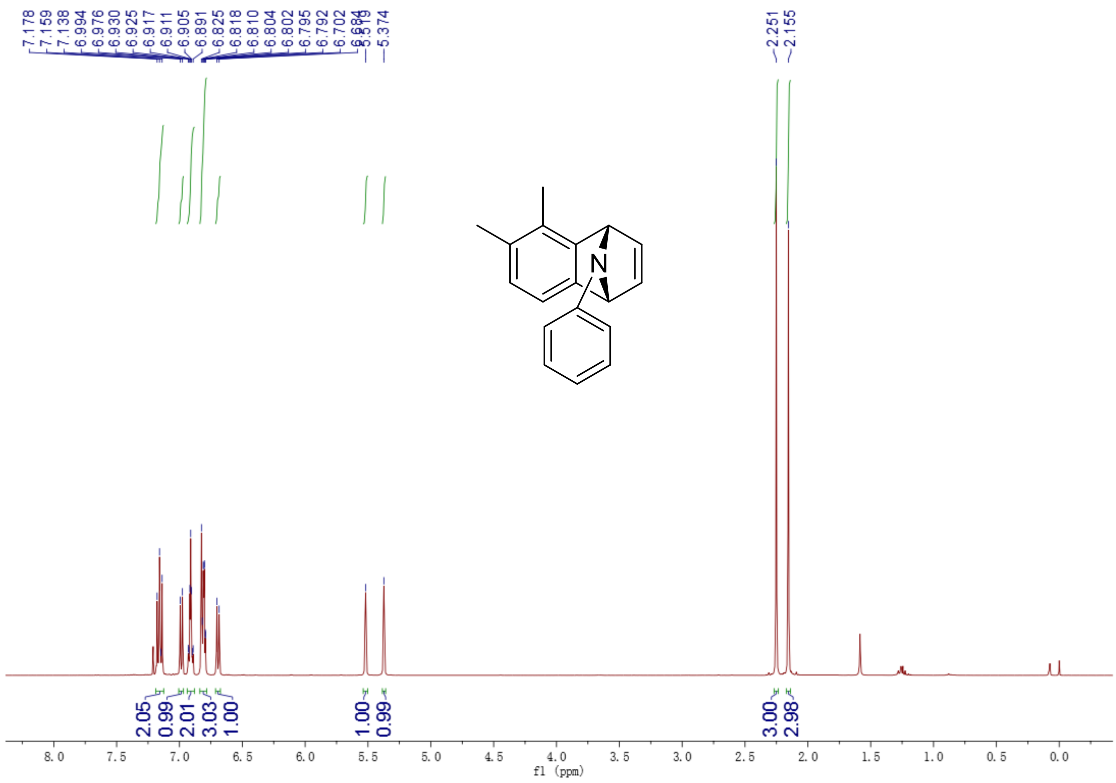

7.178
7.159
7.138
6.994
6.976
6.930
6.925
6.917
6.911
6.905
6.891
6.825
6.818
6.810
6.804
6.802
6.795
6.792
6.702
6.684
5.519
5.374
2.251
2.155
N
2.05
0.99
2.01
3.03
1.00
1.00
0.99
3.00
2.98
f1 (ppm)

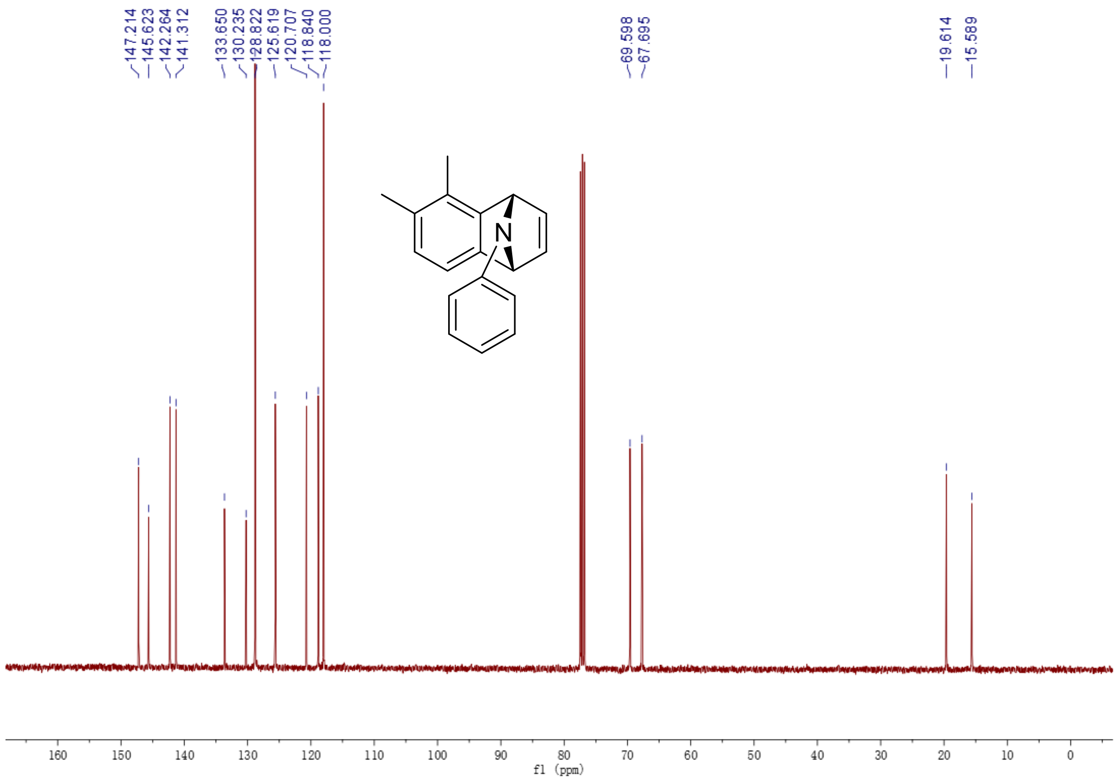

147.214
145.623
142.264
141.312
133.650
130.235
128.822
125.619
120.707
118.840
118.000
69.598
67.695
19.614
15.589
N
f1 (ppm)

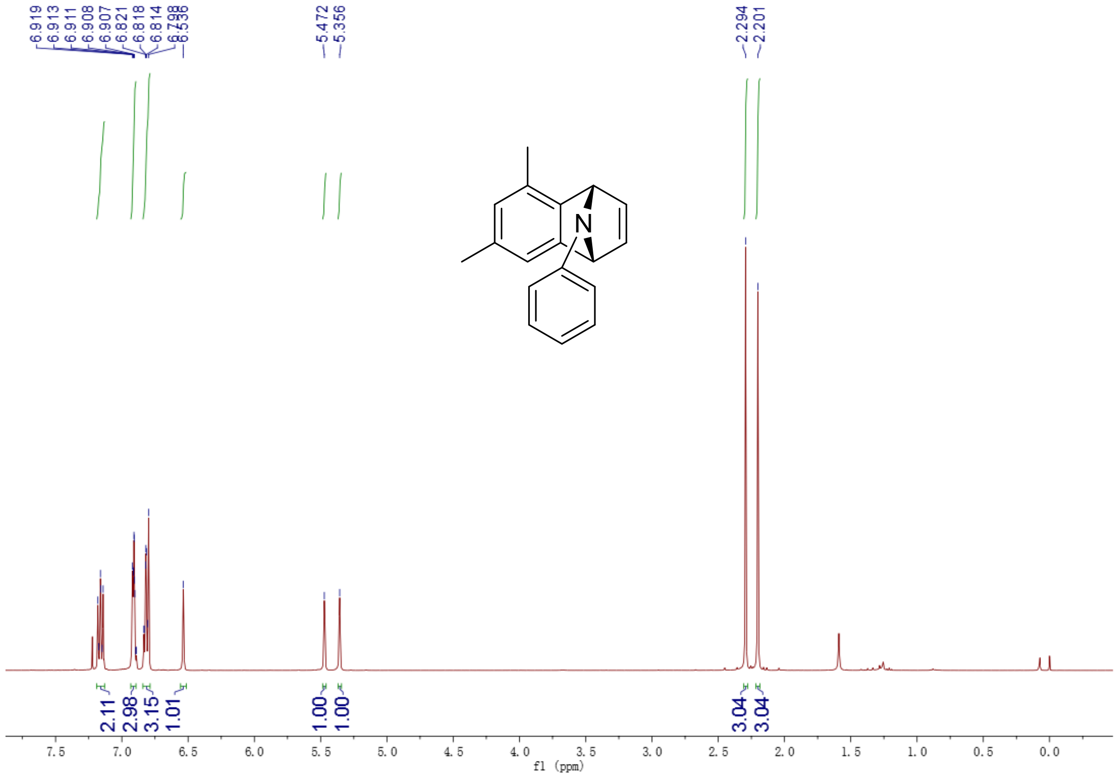
6.919
6.913
6.911
6.908
6.907
6.821
6.818
6.814
6.798
6.536
5.472
5.356
2.294
2.201
N
2.11
2.98
3.15
1.01
1.00
1.00
3.04
3.04
f1 (ppm)

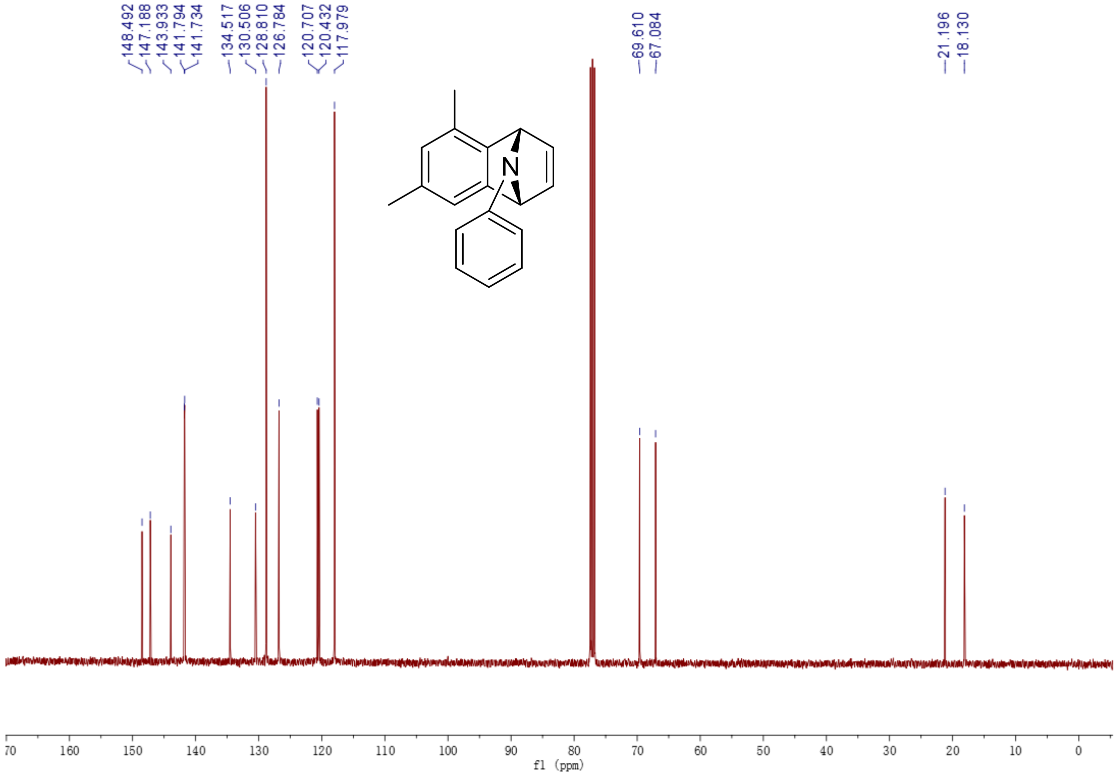
148.492
147.188
143.933
141.794
141.734
134.517
130.506
128.810
126.784
120.707
120.432
117.979
69.610
67.084
21.196
18.130
N
f1 (ppm)

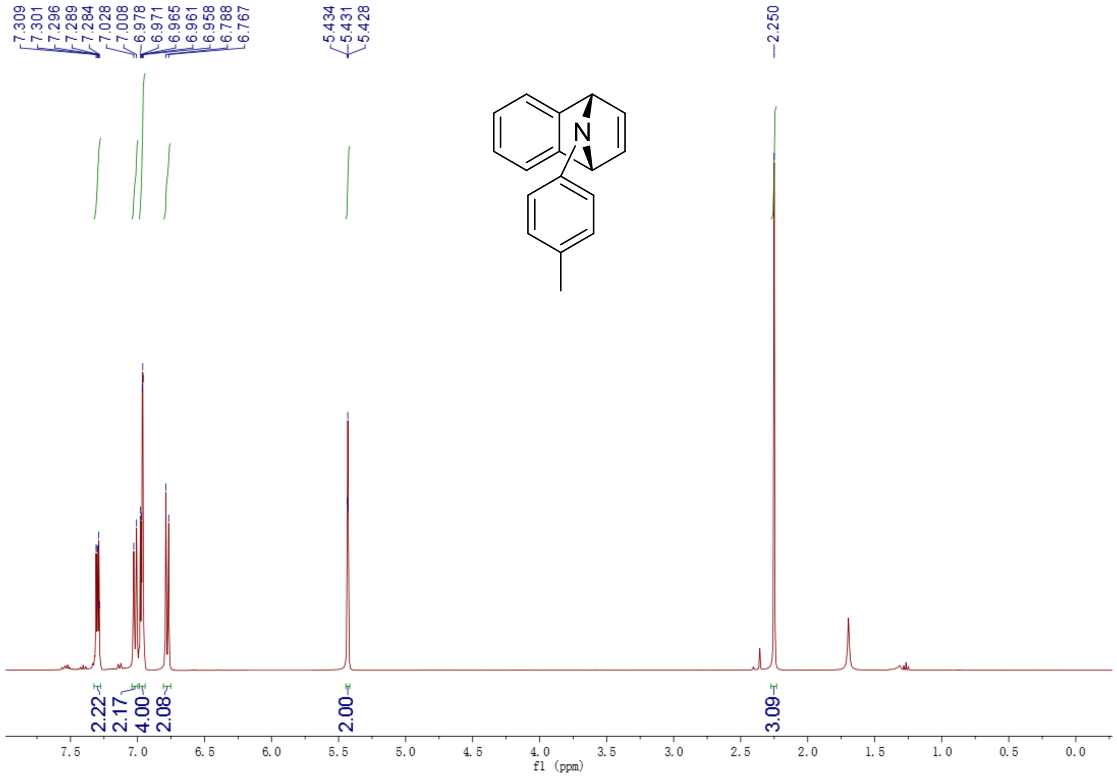
7.309
7.301
7.296
7.289
7.284
7.028
7.008
6.978
6.971
6.965
6.961
6.958
6.788
6.767
5.434
5.431
5.428
2.250
N
2.22
2.17
4.00
2.08
2.00
3.09
f1 (ppm)

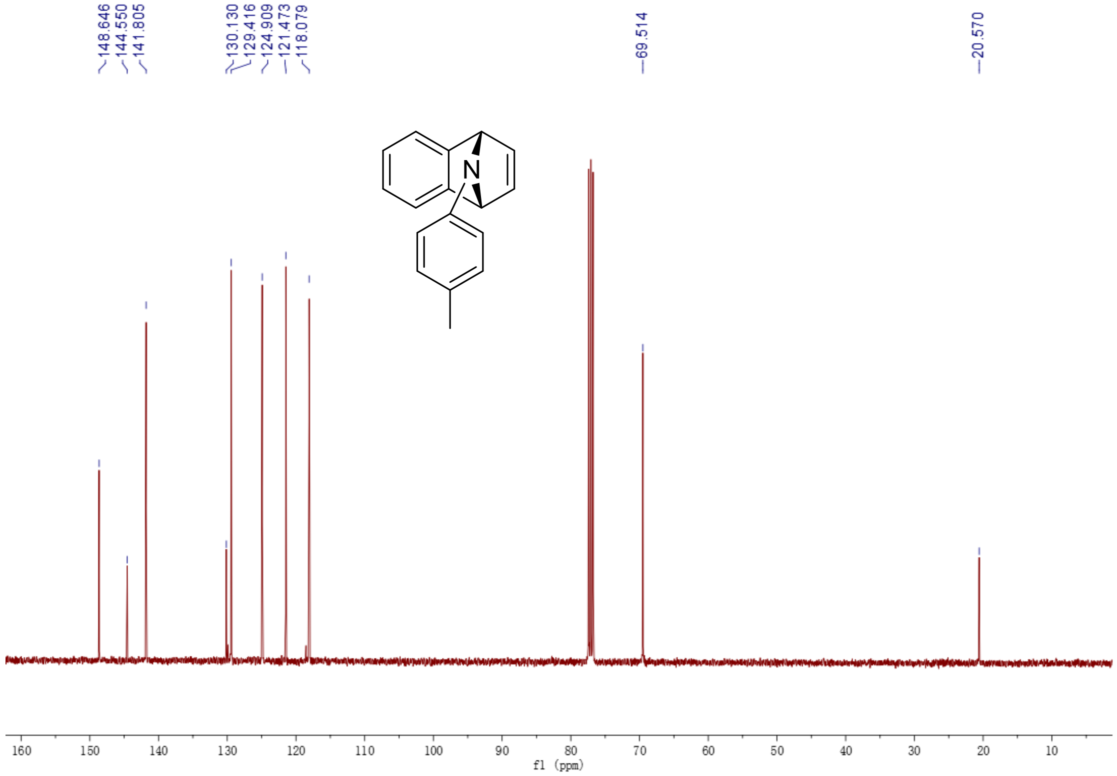
148.646
144.550
141.805
130.130
129.416
124.909
121.473
118.079
69.514
20.570
N
f1 (ppm)

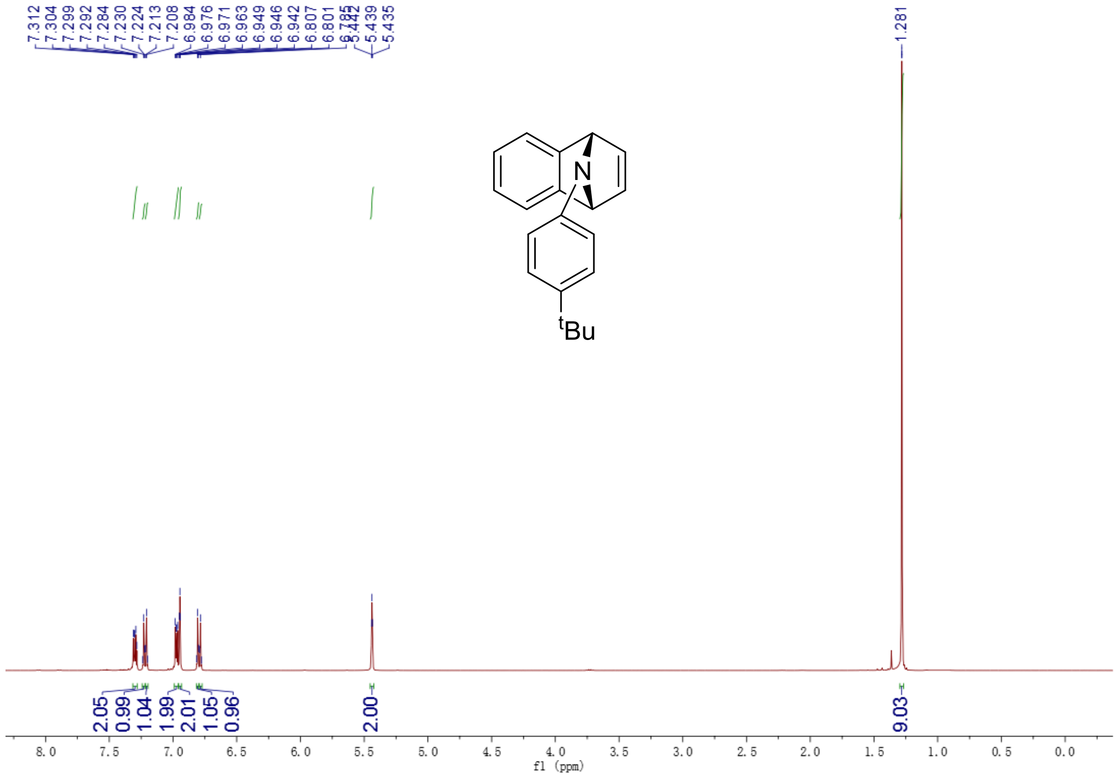
7.312
7.304
7.299
7.292
7.284
7.230
7.224
7.213
7.208
6.984
6.976
6.971
6.963
6.949
6.946
6.942
6.807
6.801
6.785
5.442
5.439
5.435
1.281
tBu
N
2.05
0.99
1.04
1.99
2.01
1.05
0.96
2.00
9.03
8.0
7.5
7.0
6.5
6.0
5.5
5.0
4.5
4.0
3.5
3.0
2.5
2.0
1.5
1.0
0.5
0.0
f1 (ppm)

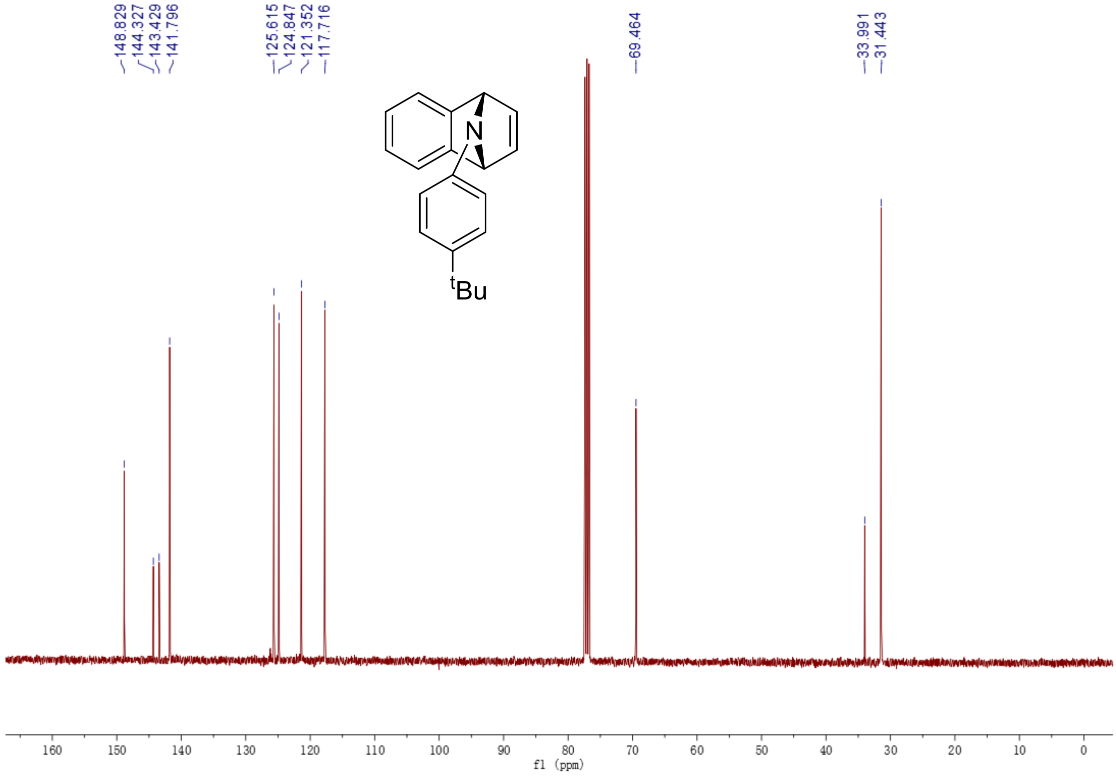
148.829
144.327
143.429
141.796
125.615
124.847
121.352
117.716
69.464
33.991
31.443
tBu
N
160
150
140
130
120
110
100
90
80
70
60
50
40
30
20
10
0
f1 (ppm)

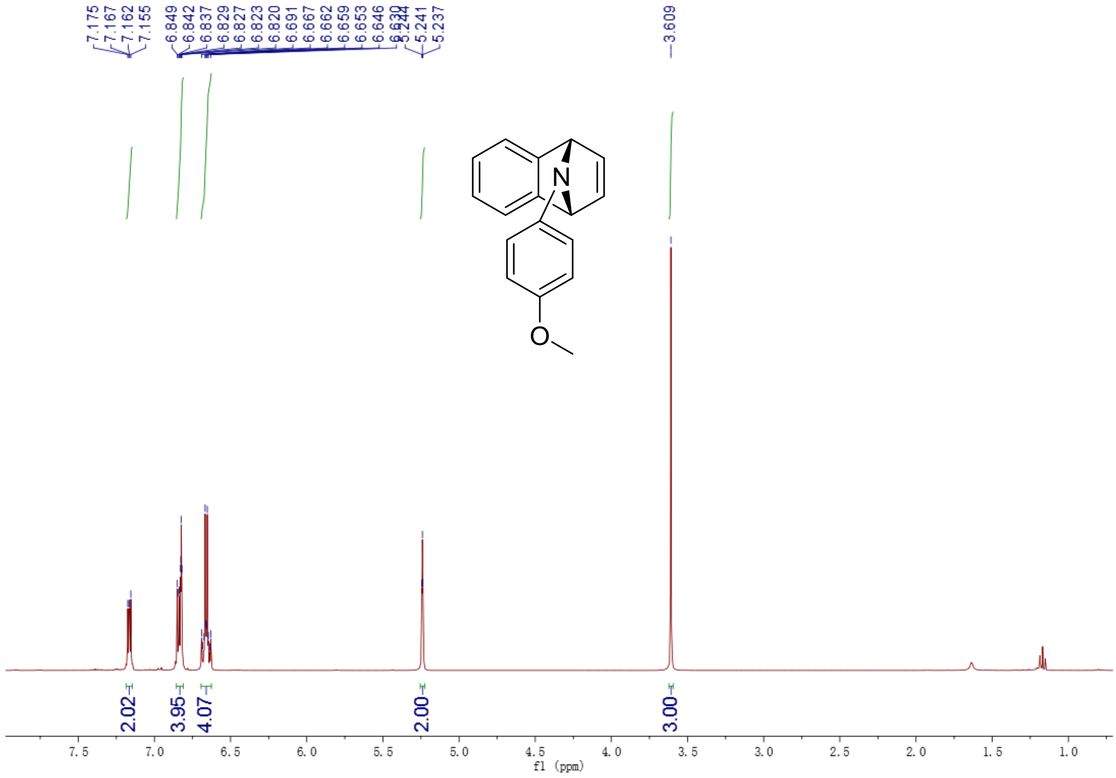

7.175
7.167
7.162
7.155
6.849
6.842
6.837
6.829
6.827
6.823
6.820
6.691
6.667
6.662
6.659
6.653
6.646
6.630
5.244
5.241
5.237
3.609
2.02
3.95
4.07
2.00
3.00
N
O
f1 (ppm)

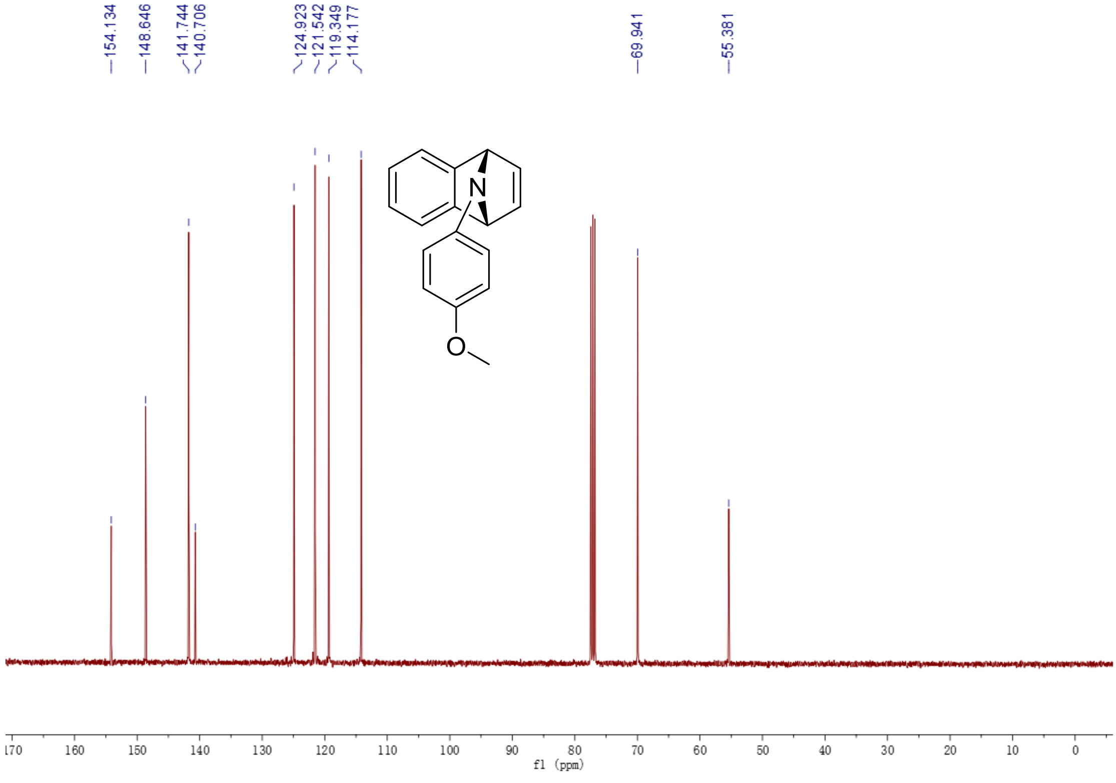

154.134
148.646
141.744
140.706
124.923
121.542
119.349
114.177
69.941
55.381
N
O
f1 (ppm)

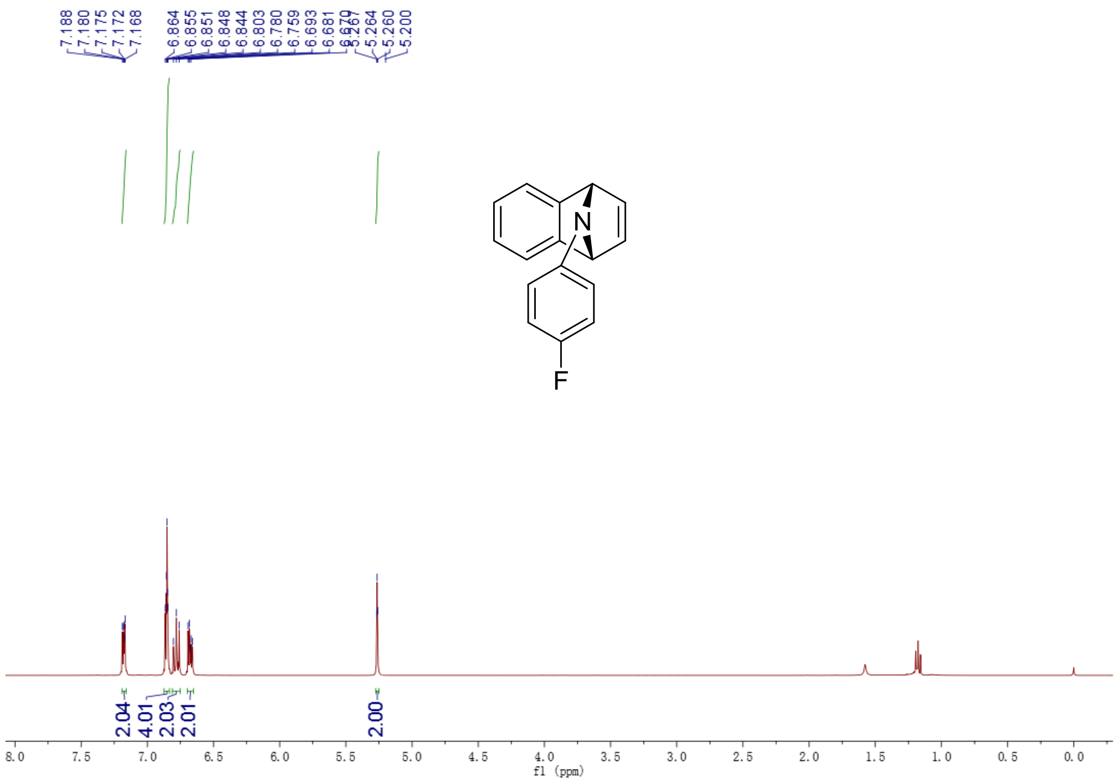
7.188
7.180
7.175
7.172
7.168
6.864
6.855
6.851
6.848
6.844
6.803
6.780
6.759
6.693
6.681
6.679
5.267
5.264
5.260
5.200
N
F
2.04
4.01
2.03
2.01
2.00
8.0
7.5
7.0
6.5
6.0
5.5
5.0
4.5
4.0
3.5
3.0
2.5
2.0
1.5
1.0
0.5
0.0
f1 (ppm)

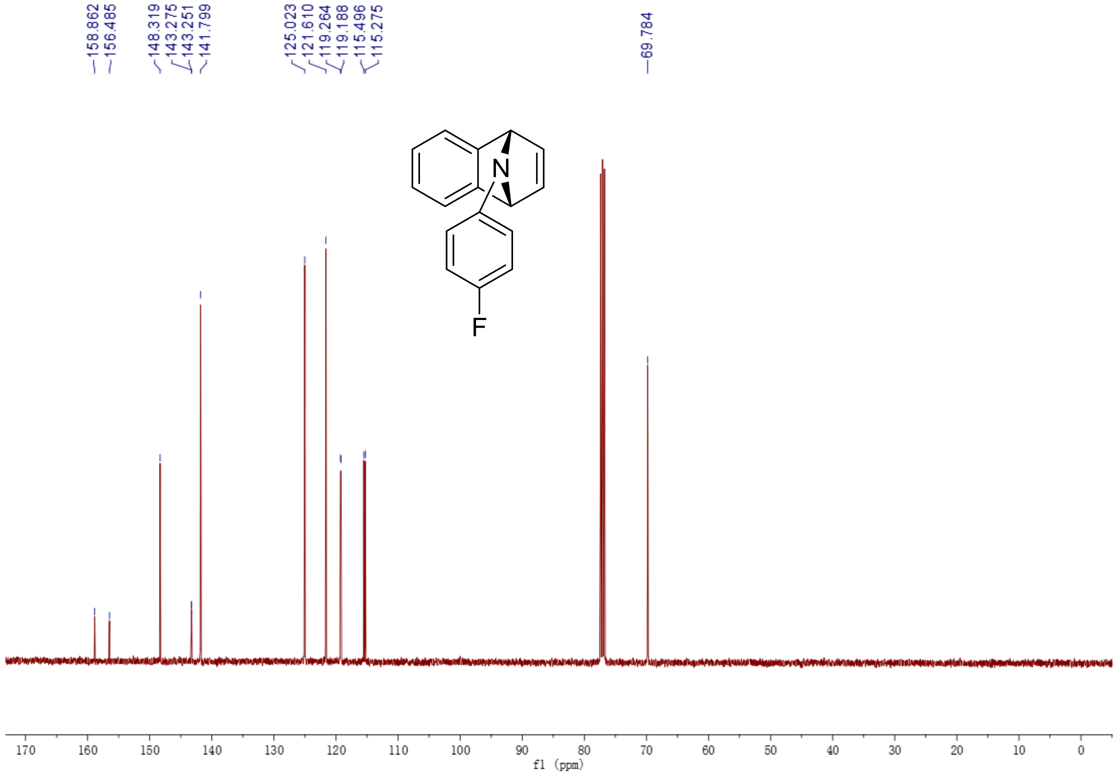
158.862
156.485
148.319
143.275
143.251
141.799
125.023
121.610
119.264
119.188
115.496
115.275
69.784
N
F
170
160
150
140
130
120
110
100
90
80
70
60
50
40
30
20
10
0
f1 (ppm)

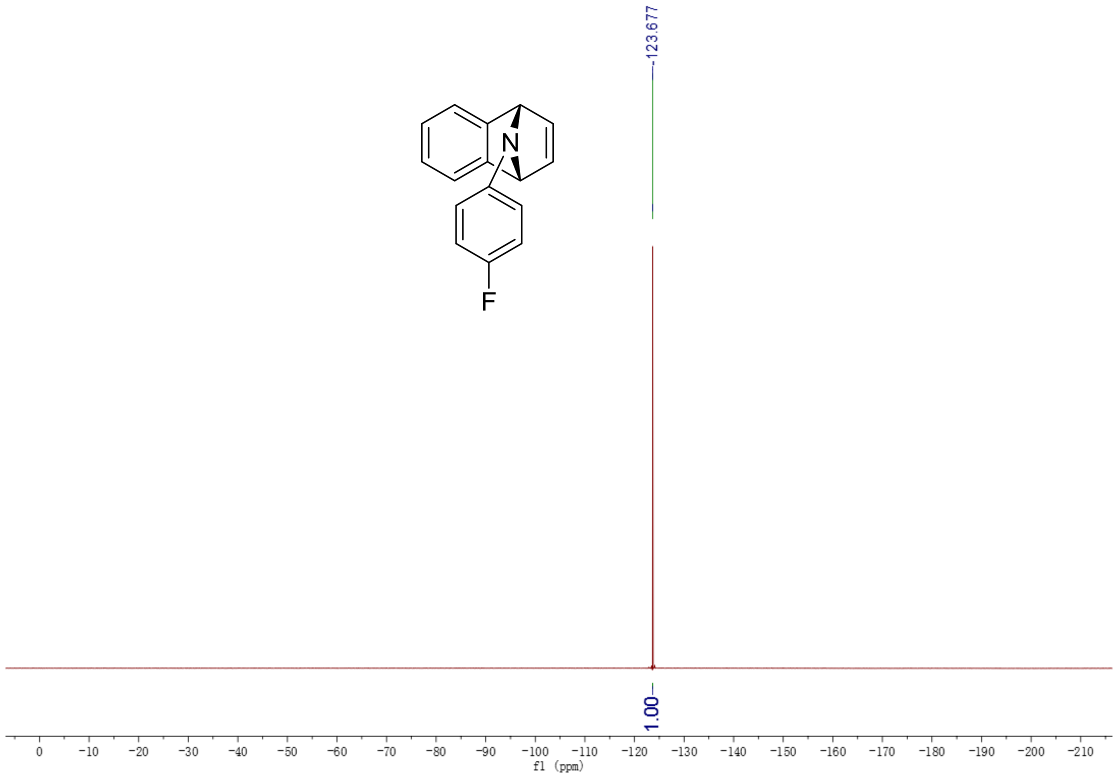

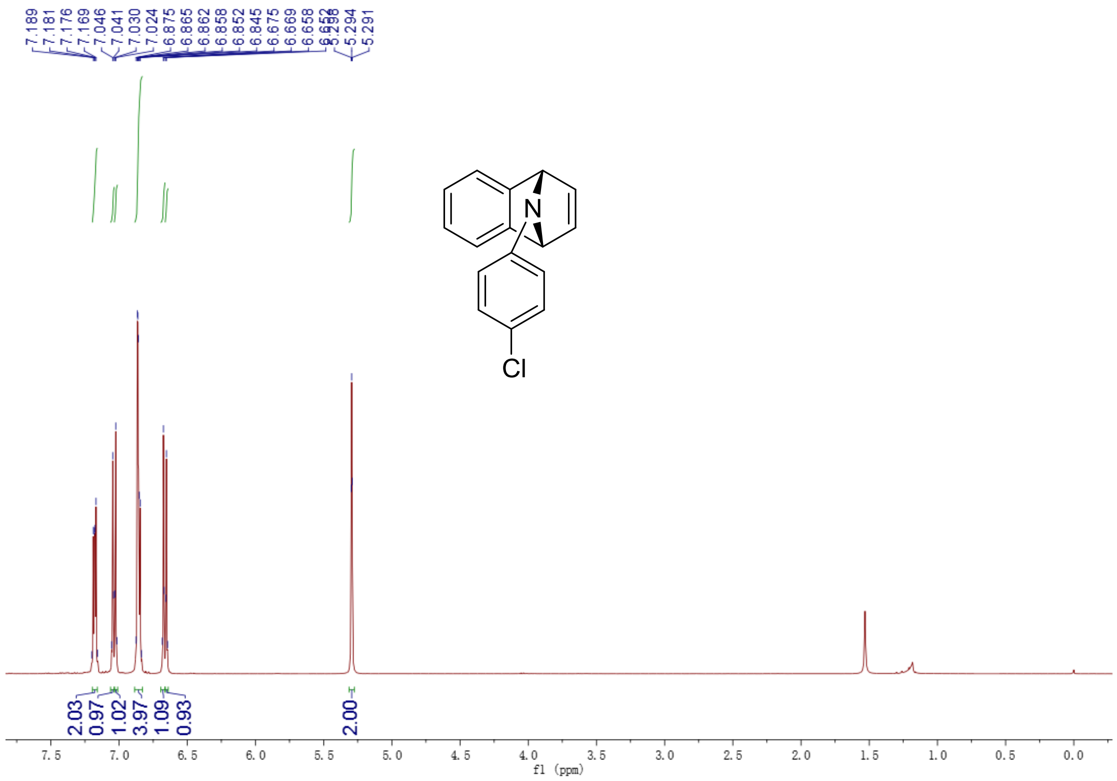

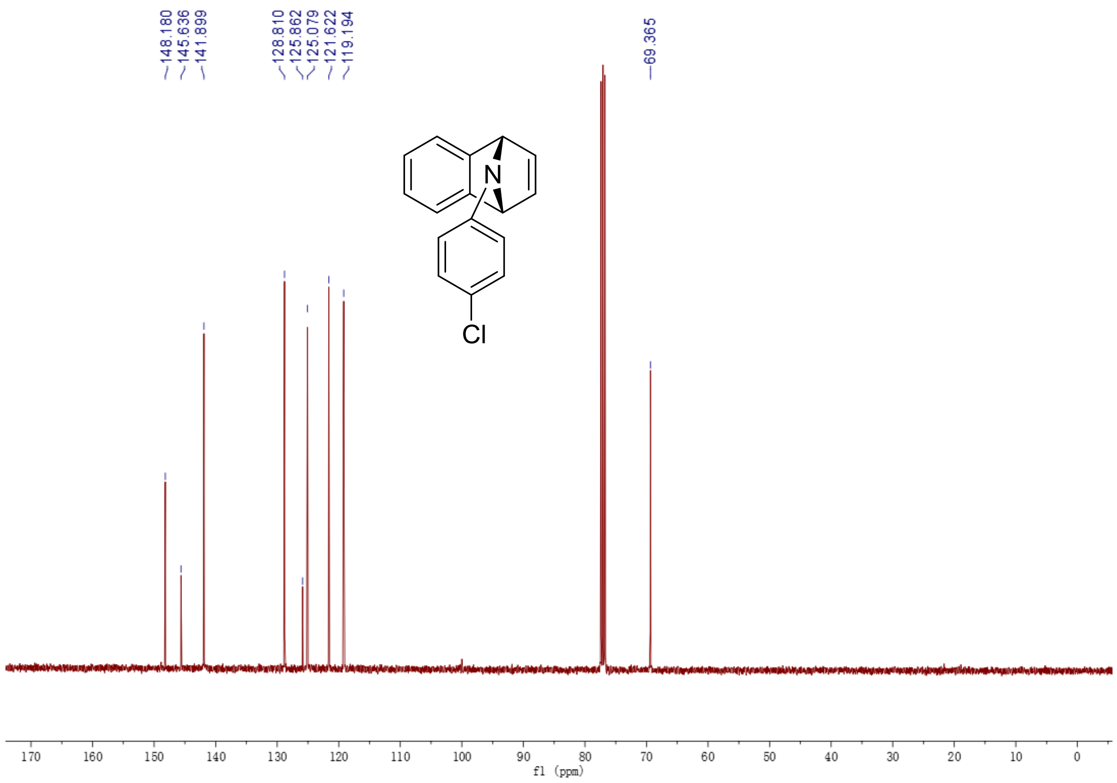
148.180
145.636
141.899
128.810
125.862
125.079
121.622
119.194
69.365
N
Cl
f1 (ppm)

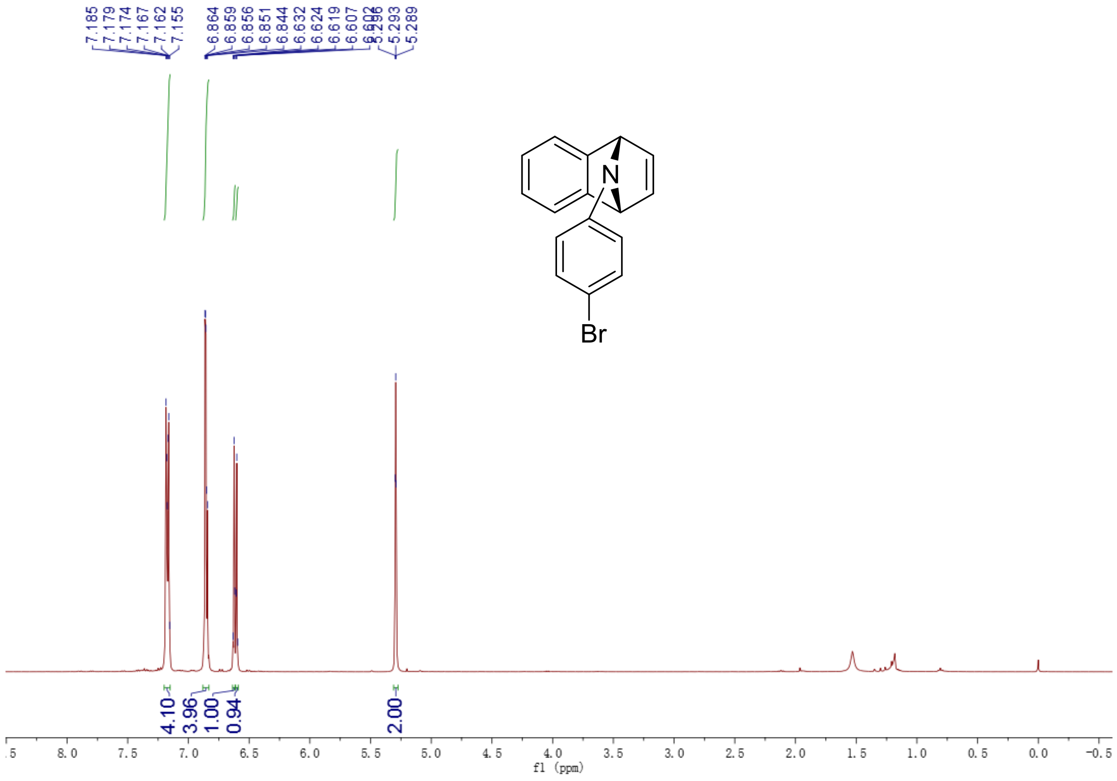
7.185
7.179
7.174
7.167
7.162
7.155
6.864
6.859
6.856
6.851
6.844
6.632
6.624
6.619
6.607
5.293
5.289
N
Br
4.10
3.96
1.00
0.94
2.00
f1 (ppm)

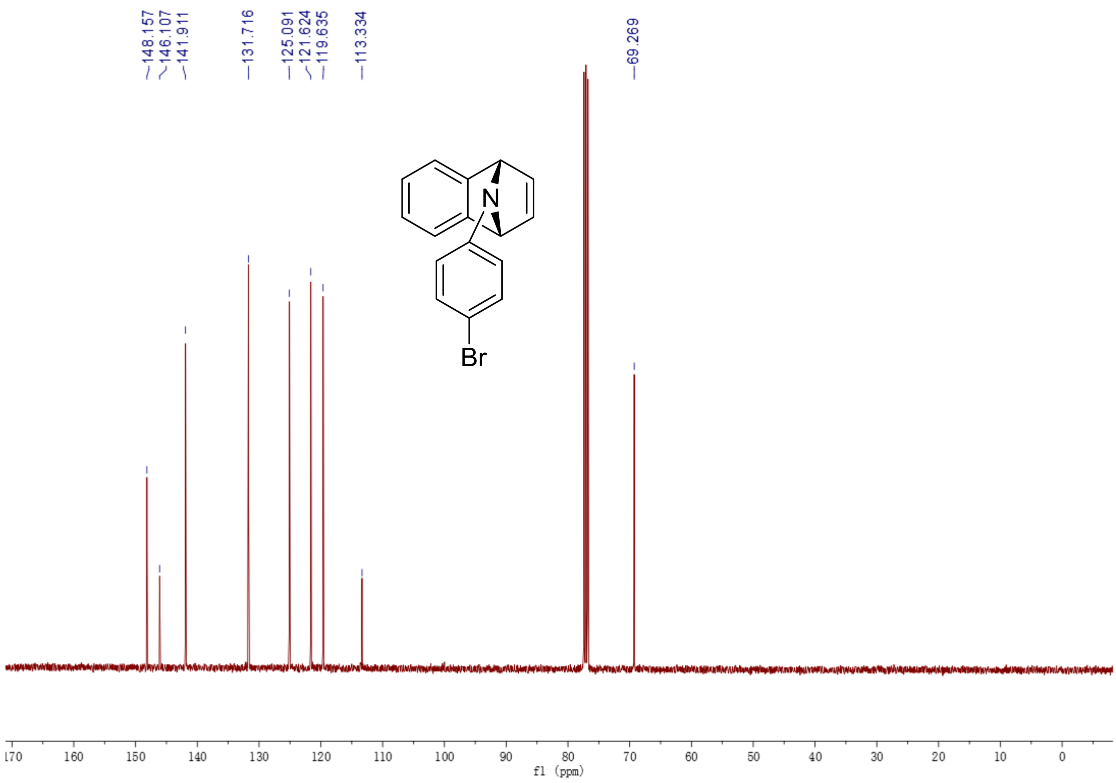

148.157
146.107
141.911
131.716
125.091
121.624
119.635
113.334
69.269
N
Br
170 160 150 140 130 120 110 100 90 80 70 60 50 40 30 20 10 0
f1 (ppm)

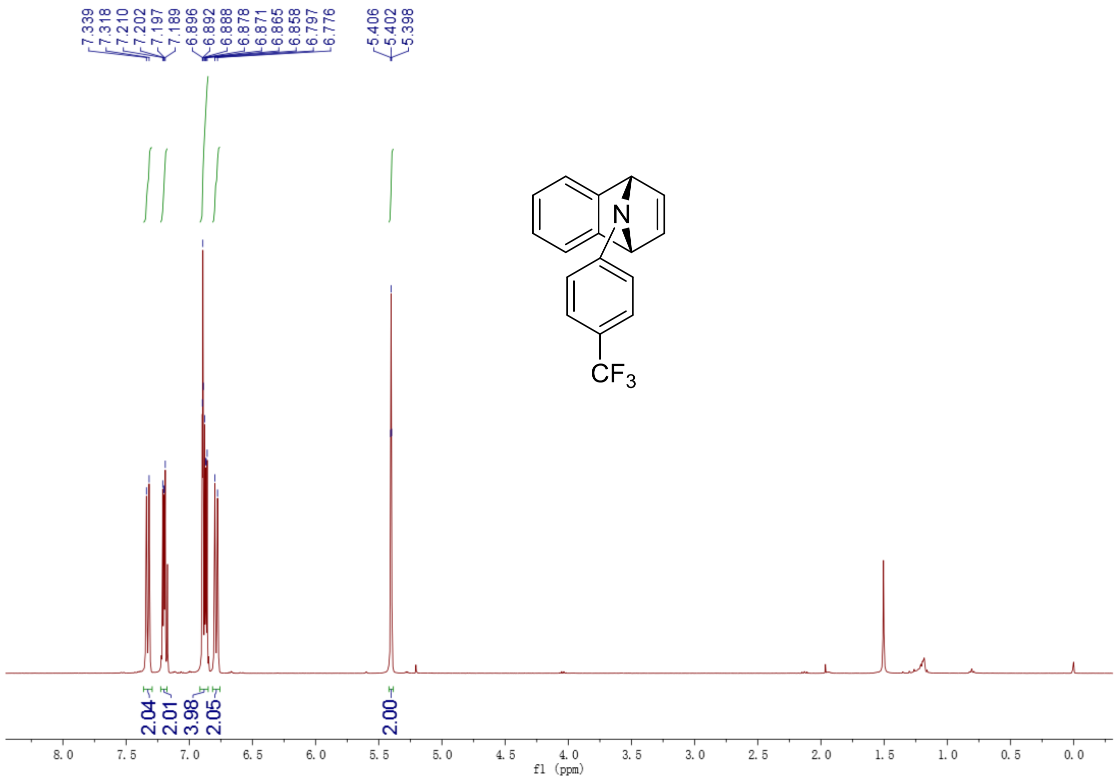

7.339
7.318
7.210
7.202
7.197
7.189
6.896
6.892
6.888
6.878
6.871
6.865
6.858
6.797
6.776
5.406
5.402
5.398
N
CF3
2.04
2.01
3.98
2.05
2.00
8.0 7.5 7.0 6.5 6.0 5.5 5.0 4.5 4.0 3.5 3.0 2.5 2.0 1.5 1.0 0.5 0.0
f1 (ppm)

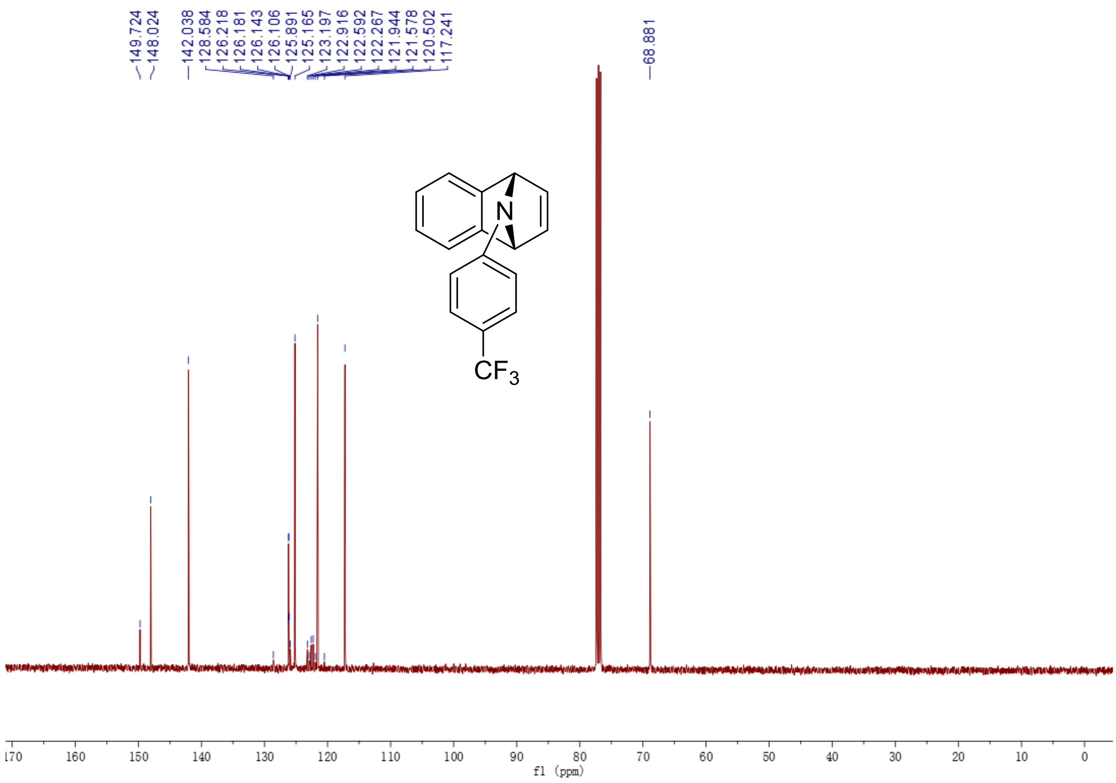
149.724
148.024
142.038
128.584
126.218
126.181
126.143
126.106
125.891
125.165
123.197
122.916
122.592
122.267
121.944
121.578
120.502
117.241
68.881
N
CF3
f1 (ppm)

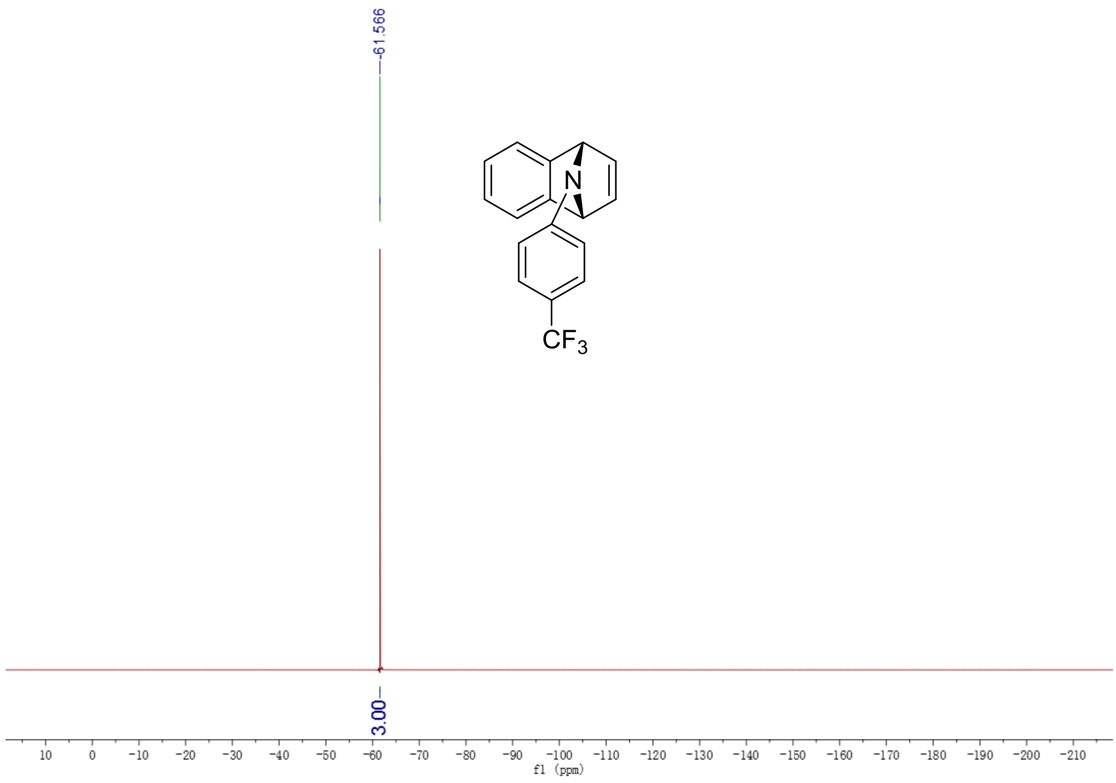
-61.566
N
CF3
3.00
f1 (ppm)

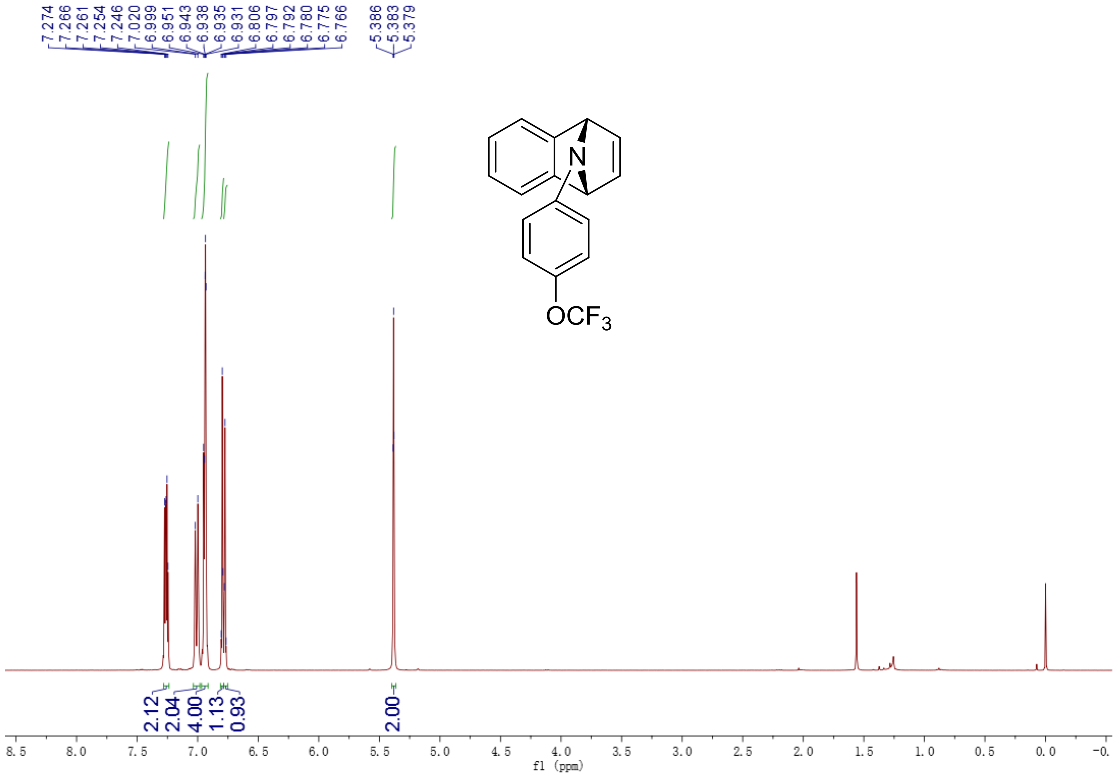
7.274
7.266
7.261
7.254
7.246
7.020
6.999
6.951
6.943
6.938
6.935
6.931
6.806
6.797
6.792
6.780
6.775
6.766
5.386
5.383
5.379
N
OCF3
2.12
2.04
4.00
1.13
0.93
2.00
8.5 8.0 7.5 7.0 6.5 6.0 5.5 5.0 4.5 4.0 3.5 3.0 2.5 2.0 1.5 1.0 0.5 0.0 -0.
f1 (ppm)

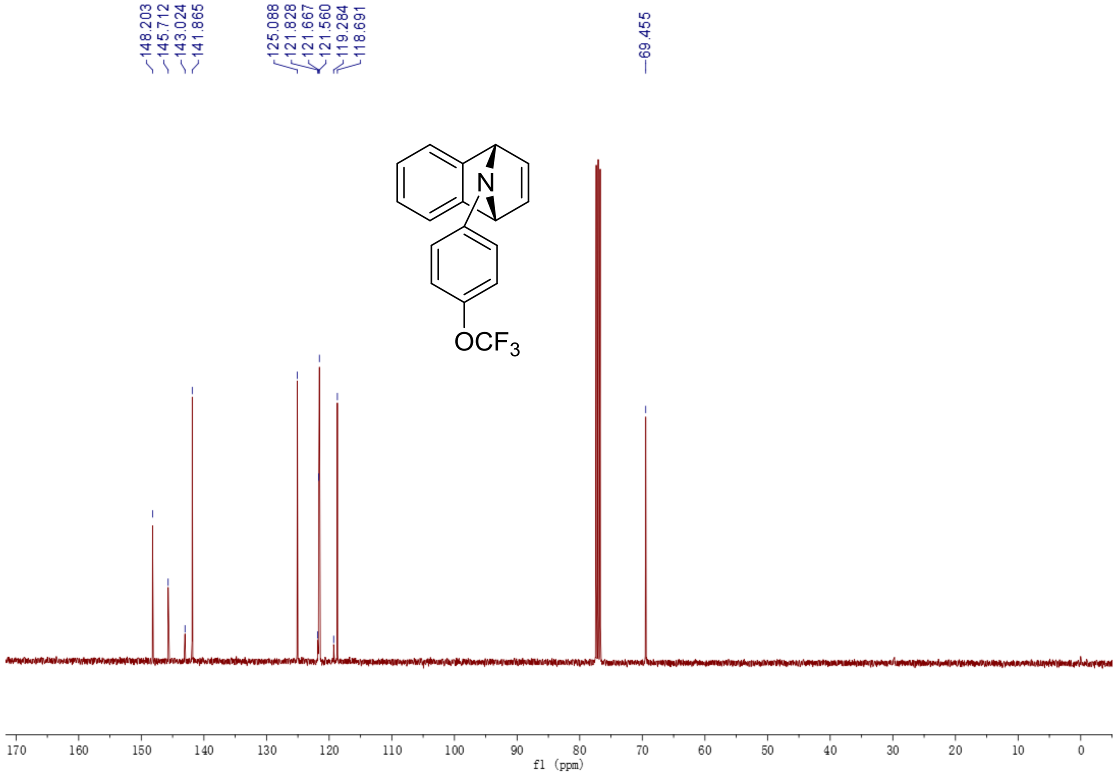
148.203
145.712
143.024
141.865
125.088
121.828
121.667
121.560
119.284
118.691
69.455
N
OCF3
170 160 150 140 130 120 110 100 90 80 70 60 50 40 30 20 10 0
f1 (ppm)

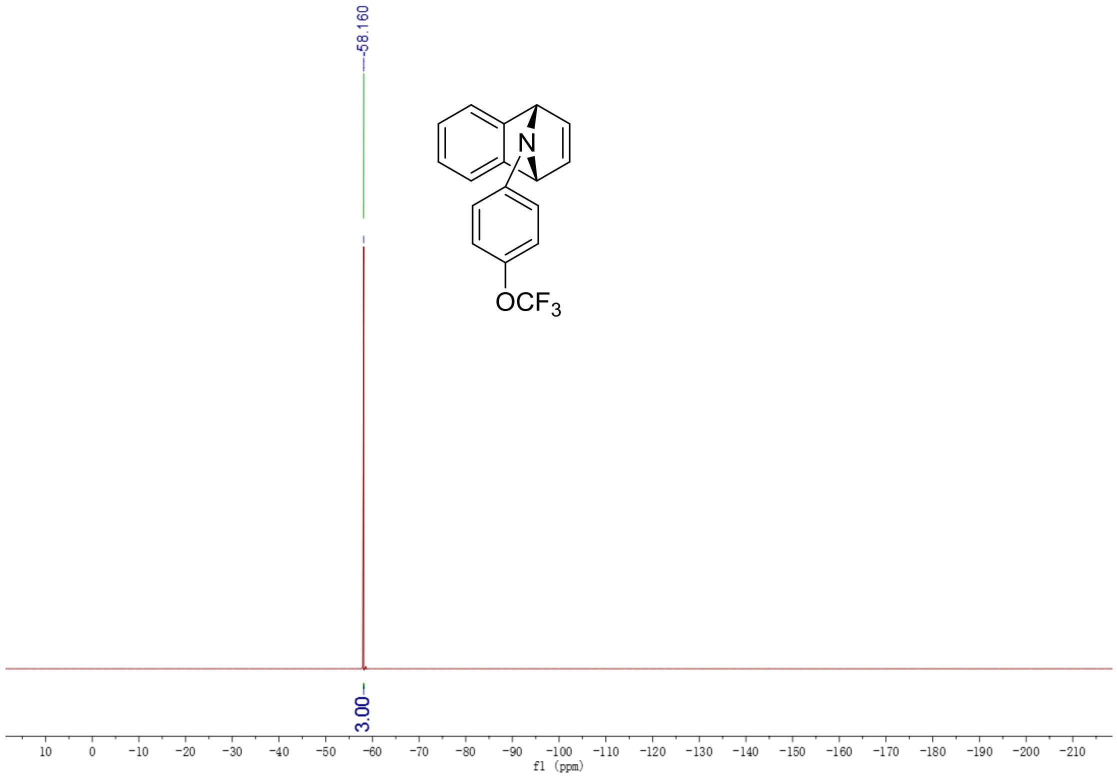

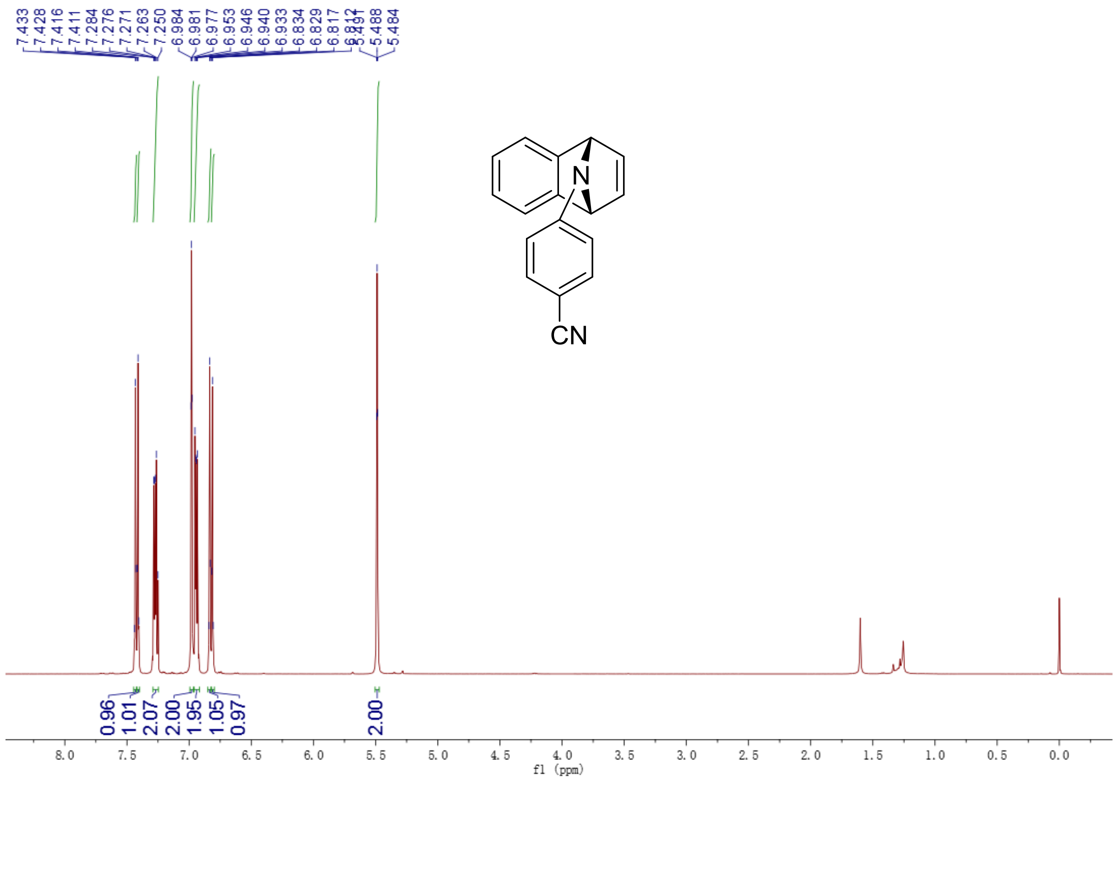

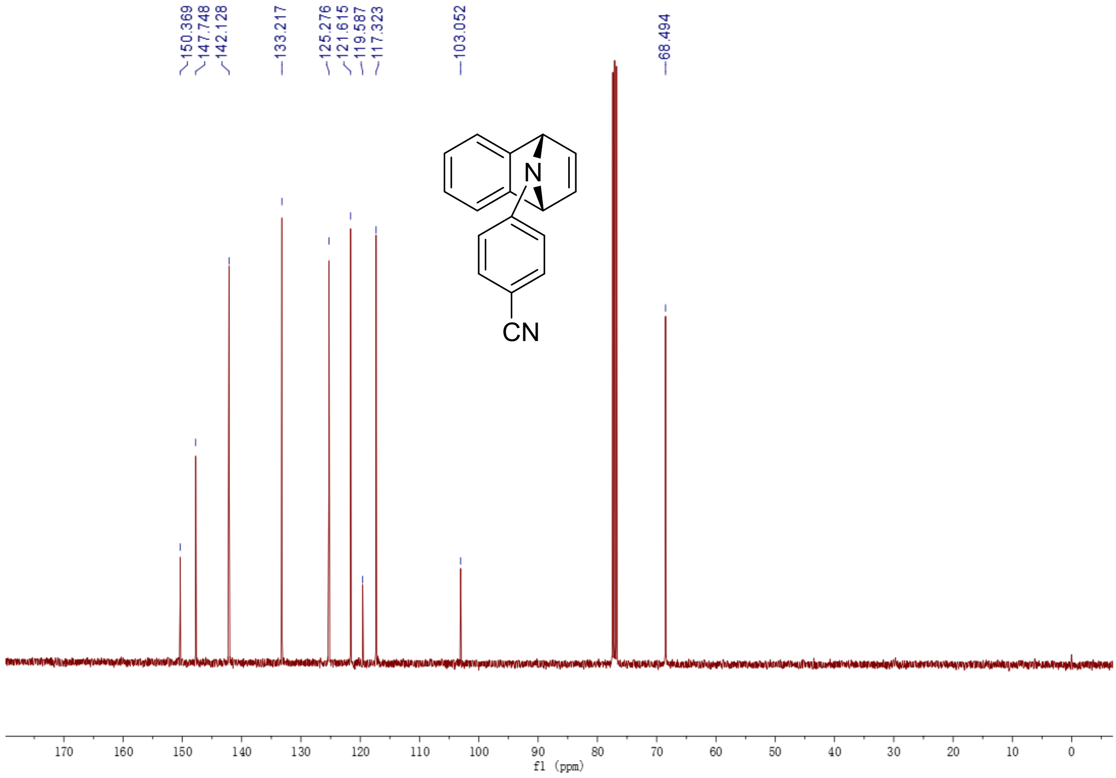

150.369
147.748
142.128
133.217
125.276
121.615
119.587
117.323
103.052
68.494
CN
N
170
160
150
140
130
120
110
100
90
80
70
60
50
40
30
20
10
0
f1 (ppm)

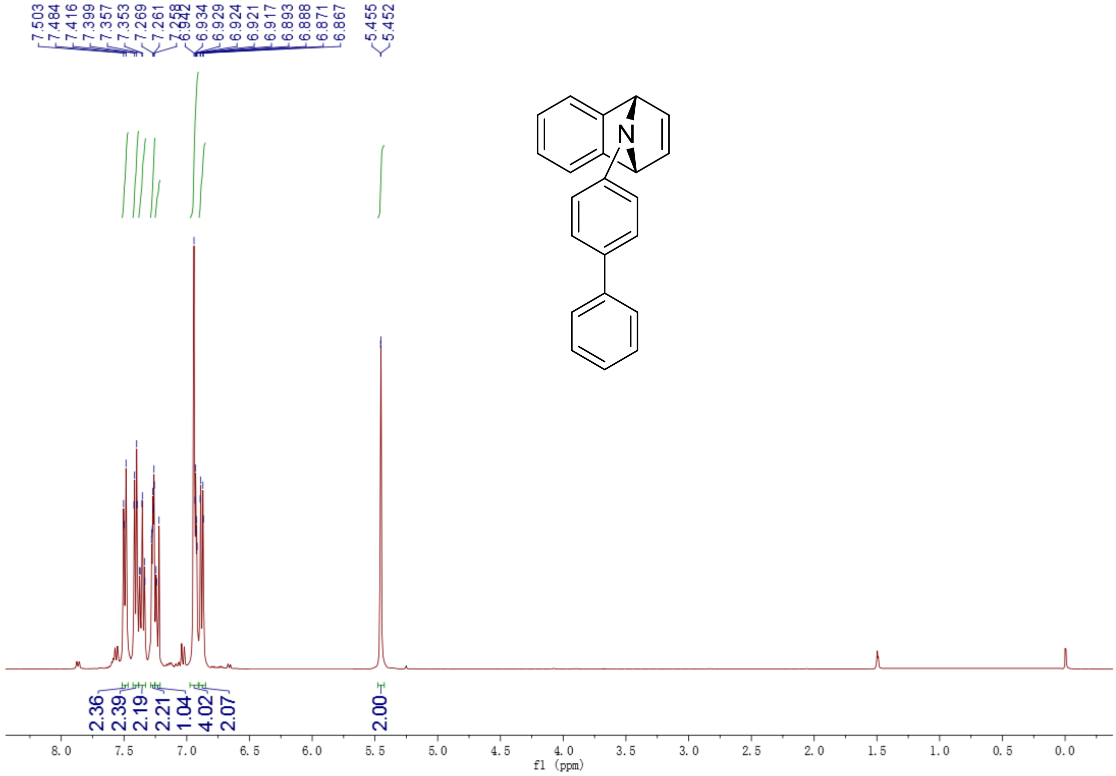

7.503
7.484
7.416
7.399
7.357
7.353
7.269
7.261
5.455
5.452
N
2.36
2.39
2.19
2.21
1.04
4.02
2.07
2.00
8.0
7.5
7.0
6.5
6.0
5.5
5.0
4.5
4.0
3.5
3.0
2.5
2.0
1.5
1.0
0.5
0.0
f1 (ppm)

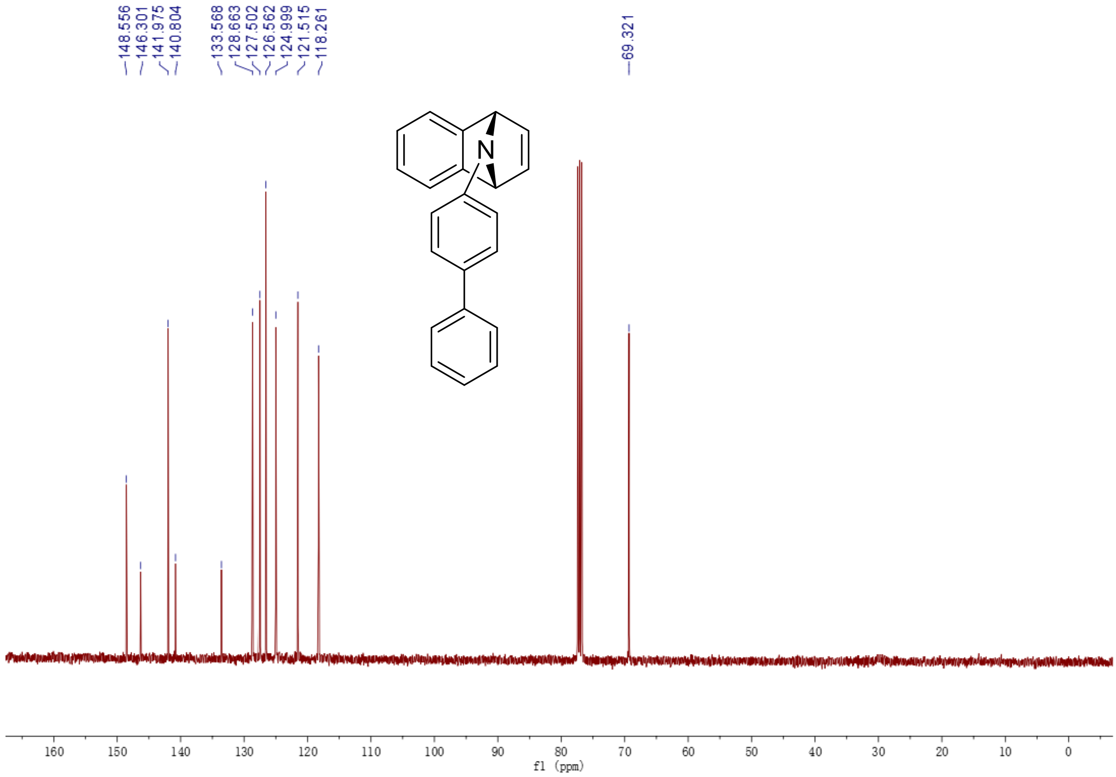

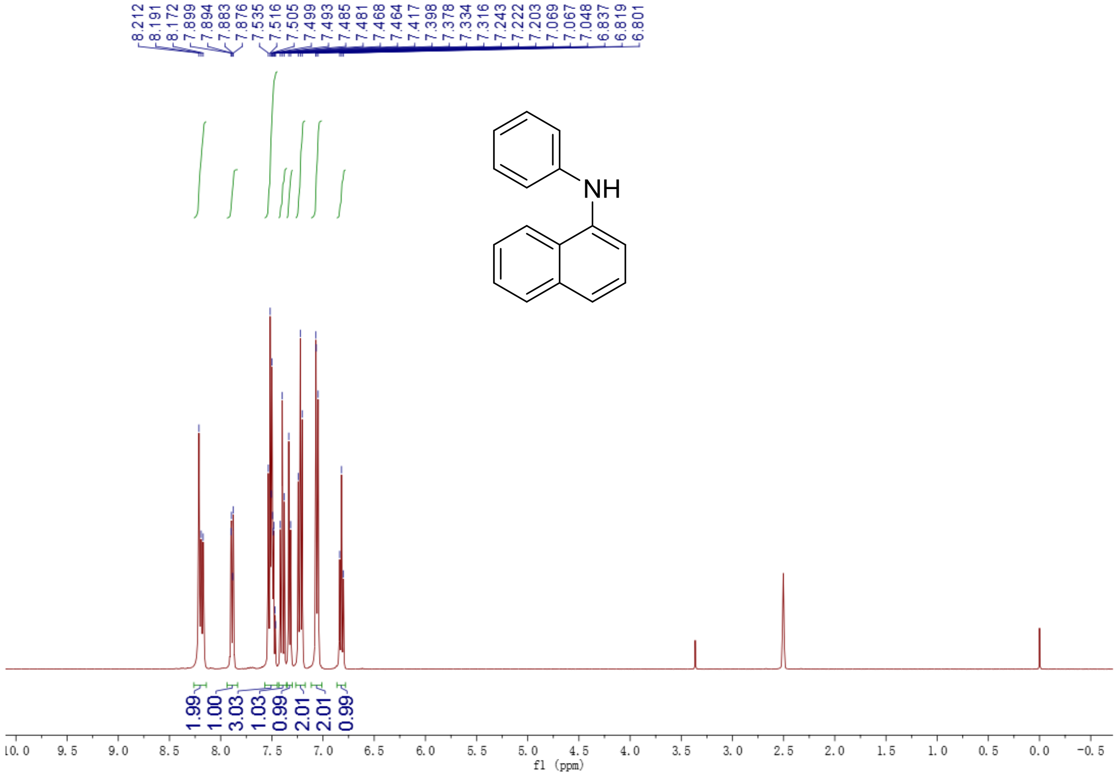

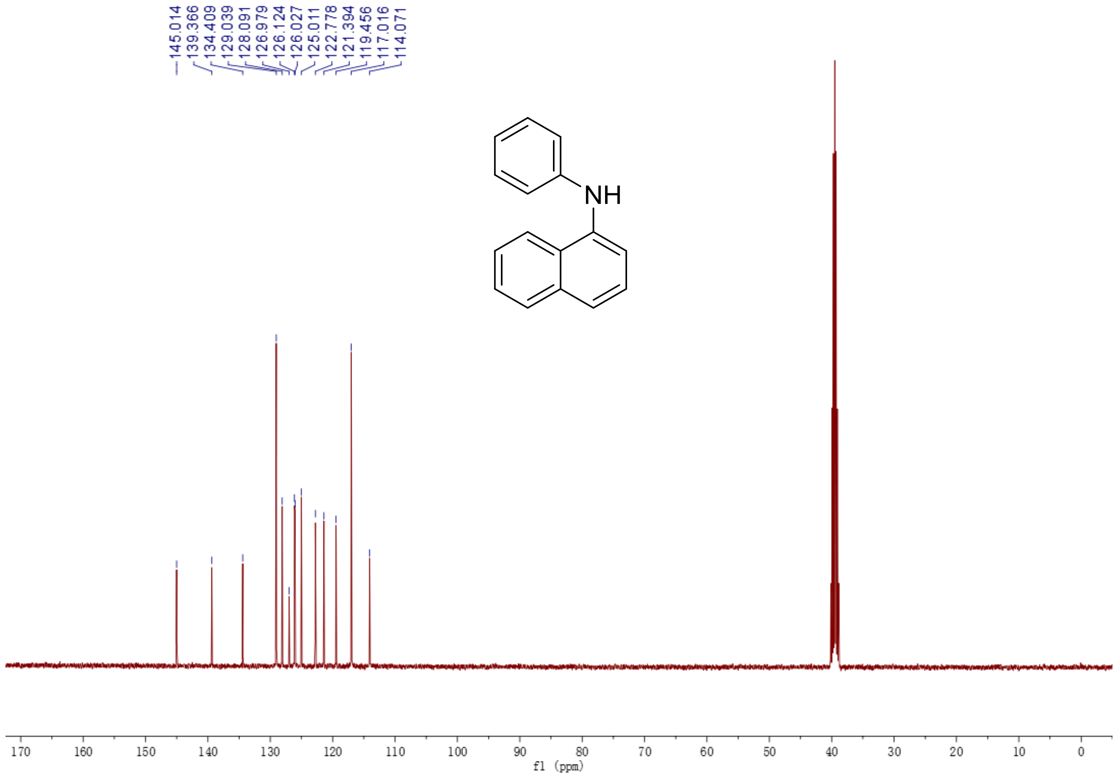
145.014
139.366
134.409
129.039
128.091
126.979
126.124
126.027
125.011
122.778
121.394
119.456
117.016
114.071
NH
170 160 150 140 130 120 110 100 90 80 70 60 50 40 30 20 10 0
f1 (ppm)

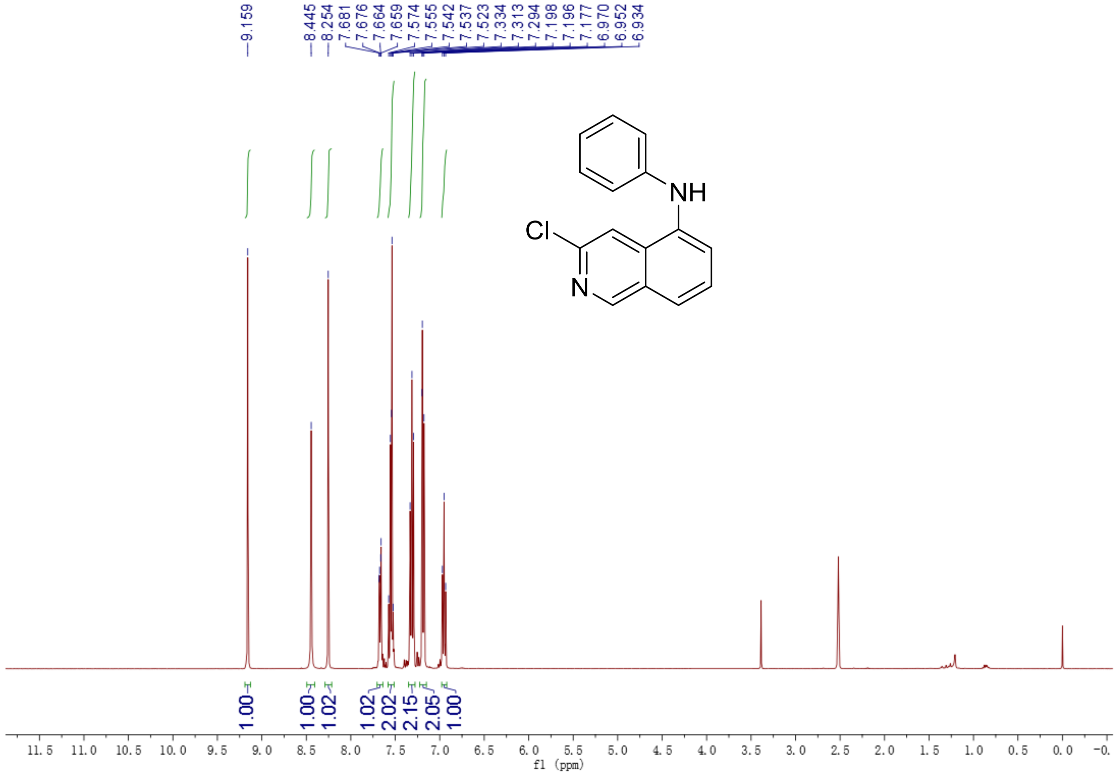
9.159
8.445
8.254
7.681
7.676
7.664
7.659
7.574
7.555
7.542
7.537
7.523
7.334
7.313
7.294
7.198
7.196
7.177
6.970
6.952
6.934
NH
Cl
N
1.00
1.00
1.02
1.02
2.02
2.15
2.05
1.00
11.5 11.0 10.5 10.0 9.5 9.0 8.5 8.0 7.5 7.0 6.5 6.0 5.5 5.0 4.5 4.0 3.5 3.0 2.5 2.0 1.5 1.0 0.5 0.0 -0.
f1 (ppm)

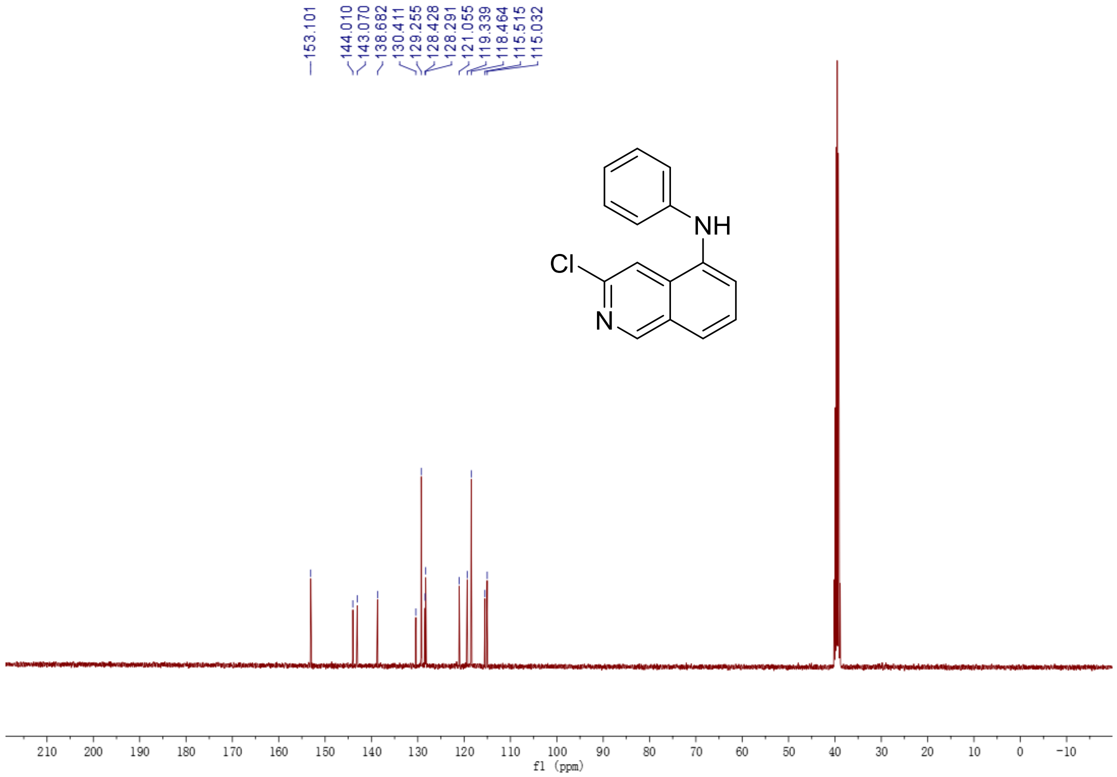

A: ^{1}H NMR spectrum of the product recorded at 400 MHz in DMSO-d_6 at 298K; B: ^{1}H NMR spectrum of the product recorded at 400 MHz in DMSO-d_6 (major) / D_2O (minor) at 298 K.

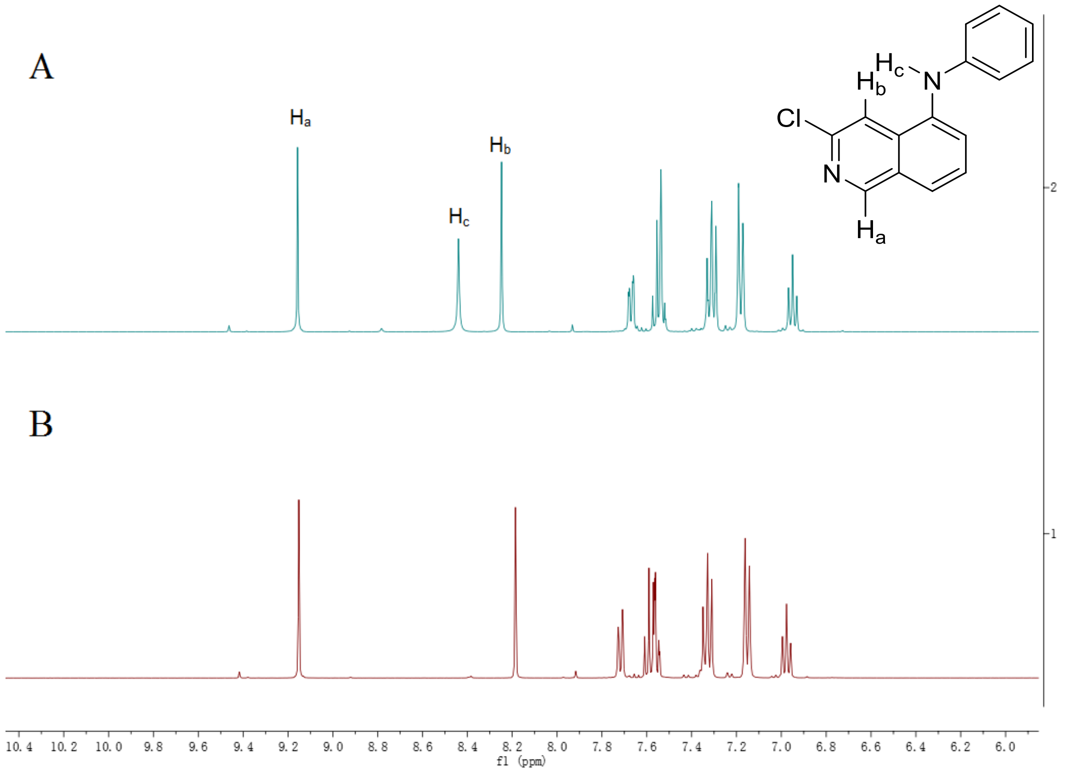

^{1}H-^{1}H NOSY spectrum of the product recorded at 400 MHz in DMSO-d_6 at 298 K

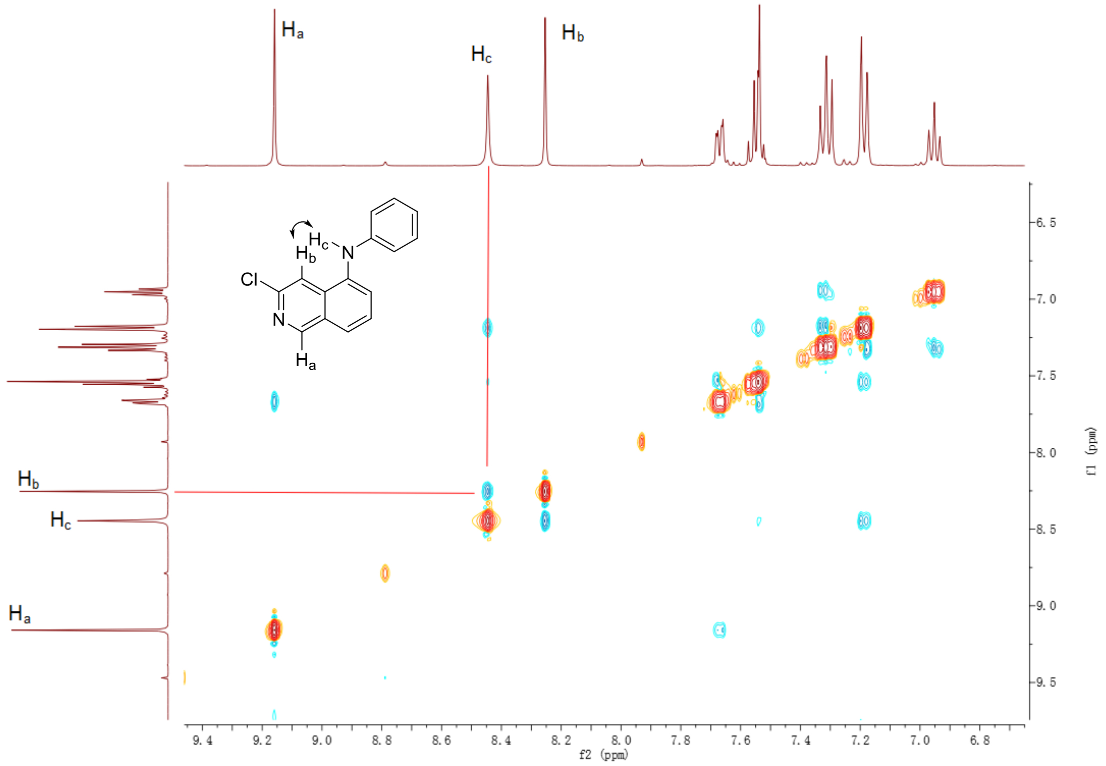

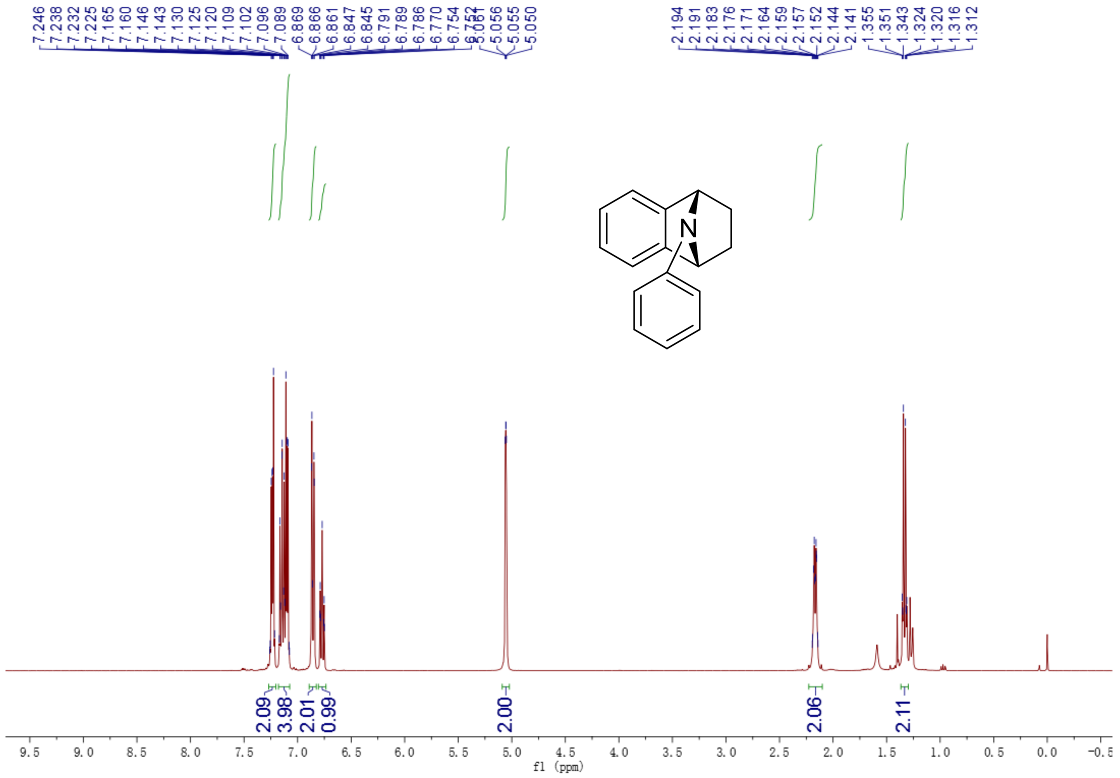

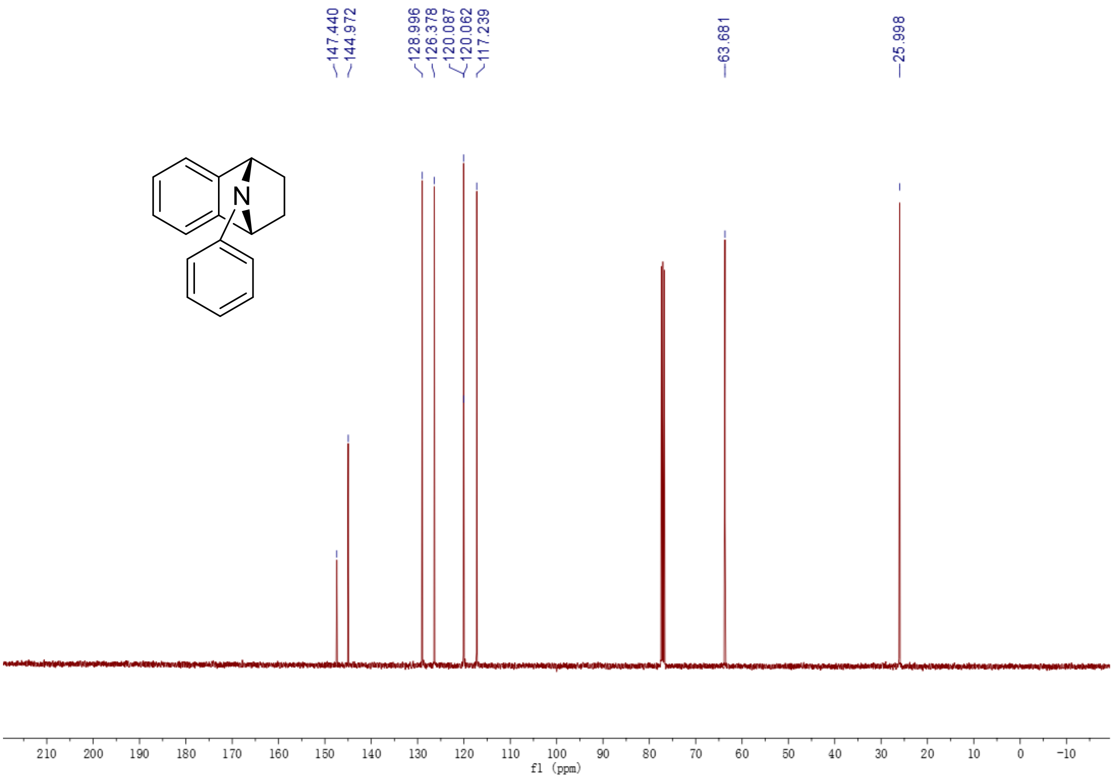

Elemental Composition Report

Page 1

Single Mass Analysis

Tolerance = 50.0 PPM / DBE: min = -1.5, max = 100.0
Element prediction: Off
Number of isotope peaks used for i-FIT = 3

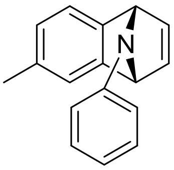

Monoisotopic Mass, Even Electron Ions
16 formula(e) evaluated with 1 results within limits (up to 1 best isotopic matches for each mass)
Elements Used:
C: 0-44 H: 0-57 N: 0-3

LM-WANG ECUST institute of Fine Chem 23-Jun-2017 20:56:22

WL-CHG-110 36 (1.183) Cm (36) 1: TOF MS ES+ 1.74e+003

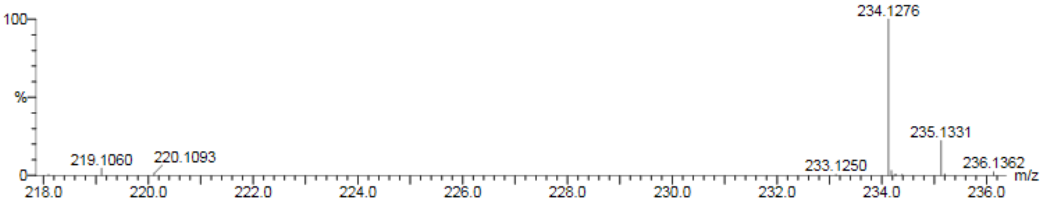

Minimum:				-1.5			
Maximum:		30.0	50.0	100.0			
Mass	Calc. Mass	mDa	PPM	DBE	i-FIT	i-FIT (Norm)	Formula
234.1276	234.1283	-0.7	-3.0	10.5	35.5	0.0	C17 H16 N

Elemental Composition Report

Single Mass Analysis
Tolerance = 50.0 PPM / DBE: min = -1.5, max = 100.0
Element prediction: Off
Number of isotope peaks used for i-FIT = 3

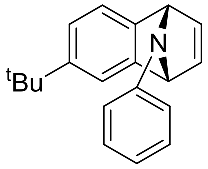

Monoisotopic Mass, Even Electron Ions
1 formula(e) evaluated with 1 results within limits (up to 1 best isotopic matches for each mass)
Elements Used:
C: 0-20 H: 0-22 N: 0-1

LM-WANG ECUST institute of Fine Chem 28-Jun-2017
22:04:29
WL-CHG-118 66 (0.905) Cm (65:67) 1: TOF MS ES+
7.26e+003

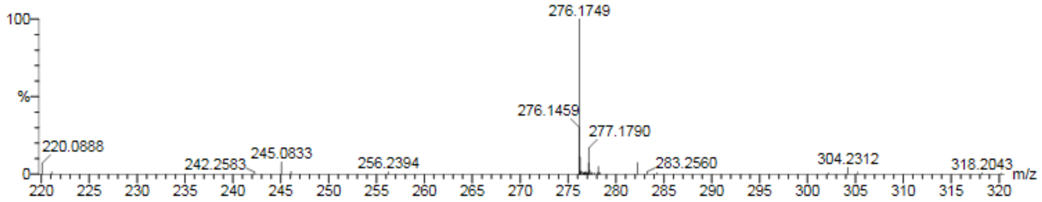

Minimum:				-1.5			
Maximum:		30.0	50.0	100.0			
Mass	Calc. Mass	mDa	PPM	DBE	i-FIT	i-FIT (Norm)	Formula
276.1749	276.1752	-0.3	-1.1	10.5	241.6	0.0	C20 H22 N

Elemental Composition Report

Single Mass Analysis
Tolerance = 50.0 PPM / DBE: min = -1.5, max = 100.0
Element prediction: Off
Number of isotope peaks used for i-FIT = 3

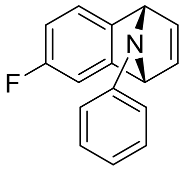

Monoisotopic Mass, Even Electron Ions
2 formula(e) evaluated with 1 results within limits (up to 1 best isotopic matches for each mass)
Elements Used:
C: 0-16 H: 0-13 N: 0-1 F: 0-1

LM-WANG ECUST institute of Fine Chem 30-Jun-2017
12:21:47
WL-CHG-113 12 (0.248) Cm (10:12) 1: TOF MS ES+
6.83e+003

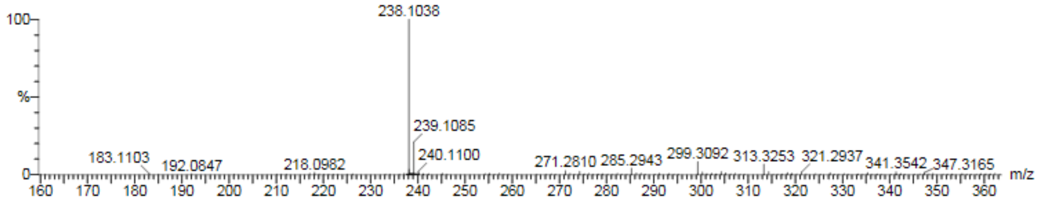

Minimum:				-1.5			
Maximum:		30.0	50.0	100.0			
Mass	Calc. Mass	mDa	PPM	DBE	i-FIT	i-FIT (Norm)	Formula
238.1038	238.1032	0.6	2.5	10.5	213.1	0.0	C16 H13 N F

Elemental Composition Report

Page 1

Single Mass Analysis

Tolerance = 50.0 PPM / DBE: min = -1.5, max = 100.0
Element prediction: Off
Number of isotope peaks used for i-FIT = 3

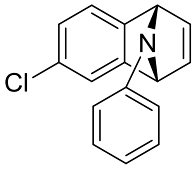

Monoisotopic Mass, Even Electron Ions
7 formula(e) evaluated with 1 results within limits (up to 1 best isotopic matches for each mass)
Elements Used:
C: 0-16 H: 0-100 N: 0-1 Cl: 0-1

LM-WANG ECUST institute of Fine Chem 02-Jul-2017 13:51:29

WL-CHG-112 44 (0.629) Cm (43:45) 1: TOF MS ES+ 3.55e+003

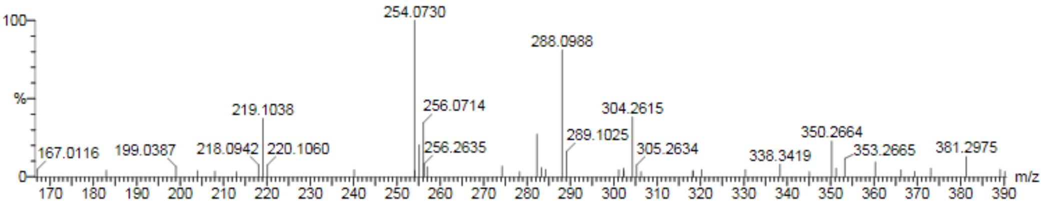

Minimum:				-1.5			
Maximum:		30.0	50.0	100.0			
Mass	Calc. Mass	mDa	PPM	DBE	i-FIT	i-FIT (Norm)	Formula
254.0730	254.0737	-0.7	-2.8	10.5	28.3	0.0	C16 H13 N Cl

Elemental Composition Report

Page 1

Single Mass Analysis

Tolerance = 50.0 PPM / DBE: min = -1.5, max = 100.0
Element prediction: Off
Number of isotope peaks used for i-FIT = 3

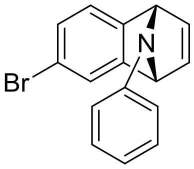

Monoisotopic Mass, Even Electron Ions
7 formula(e) evaluated with 1 results within limits (up to 1 best isotopic matches for each mass)
Elements Used:
C: 0-16 H: 0-57 N: 0-1 Br: 0-1

LM-WANG ECUST institute of Fine Chem 23-Jun-2017 20:59:51

WL-CHG-111 60 (1.920) Cm (59:60) 1: TOF MS ES+ 2.09e+003

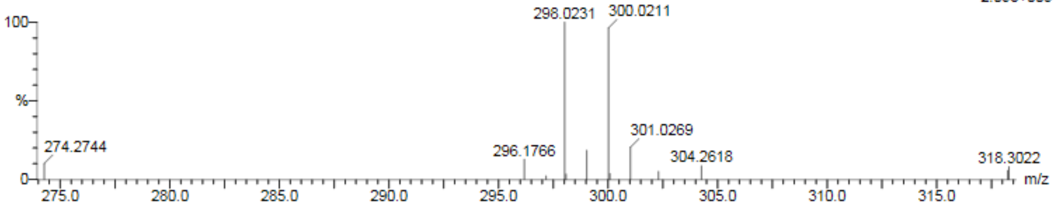

Minimum:				-1.5			
Maximum:		30.0	50.0	100.0			
Mass	Calc. Mass	mDa	PPM	DBE	i-FIT	i-FIT (Norm)	Formula
298.0231	298.0231	0.0	0.0	10.5	24.1	0.0	C16 H13 N Br

Elemental Composition Report

Single Mass Analysis

Tolerance = 50.0 PPM / DBE: min = -1.5, max = 100.0
Element prediction: Off
Number of isotope peaks used for i-FIT = 3

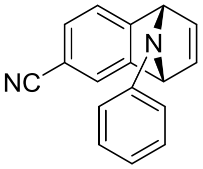

Monoisotopic Mass, Even Electron Ions
6 formula(e) evaluated with 1 results within limits (up to 1 best isotopic matches for each mass)
Elements Used:
C: 0-17 H: 0-100 N: 0-2

LM-WANG ECUST institute of Fine Chem 02-Jul-2017 14:01:35

WL-CHG-114 15 (0.280) Cm (15:16) 1: TOF MS ES+ 1.40e+003

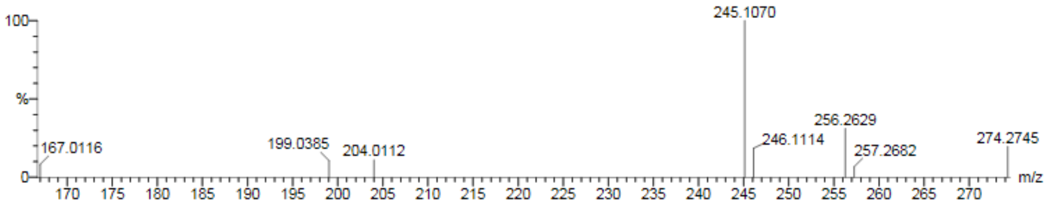

Minimum:				-1.5			
Maximum:		30.0	50.0	100.0			
Mass	Calc. Mass	mDa	PPM	DBE	i-FIT	i-FIT (Norm)	Formula
245.1070	245.1079	-0.9	-3.7	12.5	17.8	0.0	C17 H13 N2

Elemental Composition Report

Single Mass Analysis

Tolerance = 50.0 PPM / DBE: min = -1.5, max = 100.0
Element prediction: Off
Number of isotope peaks used for i-FIT = 3

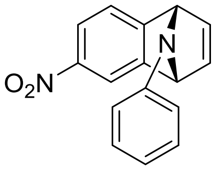

Monoisotopic Mass, Even Electron Ions
13 formula(e) evaluated with 1 results within limits (up to 1 best isotopic matches for each mass)
Elements Used:
C: 0-16 H: 0-50 N: 0-2 O: 0-2

LM-WANG ECUST institute of Fine Chem 27-Jun-2017 20:56:31

WL-CHG-115 11 (0.312) Cm (10:11) 1: TOF MS ES- 6.22e+003

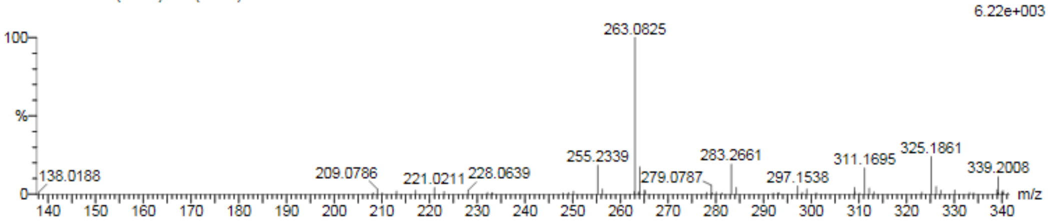

Minimum:				-1.5			
Maximum:		30.0	50.0	100.0			
Mass	Calc. Mass	mDa	PPM	DBE	i-FIT	i-FIT (Norm)	Formula
263.0825	263.0821	0.4	1.5	12.5	32.7	0.0	C16 H11 N2 O2

Single Mass Analysis
Tolerance = 50.0 PPM / DBE: min = -1.5, max = 100.0
Element prediction: Off
Number of isotope peaks used for i-FIT = 3

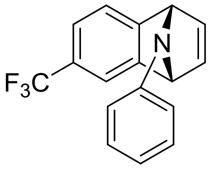

Monoisotopic Mass, Even Electron Ions
11 formula(e) evaluated with 1 results within limits (up to 1 best isotopic matches for each mass)
Elements Used:
C: 0-17 H: 0-100 N: 0-1 F: 0-3
LM-WANG ECUST institute of Fine Chem 02-Jul-2017
13:36:39
WL-CHG-117 29 (0.457) Cm (29:30) 1: TOF MS ES+
1.08e+004

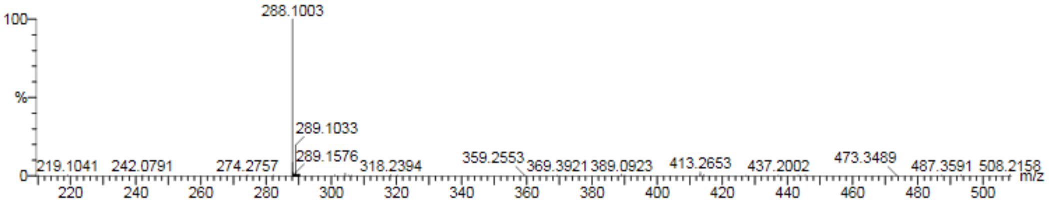

Minimum:				-1.5			
Maximum:		30.0	50.0	100.0			
Mass	Calc. Mass	mDa	PPM	DBE	i-FIT	i-FIT (Norm)	Formula
288.1003	288.1000	0.3	1.0	10.5	311.5	0.0	C17 H13 N F3

Elemental Composition Report

Page 1

Single Mass Analysis
Tolerance = 50.0 PPM / DBE: min = -1.5, max = 100.0
Element prediction: Off
Number of isotope peaks used for i-FIT = 3

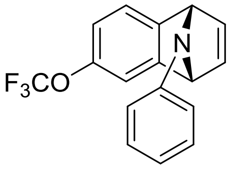

Monoisotopic Mass, Even Electron Ions
20 formula(e) evaluated with 1 results within limits (up to 1 best isotopic matches for each mass)
Elements Used:
C: 0-17 H: 0-100 N: 0-1 O: 0-1 F: 0-3
LM-WANG ECUST institute of Fine Chem 02-Jul-2017
13:46:59
WL-CHG-122 4 (0.149) Cm (2:4) 1: TOF MS ES+
9.69e+002

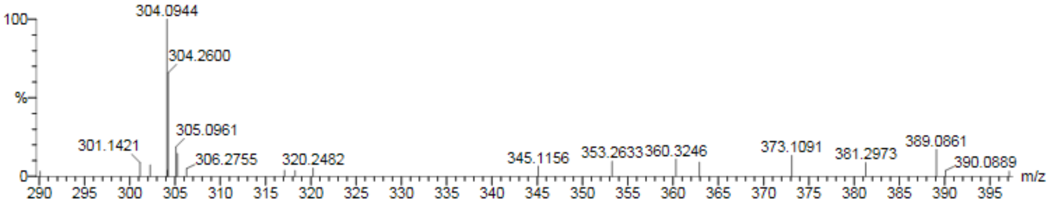

Minimum:				-1.5			
Maximum:		30.0	50.0	100.0			
Mass	Calc. Mass	mDa	PPM	DBE	i-FIT	i-FIT (Norm)	Formula
304.0944	304.0949	-0.5	-1.6	10.5	54.7	0.0	C17 H13 N O F3

Elemental Composition Report

Single Mass Analysis

Tolerance = 50.0 PPM / DBE: min = -1.5, max = 100.0
Element prediction: Off
Number of isotope peaks used for i-FIT = 3

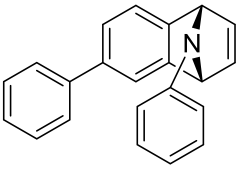

Monoisotopic Mass, Even Electron Ions
5 formula(e) evaluated with 1 results within limits (up to 1 best isotopic matches for each mass)
Elements Used:
C: 0-22 H: 0-100 N: 0-1

LM-WANG ECUST institute of Fine Chem 02-Jul-2017 14:05:47

WL-CHG-121 34 (0.509) Cm (32:36) 1: TOF MS ES+ 8.42e+003

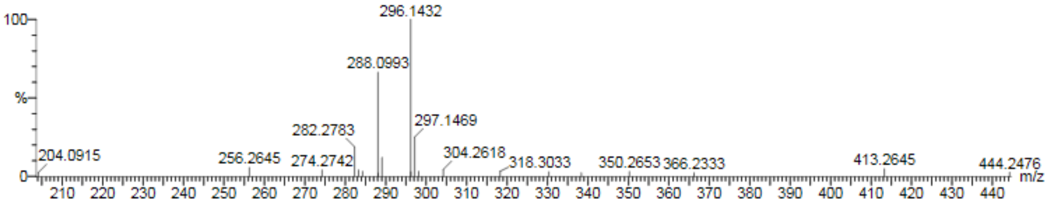

Minimum:				-1.5			
Maximum:		30.0	50.0	100.0			
Mass	Calc. Mass	mDa	PPM	DBE	i-FIT	i-FIT (Norm)	Formula
296.1432	296.1439	-0.7	-2.4	14.5	18.0	0.0	C22 H18 N

Elemental Composition Report

Single Mass Analysis

Tolerance = 50.0 PPM / DBE: min = -1.5, max = 100.0
Element prediction: Off
Number of isotope peaks used for i-FIT = 3

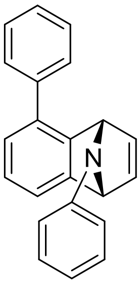

Monoisotopic Mass, Even Electron Ions
5 formula(e) evaluated with 1 results within limits (up to 1 best isotopic matches for each mass)
Elements Used:
C: 0-22 H: 0-100 N: 0-1

LM-WANG ECUST institute of Fine Chem 02-Jul-2017 13:57:32

WL-CHG-120 28 (0.446) Cm (28:31) 1: TOF MS ES+ 8.05e+003

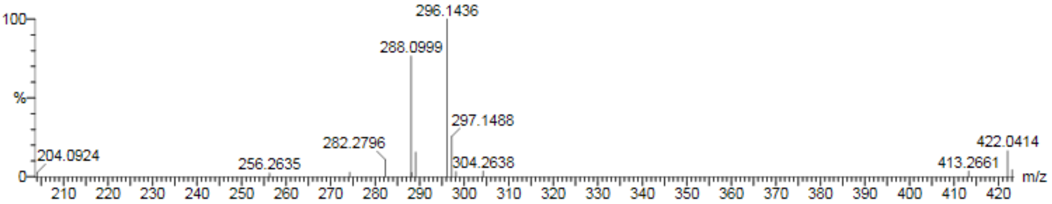

Minimum:				-1.5			
Maximum:		30.0	50.0	100.0			
Mass	Calc. Mass	mDa	PPM	DBE	i-FIT	i-FIT (Norm)	Formula
296.1436	296.1439	-0.3	-1.0	14.5	8.6	0.0	C22 H18 N

Elemental Composition Report

Single Mass Analysis

Tolerance = 50.0 PPM / DBE: min = -1.5, max = 100.0
Element prediction: Off
Number of isotope peaks used for i-FIT = 3

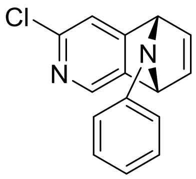

Monoisotopic Mass, Even Electron Ions
4 formula(e) evaluated with 1 results within limits (up to 1 best isotopic matches for each mass)
Elements Used:
C: 0-15 H: 0-10 N: 0-2 Cl: 0-1

LM-WANG ECUST institute of Fine Chem 30-Jun-2017 12:11:34

WL-CHG-116 31 (0.752) Cm (31:32) 1: TOF MS ES- 9.17e+003

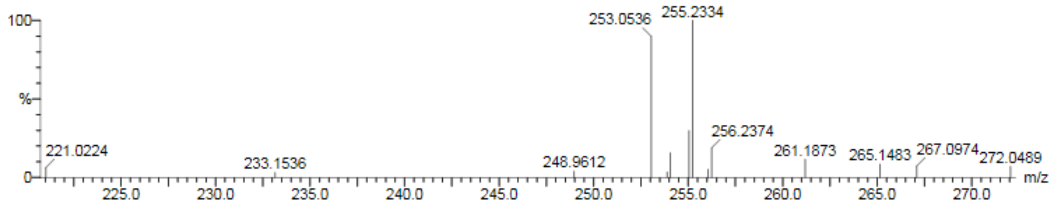

Minimum:				-1.5			
Maximum:		30.0	50.0	100.0			
Mass	Calc. Mass	mDa	PPM	DBE	i-FIT	i-FIT (Norm)	Formula
253.0536	253.0533	0.3	1.2	11.5	38.2	0.0	C15 H10 N2 Cl

Elemental Composition Report

Page 1

Single Mass Analysis

Tolerance = 50.0 PPM / DBE: min = -1.5, max = 100.0
Element prediction: Off
Number of isotope peaks used for i-FIT = 3

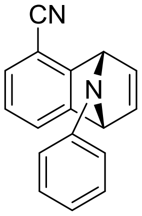

Monoisotopic Mass, Even Electron Ions
1 formula(e) evaluated with 1 results within limits (up to 1 best isotopic matches for each mass)
Elements Used:
C: 0-17 H: 0-13 N: 0-2

LM-WANG ECUST institute of Fine Chem 28-Jun-2017 21:57:35

WL-CHG-119 31 (0.478) Cm (30:33) 1: TOF MS ES+ 1.52e+005

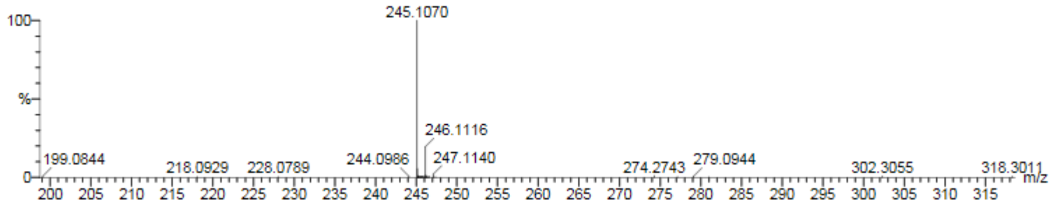

Minimum:				-1.5			
Maximum:		30.0	50.0	100.0			
Mass	Calc. Mass	mDa	PPM	DBE	i-FIT	i-FIT (Norm)	Formula
245.1070	245.1079	-0.9	-3.7	12.5	373.6	0.0	C17 H13 N2

Elemental Composition Report

Single Mass Analysis
Tolerance = 50.0 PPM / DBE: min = -1.5, max = 100.0
Element prediction: Off
Number of isotope peaks used for i-FIT = 3

Monoisotopic Mass, Even Electron Ions
13 formula(e) evaluated with 1 results within limits (up to 1 best isotopic matches for each mass)
Elements Used:
C: 0-16 H: 0-50 N: 0-1 F: 0-2

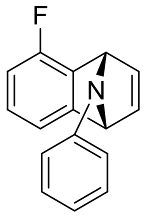

LM-WANG ECUST institute of Fine Chem 09-Jul-2017 13:29:46

WL-CHG-124 7 (0.299) Cm (6:8) 1: TOF MS ES+ 1.31e+003

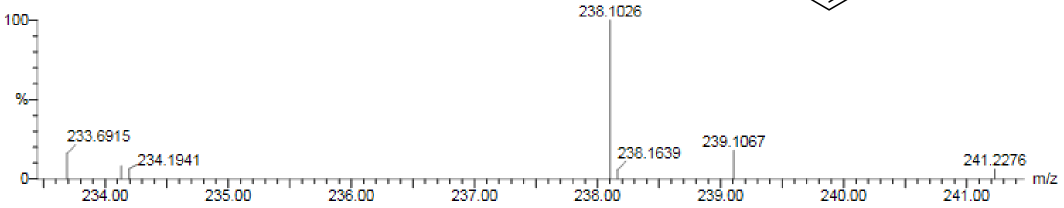

Minimum:				-1.5			
Maximum:		30.0	50.0	100.0			
Mass	Calc. Mass	mDa	PPM	DBE	i-FIT	i-FIT (Norm)	Formula
238.1026	238.1032	-0.6	-2.5	10.5	25.1	0.0	C16 H13 N F

Elemental Composition Report

Page 1

Single Mass Analysis
Tolerance = 50.0 PPM / DBE: min = -1.5, max = 100.0
Element prediction: Off
Number of isotope peaks used for i-FIT = 3

Monoisotopic Mass, Even Electron Ions
5 formula(e) evaluated with 1 results within limits (up to 1 best isotopic matches for each mass)
Elements Used:
C: 0-17 H: 0-50 N: 0-1

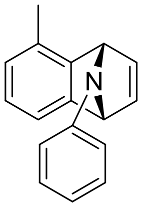

LM-WANG ECUST institute of Fine Chem 09-Jul-2017 13:39:43

WL-CHG-125 12 (0.446) Cm (11:12) 1: TOF MS ES+ 3.24e+003

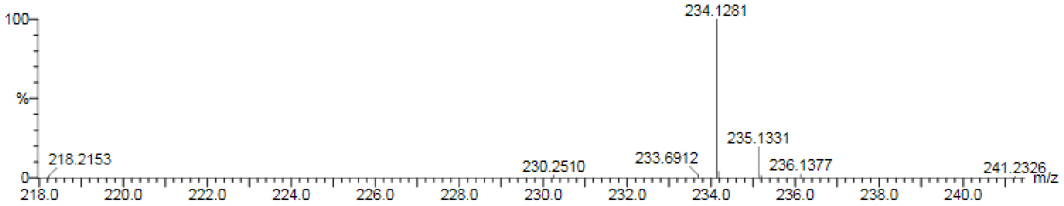

Minimum:				-1.5			
Maximum:		30.0	50.0	100.0			
Mass	Calc. Mass	mDa	PPM	DBE	i-FIT	i-FIT (Norm)	Formula
234.1281	234.1283	-0.2	-0.9	10.5	24.3	0.0	C17 H16 N

Elemental Composition Report

Single Mass Analysis
Tolerance = 50.0 PPM / DBE: min = -1.5, max = 100.0
Element prediction: Off
Number of isotope peaks used for i-FIT = 3

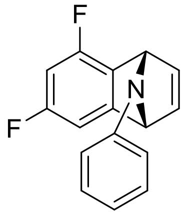

Monoisotopic Mass, Even Electron Ions
25 formula(e) evaluated with 1 results within limits (up to 1 best isotopic matches for each mass)
Elements Used:
C: 0-16 H: 0-50 N: 0-3 F: 0-2

LM-WANG ECUST institute of Fine Chem 09-Jul-2017 13:09:32

WL-CHG-123 47 (1.529) Cm (47:48) 1: TOF MS ES+ 1.48e+003

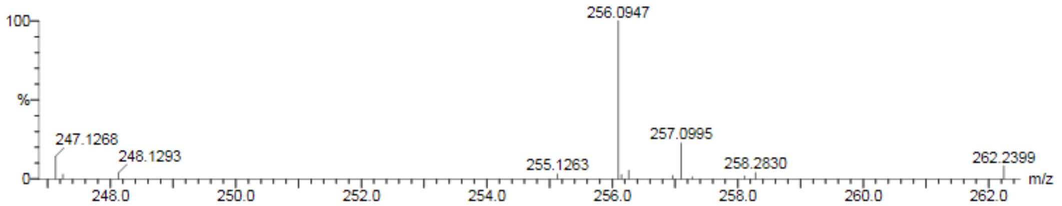

	Mass	Calc. Mass	mDa	PPM	DBE	i-FIT	i-FIT (Norm)	Formula
Minimum:					-1.5			
Maximum:			30.0	50.0	100.0			
	256.0947	256.0938	0.9	3.5	10.5	46.6	0.0	C16 H12 N F2

Elemental Composition Report

Single Mass Analysis
Tolerance = 30.0 PPM / DBE: min = -1.5, max = 100.0
Element prediction: Off
Number of isotope peaks used for i-FIT = 3

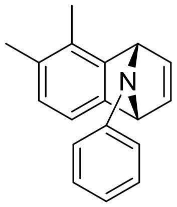

Monoisotopic Mass, Even Electron Ions
1 formula(e) evaluated with 1 results within limits (up to 1 best isotopic matches for each mass)
Elements Used:
C: 0-18 H: 0-23 N: 0-1

LM-WANG ECUST institute of Fine Chem 22-Jul-2017 12:56:26

WL-CHG-126 17 (0.620) Cm (17:18) 1: TOF MS ES+ 8.17e+003

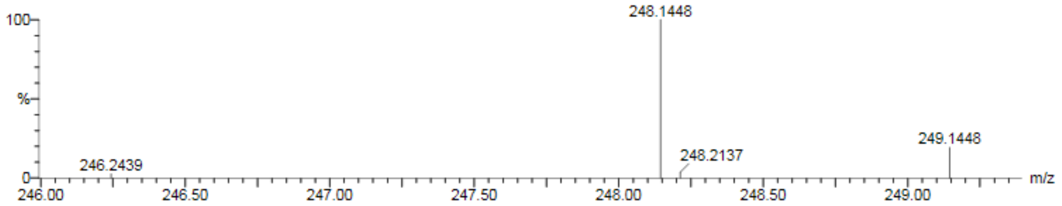

	Mass	Calc. Mass	mDa	PPM	DBE	i-FIT	i-FIT (Norm)	Formula
Minimum:					-1.5			
Maximum:			30.0	30.0	100.0			
	248.1448	248.1439	0.9	3.6	10.5	39.7	0.0	C18 H18 N

Single Mass Analysis

Tolerance = 30.0 PPM / DBE: min = -1.5, max = 100.0
Element prediction: Off
Number of isotope peaks used for i-FIT = 3

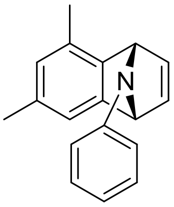

Monoisotopic Mass, Even Electron Ions
1 formula(e) evaluated with 1 results within limits (up to 1 best isotopic matches for each mass)
Elements Used:
C: 0-18 H: 0-23 N: 0-1

LM-WANG ECUST institute of Fine Chem 22-Jul-2017 12:52:02

WL-CHG-127 5 (0.251) Cm (4:6) 1: TOF MS ES+ 1.20e+004

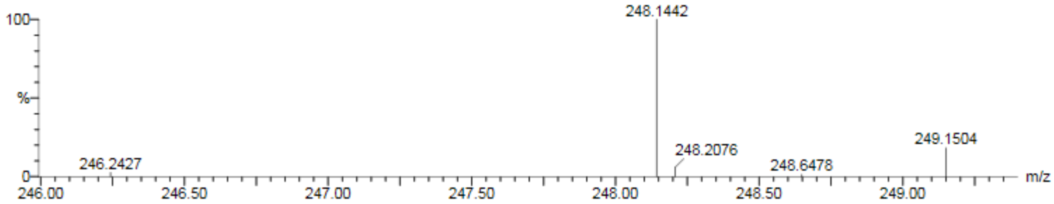

Minimum:				-1.5			
Maximum:		30.0	30.0	100.0			
Mass	Calc. Mass	mDa	PPM	DBE	i-FIT	i-FIT (Norm)	Formula
248.1442	248.1439	0.3	1.2	10.5	52.3	0.0	C18 H18 N

Elemental Composition Report
Page 1

Single Mass Analysis

Tolerance = 50.0 PPM / DBE: min = -1.5, max = 100.0
Element prediction: Off
Number of isotope peaks used for i-FIT = 3

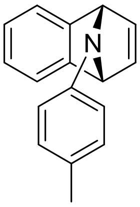

Monoisotopic Mass, Even Electron Ions
5 formula(e) evaluated with 1 results within limits (up to 1 best isotopic matches for each mass)
Elements Used:
C: 0-17 H: 0-50 N: 0-1

LM-WANG ECUST institute of Fine Chem 09-Jul-2017 13:35:03

WL-CHG-160 27 (0.917) Cm (26:28) 1: TOF MS ES+ 4.05e+003

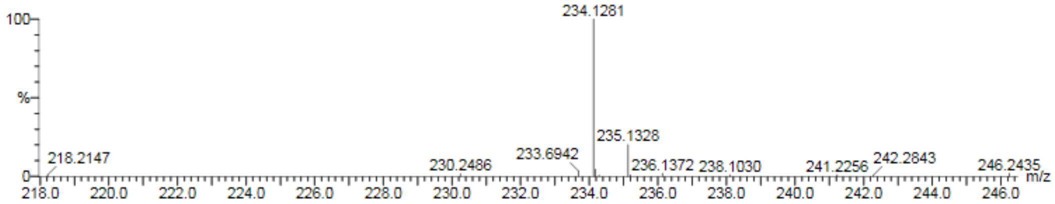

Minimum:				-1.5			
Maximum:		30.0	50.0	100.0			
Mass	Calc. Mass	mDa	PPM	DBE	i-FIT	i-FIT (Norm)	Formula
234.1281	234.1283	-0.2	-0.9	10.5	30.9	0.0	C17 H16 N

Elemental Composition Report

Single Mass Analysis

Tolerance = 50.0 PPM / DBE: min = -1.5, max = 100.0
Element prediction: Off
Number of isotope peaks used for i-FIT = 3

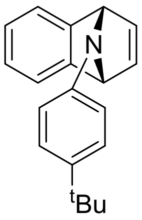

Monoisotopic Mass, Even Electron Ions
3 formula(e) evaluated with 1 results within limits (up to 1 best isotopic matches for each mass)
Elements Used:
C: 0-20 H: 0-25 N: 0-2

LM-WANG ECUST institute of Fine Chem

WL-CHG-162 5 (0.251) Cm (5:6)

08-Jul-2017
16:44:59
1: TOF MS ES+
9.81e+003

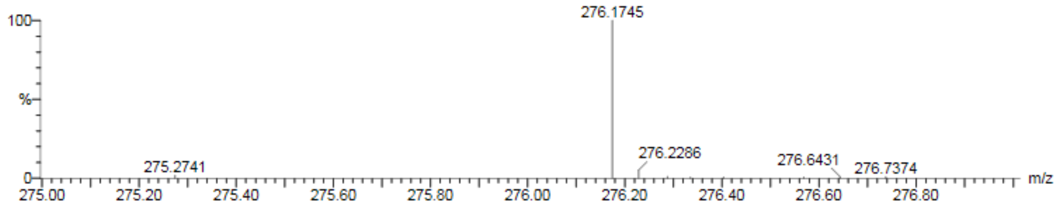

Minimum:				-1.5			
Maximum:		30.0	50.0	100.0			
Mass	Calc. Mass	mDa	PPM	DBE	i-FIT	i-FIT (Norm)	Formula
276.1745	276.1752	-0.7	-2.5	10.5	92.1	0.0	C20 H22 N

Elemental Composition Report

Single Mass Analysis

Tolerance = 50.0 PPM / DBE: min = -1.5, max = 100.0
Element prediction: Off
Number of isotope peaks used for i-FIT = 3

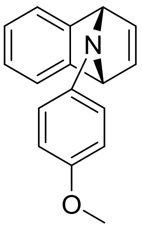

Monoisotopic Mass, Even Electron Ions
1 formula(e) evaluated with 1 results within limits (up to 1 best isotopic matches for each mass)
Elements Used:
C: 0-17 H: 0-20 N: 0-1 O: 0-1

LM-WANG ECUST institute of Fine Chem

WL-ZZA-161 20 (0.348) Cm (20:23)

11-Jul-2017
22:43:31
1: TOF MS ES+
4.73e+003

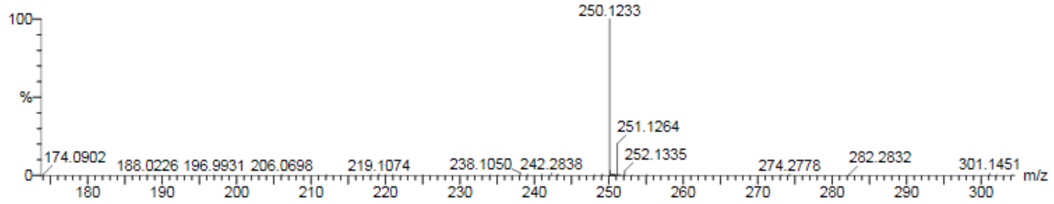

Minimum:				-1.5			
Maximum:		30.0	50.0	100.0			
Mass	Calc. Mass	mDa	PPM	DBE	i-FIT	i-FIT (Norm)	Formula
250.1233	250.1232	0.1	0.4	10.5	124.5	0.0	C17 H16 N O

Elemental Composition Report

Page 1

Single Mass Analysis

Tolerance = 50.0 PPM / DBE: min = -1.5, max = 100.0
Element prediction: Off
Number of isotope peaks used for i-FIT = 3

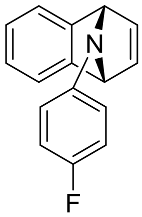

Monoisotopic Mass, Even Electron Ions
2 formula(e) evaluated with 1 results within limits (up to 1 best isotopic matches for each mass)
Elements Used:
C: 0-16 H: 0-20 N: 0-1 F: 0-1

LM-WANG ECUST institute of Fine Chem 11-Jul-2017 22:33:32

WL-ZZA-163 37 (0.560) Cm (37:39) 1: TOF MS ES+ 5.97e+003

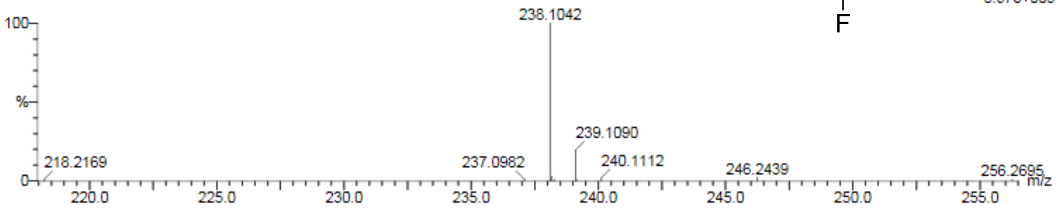

Minimum:				-1.5			
Maximum:		30.0	50.0	100.0			
Mass	Calc. Mass	mDa	PPM	DBE	i-FIT	i-FIT (Norm)	Formula
238.1042	238.1032	1.0	4.2	10.5	32.8	0.0	C16 H13 N F

Elemental Composition Report

Page 1

Single Mass Analysis

Tolerance = 50.0 PPM / DBE: min = -1.5, max = 100.0
Element prediction: Off
Number of isotope peaks used for i-FIT = 3

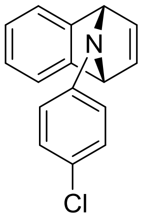

Monoisotopic Mass, Even Electron Ions
5 formula(e) evaluated with 1 results within limits (up to 1 best isotopic matches for each mass)
Elements Used:
C: 0-16 H: 0-35 N: 0-1 Cl: 0-1

LM-WANG ECUST institute of Fine Chem 11-Jul-2017 23:11:21

WL-CHG-164 19 (0.337) Cm (18:19) 1: TOF MS ES+ 1.75e+003

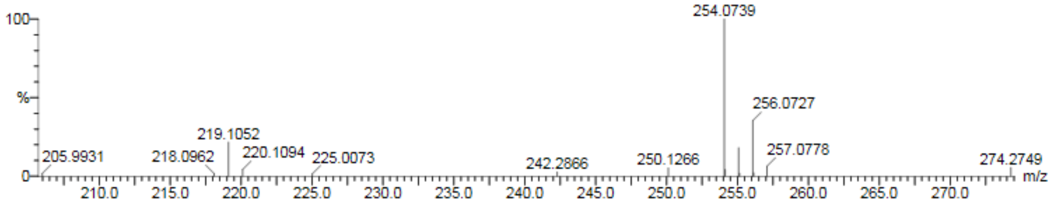

Minimum:				-1.5			
Maximum:		30.0	50.0	100.0			
Mass	Calc. Mass	mDa	PPM	DBE	i-FIT	i-FIT (Norm)	Formula
254.0739	254.0737	0.2	0.8	10.5	29.9	0.0	C16 H13 N Cl

Single Mass Analysis

Tolerance = 50.0 PPM / DBE: min = -1.5, max = 100.0
Element prediction: Off
Number of isotope peaks used for i-FIT = 3

Monoisotopic Mass, Even Electron Ions
5 formula(e) evaluated with 1 results within limits (up to 1 best isotopic matches for each mass)
Elements Used:
C: 0-16 H: 0-35 N: 0-1 Br: 0-1

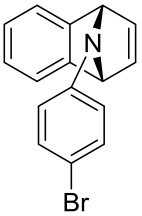

LM-WANG ECUST institute of Fine Chem 11-Jul-2017 23:14:23

WL-CHG-165 23 (0.378) Cm (23:25) 1: TOF MS ES+ 3.91e+003

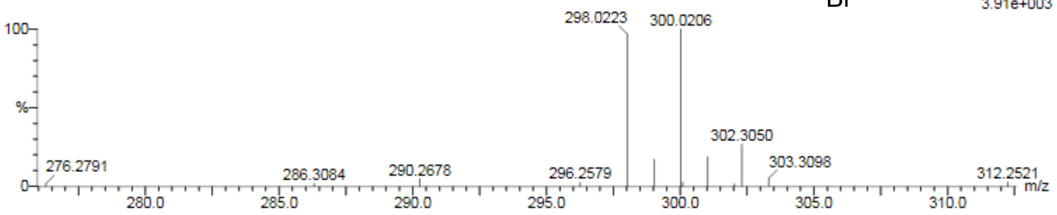

Minimum:				-1.5			
Maximum:		30.0	50.0	100.0			
Mass	Calc. Mass	mDa	PPM	DBE	i-FIT	i-FIT (Norm)	Formula
298.0223	298.0231	-0.8	-2.7	10.5	16.1	0.0	C16 H13 N Br

Elemental Composition Report Page 1

Single Mass Analysis

Tolerance = 30.0 PPM / DBE: min = -1.5, max = 100.0
Element prediction: Off
Number of isotope peaks used for i-FIT = 3

Monoisotopic Mass, Even Electron Ions
6 formula(e) evaluated with 1 results within limits (up to 1 best isotopic matches for each mass)
Elements Used:
C: 0-17 H: 0-15 N: 0-1 F: 0-3

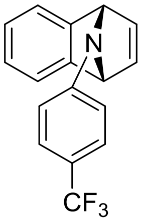

LM-WANG ECUST institute of Fine Chem 22-Jul-2017 12:49:21

WL-CHG-166 13 (0.496) Cm (13:15) 1: TOF MS ES+ 5.30e+002

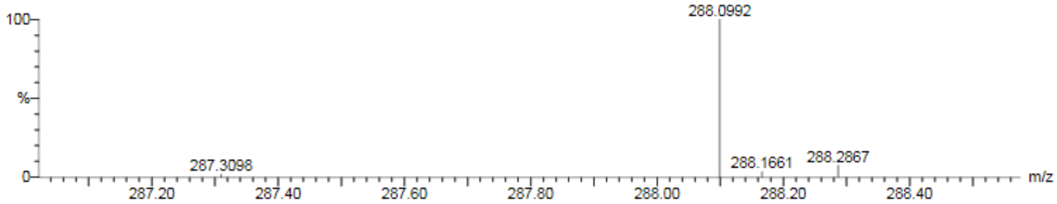

Minimum:				-1.5			
Maximum:		30.0	30.0	100.0			
Mass	Calc. Mass	mDa	PPM	DBE	i-FIT	i-FIT (Norm)	Formula
288.0992	288.1000	-0.8	-2.8	10.5	35.0	0.0	C17 H13 N F3

Elemental Composition Report

Page 1

Single Mass Analysis

Tolerance = 30.0 PPM / DBE: min = -1.5, max = 100.0
Element prediction: Off
Number of isotope peaks used for i-FIT = 3

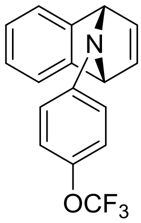

Monoisotopic Mass, Even Electron Ions
13 formula(e) evaluated with 1 results within limits (up to 1 best isotopic matches for each mass)
Elements Used:
C: 0-18 H: 0-23 N: 0-1 O: 0-1 F: 0-3

LM-WANG ECUST institute of Fine Chem 22-Jul-2017 12:54:20

WL-CHG-167 9 (0.374) Cm (9:10) 1: TOF MS ES+ 3.31e+003

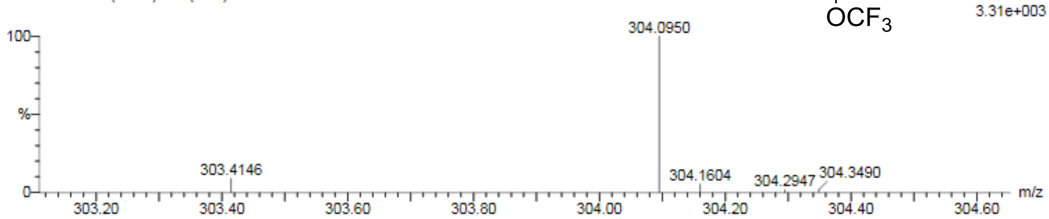

Minimum:				-1.5			
Maximum:		30.0	30.0	100.0			
Mass	Calc. Mass	mDa	PPM	DBE	i-FIT	i-FIT (Norm)	Formula
304.0950	304.0949	0.1	0.3	10.5	52.4	0.0	C17 H13 N O F3

Elemental Composition Report

Page 1

Single Mass Analysis

Tolerance = 30.0 PPM / DBE: min = -1.5, max = 100.0
Element prediction: Off
Number of isotope peaks used for i-FIT = 3

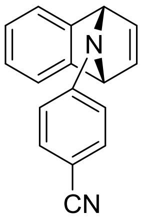

Monoisotopic Mass, Even Electron Ions
1 formula(e) evaluated with 1 results within limits (up to 1 best isotopic matches for each mass)
Elements Used:
C: 0-17 H: 0-13 N: 0-2

LM-WANG ECUST institute of Fine Chem 13-Sep-2017 23:08:32

WL-CHG-1711 104 (1.366) Cm (102:107) 1: TOF MS ES+ 1.18e+004

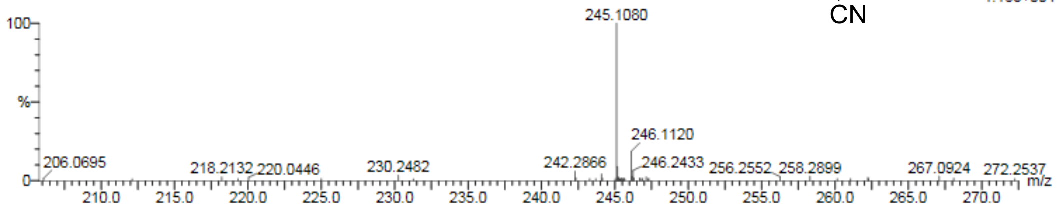

Minimum:				-1.5			
Maximum:		30.0	30.0	100.0			
Mass	Calc. Mass	mDa	PPM	DBE	i-FIT	i-FIT (Norm)	Formula
245.1080	245.1079	0.1	0.4	12.5	197.9	0.0	C17 H13 N2

Elemental Composition Report

Single Mass Analysis

Tolerance = 30.0 PPM / DBE: min = -1.5, max = 100.0
Element prediction: Off
Number of isotope peaks used for i-FIT = 3

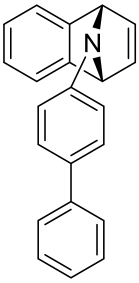

Monoisotopic Mass, Even Electron Ions
12 formula(e) evaluated with 1 results within limits (up to 1 best isotopic matches for each mass)
Elements Used:
C: 0-27 H: 0-81 N: 0-2

LM-WANG ECUST institute of Fine Chem 20-Jul-2017 18:18:14

WL-CHG-168 10 (0.227) Cm (10:12) 1: TOF MS ES+ 5.14e+002

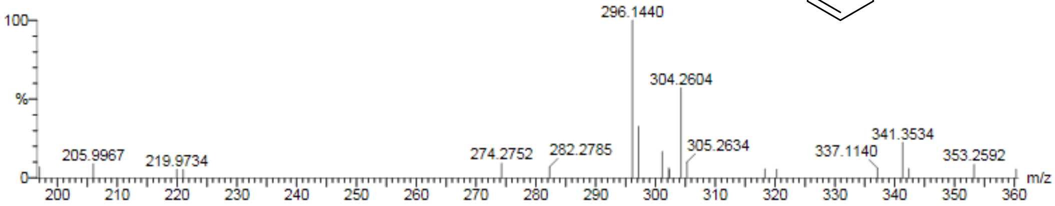

Minimum:				-1.5			
Maximum:		30.0	30.0	100.0			
Mass	Calc. Mass	mDa	PPM	DBE	i-FIT	i-FIT (Norm)	Formula
296.1440	296.1439	0.1	0.3	14.5	15.9	0.0	C22 H18 N

Elemental Composition Report

Single Mass Analysis

Tolerance = 30.0 PPM / DBE: min = -1.5, max = 100.0
Element prediction: Off
Number of isotope peaks used for i-FIT = 3

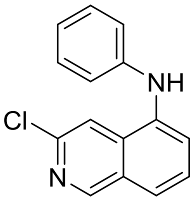

Monoisotopic Mass, Even Electron Ions
73 formula(e) evaluated with 3 results within limits (up to 1 best isotopic matches for each mass)
Elements Used:
C: 0-38 H: 0-82 N: 0-4 Cl: 0-4

LM-WANG ECUST institute of Fine Chem 22-Sep-2017 14:40:59

WL-CHG-182 192 (2.440) Cm (188:194) 1: TOF MS ES+ 1.88e+003

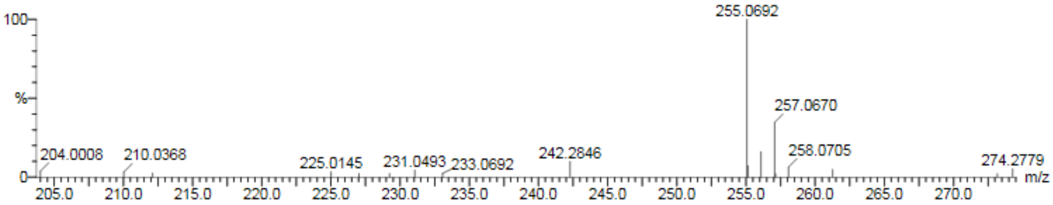

Minimum:				-1.5			
Maximum:		30.0	30.0	100.0			
Mass	Calc. Mass	mDa	PPM	DBE	i-FIT	i-FIT (Norm)	Formula
255.0692	255.0689	0.3	1.2	10.5	22.6	0.0	C15 H12 N2 Cl

Elemental Composition Report

Single Mass Analysis
Tolerance = 30.0 PPM / DBE: min = -1.5, max = 100.0
Element prediction: Off
Number of isotope peaks used for i-FIT = 3

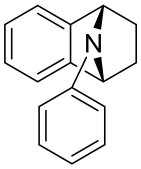

Monoisotopic Mass, Even Electron Ions
19 formula(e) evaluated with 1 results within limits (up to 1 best isotopic matches for each mass)
Elements Used:
C: 0-38 H: 0-82 N: 0-4

LM-WANG ECUST institute of Fine Chem 22-Sep-2017 14:47:42

WL-CHG-1861 48 (0.686) Cm (48:50) 1: TOF MS ES+ 9.67e+002

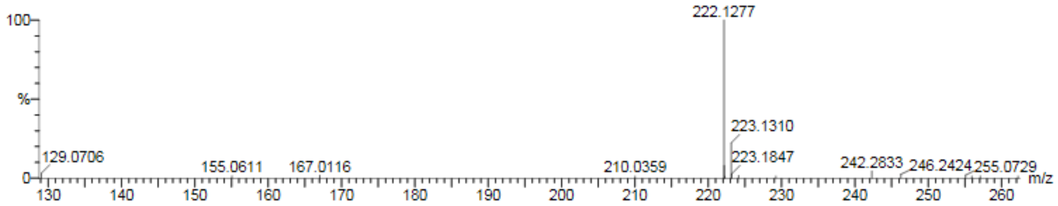

Minimum:				-1.5			
Maximum:		30.0	30.0	100.0			
Mass	Calc. Mass	mDa	PPM	DBE	i-FIT	i-FIT (Norm)	Formula
222.1277	222.1283	-0.6	-2.7	9.5	24.4	0.0	C16 H16 N
